# Discovery of Pre-Clinical Candidate **VU6008055**/**AF98943**: A Highly Selective, Orally Bioavailable, and Structurally Distinct Tricyclic M_4_ Muscarinic Acetylcholine Receptor Positive Allosteric Modulator (PAM) with Robust In Vivo Efficacy

**DOI:** 10.1021/acschemneuro.5c00277

**Published:** 2025-05-26

**Authors:** Julie L. Engers, Sean R. Bollinger, Alison R. Gregro, Rory A. Capstick, Paul K. Spearing, Madeline F. Long, James C. Tarr, Katherine J. Watson, Sichen Chang, Vincent B. Luscombe, Alice L. Rodriguez, Hyekyung P. Cho, Aidong Qi, Colleen M. Niswender, Michael Bubser, Robert W. Gould, William Hudson Robb, Nellie Byun, John Gore, Carrie K. Jones, Morten S. Thomsen, Thomas M. Bridges, Olivier Boutaud, P. Jeffrey Conn, Darren W. Engers, Craig W. Lindsley, Kayla J. Temple

**Affiliations:** † Warren Center for Neuroscience Drug Discovery, 5718 Vanderbilt University, Nashville, Tennessee 37232, United States; ‡ Department of Pharmacology, 12327 Vanderbilt University School of Medicine, Nashville, Tennessee 37232, United States; § Department of Chemistry, Vanderbilt University, Nashville, Tennessee 37232, United States; ∥ Department of Biochemistry, Vanderbilt University, Nashville, Tennessee 37232, United States; ⊥ Vanderbilt Kennedy Center, Vanderbilt University School of Medicine, Nashville, Tennessee 37232, United States; # Vanderbilt Brain Institute, Vanderbilt University School of Medicine, Nashville, Tennessee 37232, United States; ¶ Vanderbilt University Institute of Imaging Science, Vanderbilt University School of Medicine, Nashville, Tennessee 37232, United States; ∇ Department of Radiology and Radiological Sciences, 12328 Vanderbilt University Medical Center, Nashville, Tennessee 37232, United States; ○ Department of Biomedical Engineering, Vanderbilt University, Nashville, Tennessee 37232, United States; ⧫ Neuroscience Research, 8134 H. Lundbeck A/S, Valby DK-2500, Denmark

**Keywords:** muscarinic acetylcholine receptor (mAChR), M_4_, positive allosteric modulator (PAM), structure–activity relationship (SAR), schizophrenia, Parkinson’s disease, Alzheimer’s disease, VU6008055

## Abstract

Herein, we report the structure–activity relationship to develop novel tricyclic M_4_ positive allosteric modulator scaffolds with improved pharmacological properties. This endeavor involved modifying a 5-amino-3,4-dimethylthieno­[2,3-*c*]­pyridazine-6-carboxamide core via a “tie-back” strategy to discover a novel tricyclic 3,4-dimethylpyrimido­[4′,5′:4,5]­thieno­[2,3-*c*]­pyridazine core. From this exercise, **VU6008055**/**AF98943** was identified as a preclinical candidate, which displays low nanomolar potency against both human and rat M_4_. Moreover, **VU6008055** is highly brain penetrant, has an overall superior pharmacological and DMPK profile to previously reported M_4_ PAMs, and demonstrates efficacy in preclinical models of antipsychotic-like activity.

## Graphical abstract

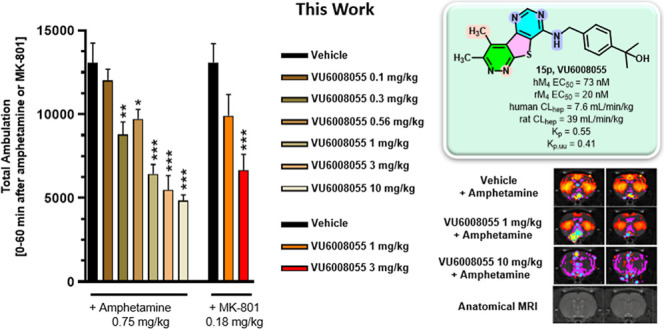

## Introduction

Muscarinic acetylcholine receptor subtype 4 (M_4_) positive allosteric modulators (PAMs) continue to be important drug targets as novel treatments for various neurological disorders such as Parkinson’s disease,^1^ Huntington’s disease,^2^ and schizophrenia (both the positive and negative symptom clusters).^3–7^ Historical M_4_ PAM chemotypes contain a key pharmacophore (a β-amino carboxamide moiety, circled in **4**, Figure 1), which has been linked to potency discrepancies across species, poor compound solubility, as well as low brain exposure due to varying degrees of P-glycoprotein (P-gp) efflux.^8–17^ Consequently, efforts in the field have shifted toward the development of novel M_4_ PAM chemotypes with the goal of improving drug-like properties.^18–32^ In the early 1990s, Eli Lilly & Co., in collaboration with Novo Nordisk, provided further target validation of the muscarinic cholinergic system as a treatment for the psychosis and behavioral disturbances observed in both Alzheimer’s disease and schizophrenia patients with the development of xanomeline (an M_1_/M_4_ preferring agonist).^27,28^ However, xanomeline’s clinical development was ultimately halted due to the lack of selectivity among receptor subtypes, which resulted in peripherally mediated cholinergic side effects. To circumvent these adverse events, Karuna Therapeutics (acquired by Bristol Myers Squibb) developed KarXT; recently, a New Drug Application was approved by the FDA.^29^ KarXT (Cobenfy) is a treatment in which xanomeline is coadministered with a pan-selective, peripherally restricted muscarinic acetylcholine receptor antagonist (trospium chloride) in order to minimize the cholinergic adverse events associated when xanomeline is administered alone (Figure 2).^30^ More recently, a selective M_4_ PAM demonstrated robust efficacy in preclinical models of antipsychotic-like activity and enhancement of cognition, while also displaying none of the adverse cholinergic-related side effects previously observed with xanomeline.^12^ These data suggest that receptor-subtype-selective M_4_ PAMs could be a strategy to improve safety and tolerability profiles. One such selective M_4_ PAM, CVL-231, is currently undergoing clinical trials for the treatment of schizophrenia as an adjunct treatment (Figure 2).^32–38^

**Figure 1:**
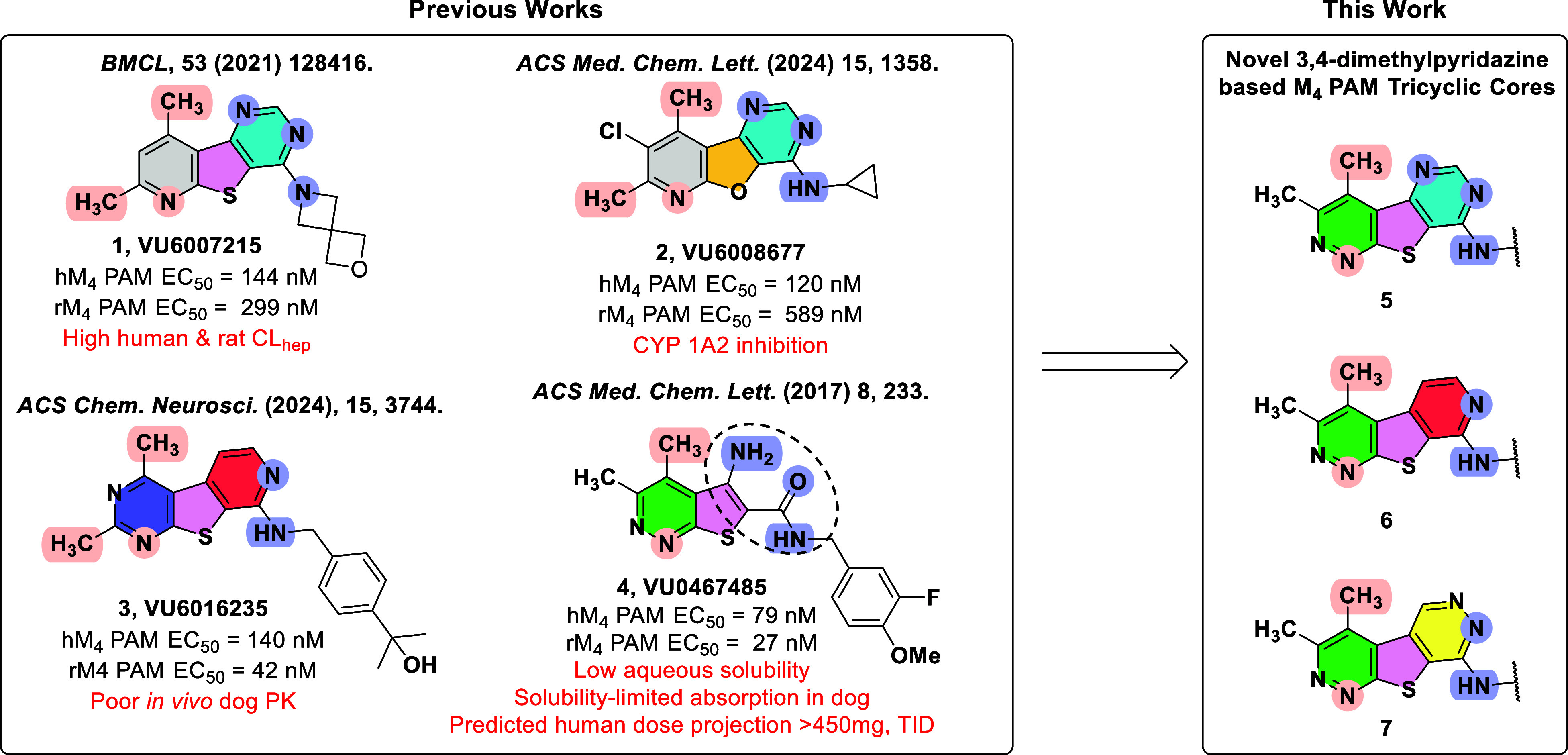
Discovery of novel, structurally distinct tricyclic cores as M_4_ PAMs. Utilization of a “tie-back” strategy to mask the β-amino carboxamide moiety (dotted circle) of **VU0467485** (**4**) revealed three unique M_4_ PAM tricyclic chemotypes: 3,4-dimethylpyrimido­[4′,5′:4,5]­thieno­[2,3-*c*]­pyridazine core **5**, 3,4-dimethylpyrido­[4′,3′:4,5]­thieno­[2,3-*c*]­pyridazine core **6**, and 3,4-dimethylthieno­[2,3-*c*:4,5-*d*′]­dipyridazine core **7**.

**Figure 2:**
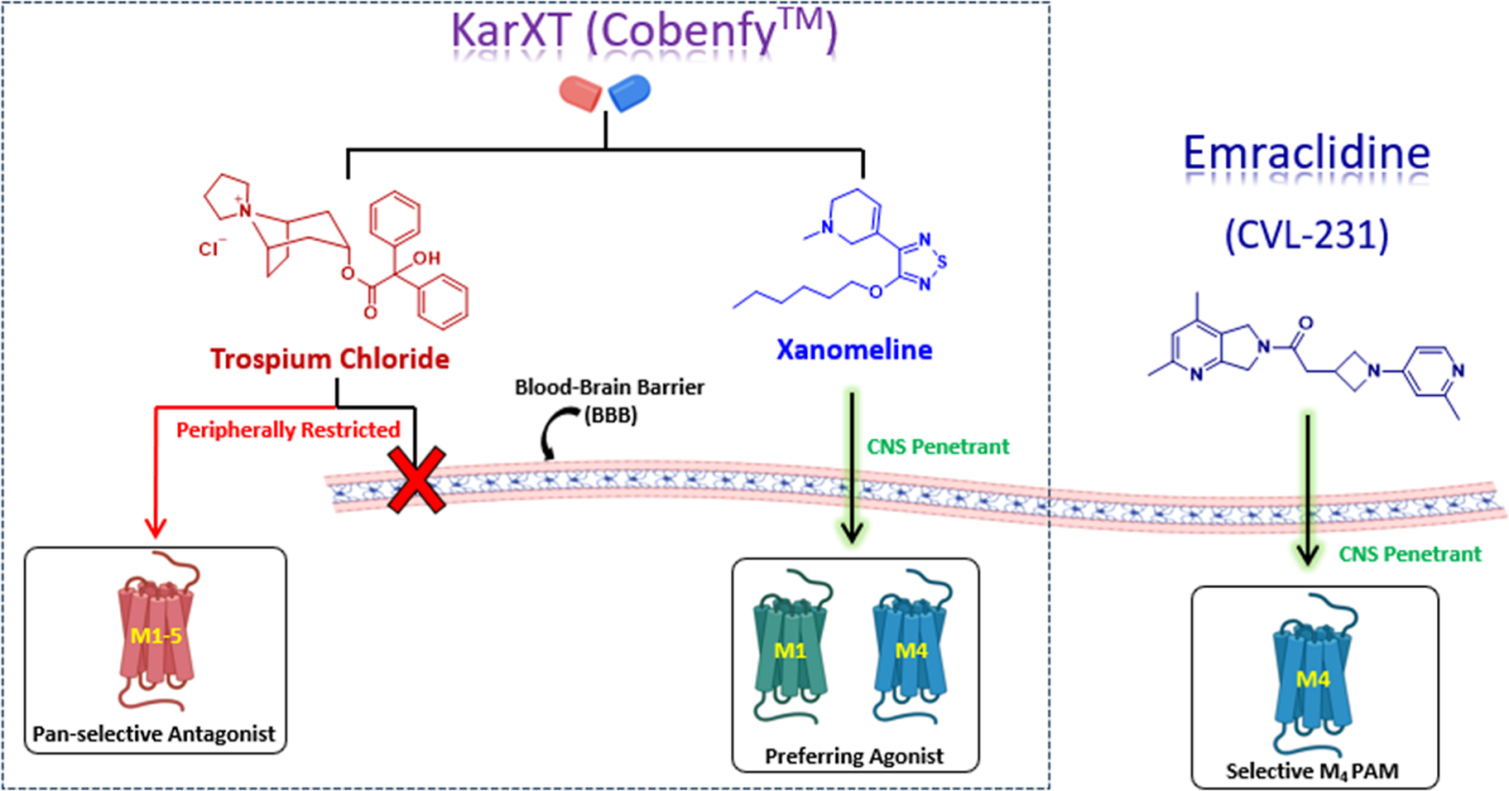
Structures of clinically advanced M_4_-targeting therapeutics.

Our lab recently revealed several structurally distinct tricyclic M_4_ PAM chemotypes in which the β-amino carboxamide moiety was masked via a “tie-back” strategy (Figure 1, 1–3).^21–24^ While this strategy afforded novel chemical matter, the previous scaffolds suffered from either high predicted human and rat hepatic clearance (**VU6007215**), CYP 1A2 inhibition (**VU6008677**), or poor in vivo higher species pharmacokinetics (**VU6016235**). To overcome these challenges and identify additional unique M_4_ PAM chemotypes, we extended our “tie-back” strategy to incorporate another of our lab’s early lead M_4_ PAM compounds, **VU0467485** (**4**).^14^ When designing this new scaffold, we elected to keep the 3,4-dimethylthieno­[2,3-*c*]­pyridazine core intact while first masking the β-amino carboxamide as a pyrimidine moiety (core **5**), a strategy utilized in the generation of **VU6007215** (**1**).^22^ This resulted in the discovery of a novel M_4_ PAM chemotype containing a 3,4-dimethylpyrimido­[4′,5′:4,5]-thieno­[2,3-*c*]­pyridazine core (**5**). Further exploration resulted in the discovery of two additional tricyclic M_4_ PAM chemotypes: 3,4-dimethylpyrido­[4′,3′:4,5]­thieno­[2,3-*c*]­pyridazine core **6** and 3,4-dimethylthieno­[2,3-c:4,5-*d*′]­dipyridazine core **7**. This body of work details the development of these novel M_4_ PAM chemotypes.

## Results and Discussion

### Synthesis and SAR

The synthesis of analogs with core **5** began with the commercially available mercaptan **8**, which was condensed with various α-bromo or chloro ketones to afford 5-amino-3,4-dimethylthieno­[2,3-*c*]­pyridazines **10** and **11** (Scheme 1). Alternatively, intermediate **28** can be converted to the carboxylate salt under basic conditions and, following a HATU amide coupling with NH_4_Cl, provides intermediate **10** (Scheme 2). Treatment of **10** with trimethylorthoformate under heat afforded pyrimidone intermediate **12** (Scheme 1). Treatment of **12** with POCl_3_ gave chloride **14,** which readily underwent nucleophilic aromatic substitution with a variety of primary and secondary amines to yield desired analogs **15**. For this exercise, as we were generating a novel tricyclic core that had unknown consequences on hM_4_ potency, we chose to evaluate a variety of known amines that provided potent analogs from our previous work.^14–17,23,24^ Further derivatization of **15′** with treatment of a Grignard reagent afforded analogs **19**. Alternatively, when **15′** was a methyl ester, the ester was saponified to the carboxylic acid and, following HATU amide coupling reactions, analogs **20** were afforded. Moreover, chloride **14** was subjected to nucleophilic aromatic substitution with a variety of alcohols to yield desired analogs **16**.

**Scheme 1:**
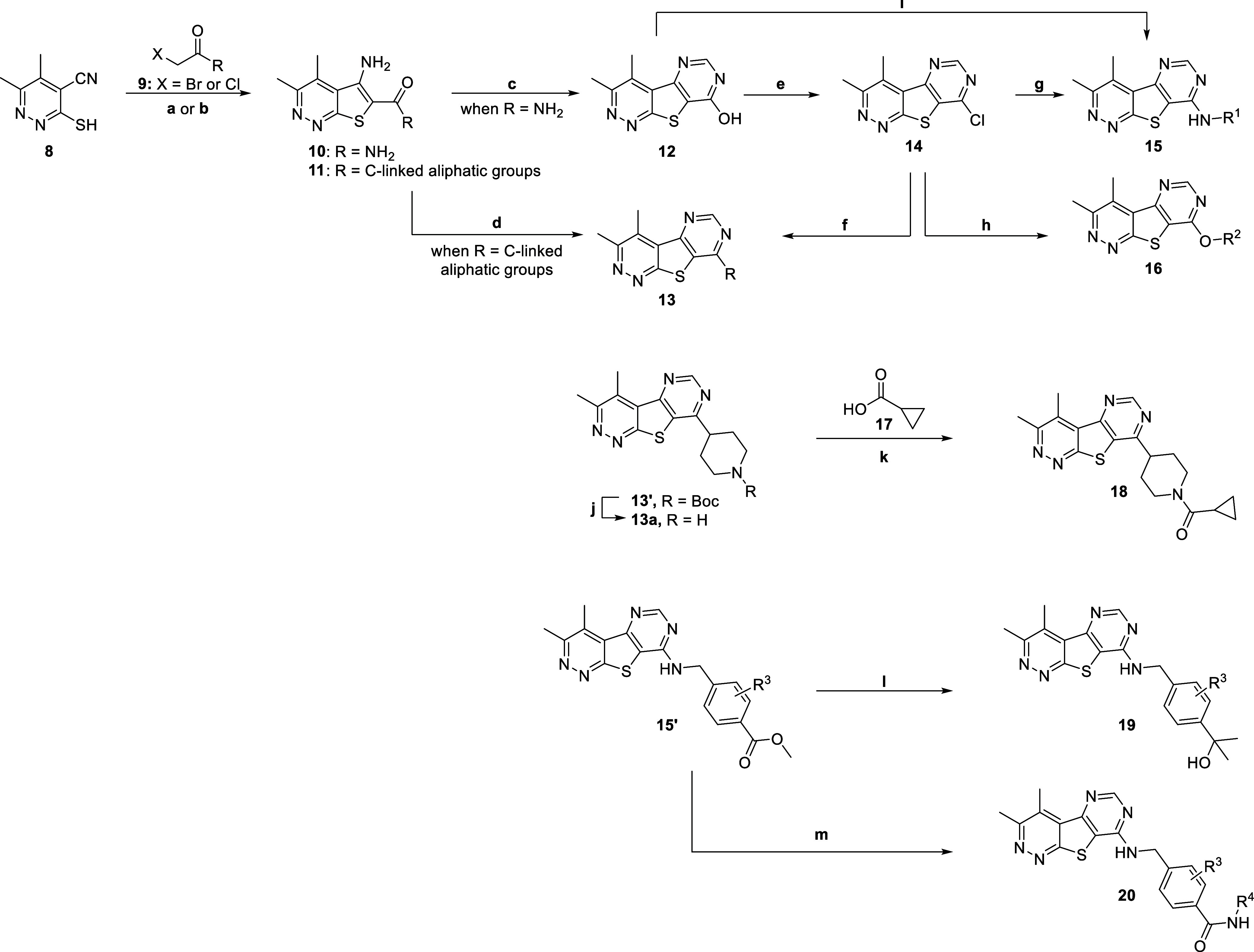
Synthesis of M_4_ Analogs **13**, **16**, **18**, **19,** and **20** ^a^

a Reagents and conditions: (a) 2-chloroacetamide (**9**), Na_2_CO_3_, NMP, 100 °C, 18 h, 81%; (b) **9** (R is C-linked aliphatic groups), KOH, IPA, 105 °C; (c) CH­(OEt)_3_, 150 °C, 12 h, 94%; (d) (i) HCONH_2_, 150 °C; (ii) AcOH·HN=CHNH_2_, 150 °C, 1–13%; (e) POCl_3_, DIEA, DMA, 105 °C, 7 h, 72%; (f) RMgX, Fe­(acac)_3_, THF/NMP, 20–44%; (g) R^1^NH_2_, DIEA, NMP, microwave irradiated at 120 –180 °C, 4–79%; (h) R^2^OH, NaH, THF, 0 °C, 8–39%; (i) R^1^NH_2_, BOP, DBU, NMP, 4–19%; (j) HCl, 1,4-dioxane/H_2_O (1:1), 50 °C, 92%; (k) **17**, HATU, DIEA, DMF, 25%; (l) MeMgBr, THF, 0 °C, 27–68%; (m) (i) LiOH, THF/H_2_O, 50 °C; (ii) R^4^NH_2_, HATU, DIEA, NMP, 6–31%.

**Scheme 2:**
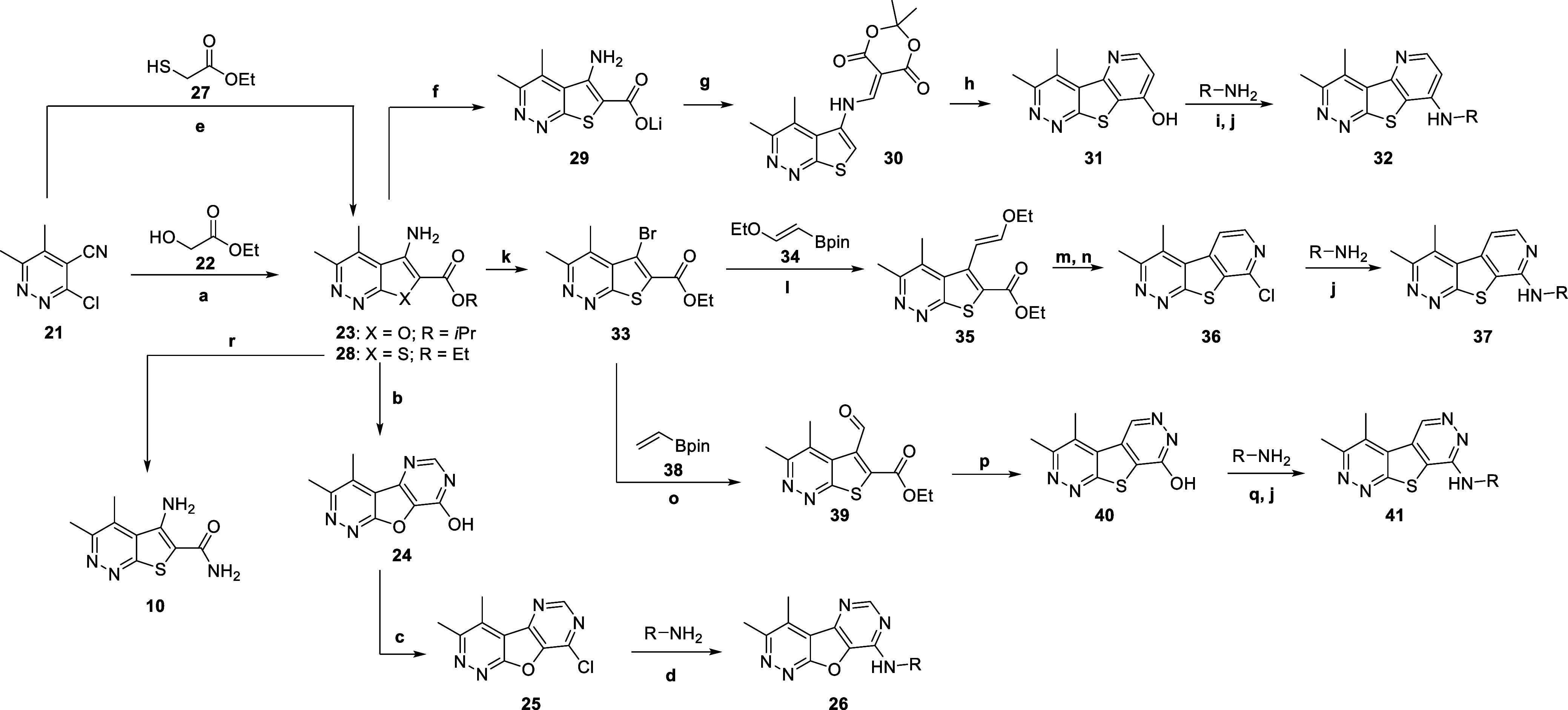
Synthesis of M_4_ Analogs **26**, **33**, **38**, and **41** ^a^

a Reagents and conditions: (a) **22**, K_2_CO_3_, IPA, microwave-irradiated at 150 °C, 0.5 h, 47%; (b) (i) HCONH_2_, 150 °C; (ii) AcOH HN=CHNH_2_, 150 °C, 1.5 h, 82%; (c) POCl_3_, PCl_5_, 120 °C, 24 h, 99%; (d) amine, DIEA, NMP, 60 °C, 0.5 h, 52–54%; (e) (i) **27**, K_2_CO_3_, ^*t*^BuOH, 12 h; (ii) EtOH, 45 °C, 3 h, 95%; (f) LiOH, MeOH/THF, 50 °C, 98%; (g) (i) H_2_O, microwave-irradiated at 160 °C, 1 h; (ii) Meldrum’s acid, HC­(OMe)_3_, 90 °C, 1 h, 22%; (h) Ph_2_O, 230 °C, 40 min, 40%; (i) POCl_3_, 90 °C, 0.5 h, 93%; (j) amine, DIEA, NMP, microwave-irradiated at 120–200 °C, 0.5 h, 4–64%; (k) CuBr_2_, ^*t*^BuONO, ACN, 0.5 h, 79%; (l) **34**, Pd­(dppf)­Cl_2_, Cs_2_CO_3_, 1,4-dioxanes/H_2_O (5:1), 85 °C, 72 h, 98%; (m) (i) TFA, microwave-irradiated at 120 °C, 1 h, 51%; (ii) NH_4_OH, 100 °C, 1.5 h, 93%; (n) POCl_3_, DCE, microwave-irradiated at 150 °C, 0.5 h, 72%; (o) (i) **38**, Pd­(dppf)­Cl_2_, Cs_2_CO_3_, THF/H_2_O (10:1), microwave-irradiated at 130 °C, 0.5 h, 70%; (ii) O_3_, DMS, DCM, −78 °C, 0.5 h, 52%; (p) H_2_N–NH_2_, EtOH/IPA (1:1), 80 °C, 1 h, 98%; (q) POCl_3_, TEA, DCE, 0 °C then 105 °C, 5 h, 47%, (r) (i) LiOH, MeOH/THF (1:3.3); (ii) HATU, DIEA, NH_4_Cl, NMP, 18 h, 78%—2 steps.

Select analogs **15**, **16**, **19**, and **20** were tested for activity in Chinese Hamster Ovary (CHO) cells expressing human M_4_ (hM_4_) to determine EC_50_ values, with results highlighted in Table 1. This exercise proved to be very fruitful, as we generated several compounds with hM_4_ PAM functional potencies less than 500 nM. Interestingly, many of these analogs were several fold more potent than their respective 7,9-dimethylpyrido­[3′,2′:4,5]­thieno [3,2-*d*]­pyrimidine core counterparts (Figure 1, core **1**) previously discussed.^23^ Most notable was the increase in potency of the benzylamine analogs **15l**–**w** and **19a**–**e**. Previously, similar analogs with benzyl amine tail groups in conjugation with the 7,9-dimethylpyrido­[3′,2′:4,5]­thieno [3,2-*d*]­pyrimidine core (**1**) provided compounds with hM_4_ PAM functional potencies ranging from inactive to low micromolar EC_50_s, suggesting the importance of the pyridazine ring of the tricyclic core structure **5**.

**Table 1:**
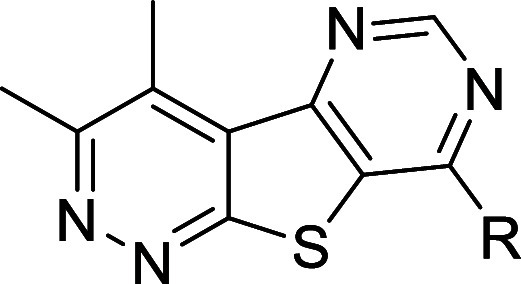

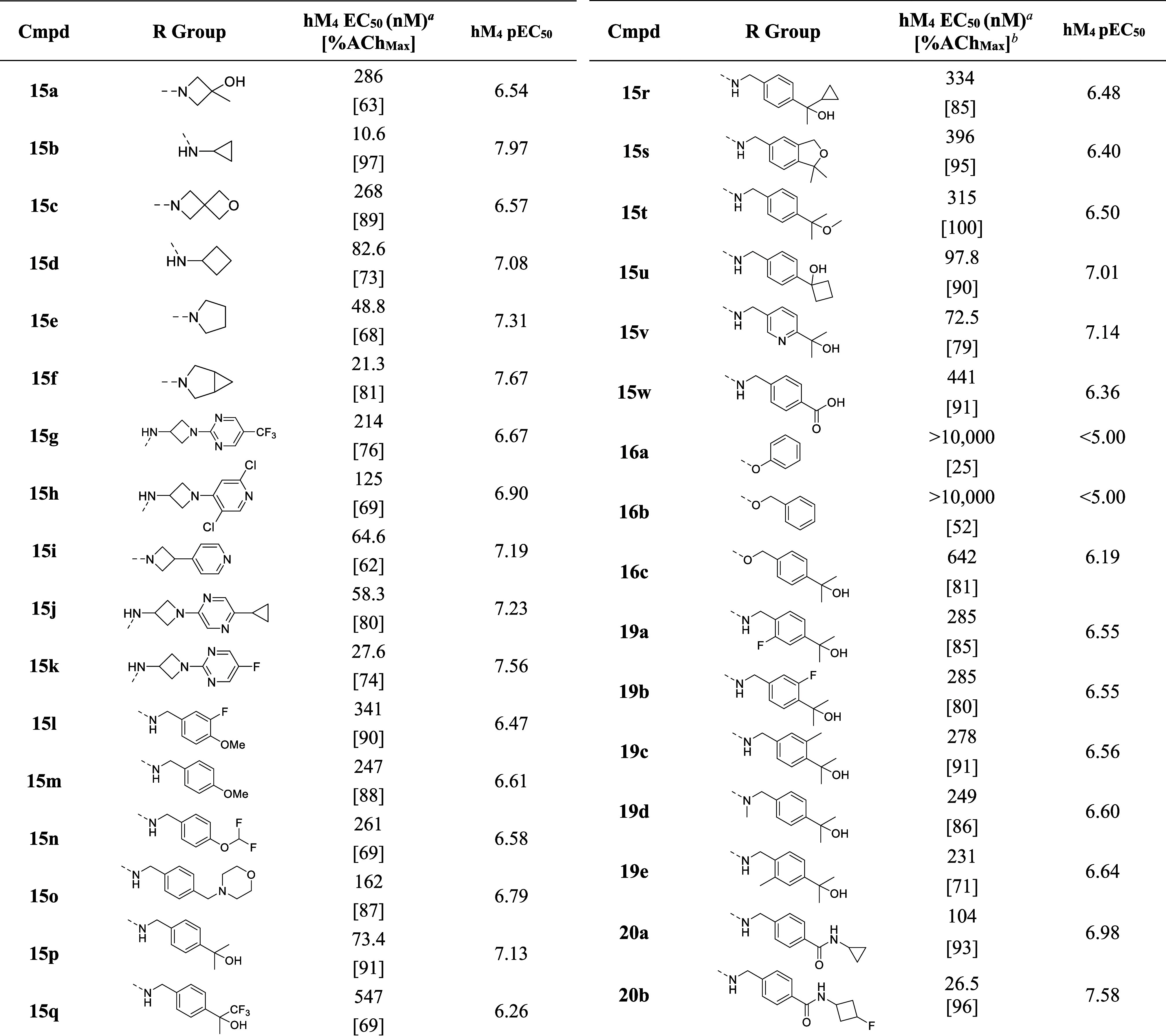
Structures and Activities for Analogs **15**, **16**, **19**, and **20** ^a^

a Calcium mobilization assays with hM_4/Gqi5_-CHO cells performed in the presence of an EC_20_ fixed concentration of acetylcholine, *n* = 1–3 independent experiments in triplicate.

b Data are normalized to 100 μM acetylcholine [% ACh_Max_].

Although small aliphatic amino tail groups (**15a**–**15f**) and amino azetidine tail groups (**15g-h** and **15k-l**) provided some highly potent analogs in the past, they historically suffer from poor DMPK profiles and/or chemical stability issues.^39^ Thus, once we identified a novel tricyclic core, we quickly pivoted our attention to exploring alternate amines with the goal of improving DMPK. To this end, we focused our attention on benzylamine tail groups. While *p*-methoxybenzyl amine derivative **15m** resulted in an hM_4_ EC_50_ of 247 nM, addition of an electronegative fluorine ortho to the methoxy group (**15l**, hM_4_ EC_50_ = 341 nM) resulted in a slight loss of hM_4_ PAM activity. Conversely, addition of fluorine to the methoxy group to give difluoromethoxy analog **15n** (hM_4_ EC_50_ = 261 nM) had no deleterious effect on potency. Interestingly, the activity of morpholine analog **15o** (hM_4_ EC_50_ = 162 nM) indicated that larger substituents on the benzyl ring would be well tolerated. To increase the aqueous solubility of our analogs, we synthesized tertiary alcohol **15p** (hM_4_ EC_50_ = 73.4 nM), which afforded one of the most potent compounds within our initial benzylamine series.

Further derivatization of the tertiary alcohol benzylamine generated compounds **15q**–**15v** and **19a**–**19e**. Substituting one methyl group of tertiary alcohol **15p** with a trifluoromethyl group (**15q**, hM_4_ EC_50_ = 547 nM) resulted in a 7.5-fold loss of activity. Additionally, increasing steric bulk around the tertiary alcohol (**15r**, hM_4_ EC_50_ = 334 nM; **15u**, hM_4_ EC_50_ = 97.8 nM), as well as restricting rotation (**15s**: hM_4_ EC_50_ = 396 nM), all resulted in a loss of hM_4_ activity to varying degrees. Converting the alcohol of **15p** into a methyl ether (**15t**, hM_4_ EC_50_ = 315 nM) also resulted in a 4-fold decrease in potency. Likewise, *N*-alkylation of the benzyl amine to give tertiary amine **19d** (hM_4_ EC_50_ = 249 nM) resulted in a 3.4-fold loss of hM_4_ activity. Similarly, fluorine or methyl substitutions on the benzene ring (**19a**: hM_4_ EC_50_ = 285 nM; **19b**: hM_4_ EC_50_ = 285 nM; **19c**: hM_4_ EC_50_ = 278 nM; **19e**: hM_4_ EC_50_ = 231 nM) caused a 3 to 4-fold loss of hM_4_ activity. Interestingly, substituting the benzene ring with a pyridine (**15v**: hM_4_ EC_50_ = 72.5 nM) afforded an analog comparable in potency to **15p** and was 4-fold more potent than the corresponding fluorobenzene analog **19b**.

To determine if amides were a suitable replacement for the tertiary alcohol of **15p**, we synthesized analogs **20**. To accomplish this, we began with methyl ester **15,** which was then saponified to the carboxylic acid in the presence of LiOH. Following standard HATU coupling conditions, the desired analogs **20** were afforded. Interestingly, replacing the tertiary alcohol with various small amide groups (**20a**: hM_4_ EC_50_ = 104 nM; **20b**: hM_4_ EC_50_ = 26.5 nM) resulted in highly potent analogs. Conversely, exchanging the amine linker (**15**) for an ether linkage (**16**) resulted in a loss of the hM_4_ functional potency. Analogs **16** could be easily accessed by treating various alcohols with NaH followed by nucleophilic aromatic substitution with chloride **14**. Directly comparing amine-linked analog **15p** (hM_4_ EC_50_ = 73.4 nM) and ether-linked analog **16c** (hM_4_ EC_50_ = 642 nM) demonstrated a nearly 9-fold loss of hM_4_ potency, highlighting the importance of the amine linker in regard to benzyl tail groups.

We next examined replacing the benzyl amine tail group with small, carbon-linked aliphatic groups **13** and **18,** which were synthesized in accordance with Scheme 1. Briefly, intermediates **11** underwent a condensation with formamide, affording final compounds **13**. The piperidine intermediate **13a** could be further transformed via HATU amide coupling with carboxylic acid **17** to yield analog **18**. Select analogs **13** and **18** were screened against hM_4_ to determine potency, with results highlighted in Table 2. In comparison to the previously described tricyclic core **1** analogs, analogs showcased in Table 2 containing the tricyclic core **5** displayed greater hM_4_ PAM activity.^23^ In fact, this exercise provided several analogs with hM_4_ functional potencies ≤300 nM. Similar to our previous studies, the potency of the piperidine analog **13a** (hM_4_ EC_50_ = 4.27 μM) could greatly be improved (∼10.5-fold) via conversion into the amide analog **18** (hM_4_ EC_50_ = 408 nM). Unlike our previous study, the cyclohexyl analog **13g** (hM_4_ EC_50_ = 51.6 nM) was 2.3-fold more potent than the tetrahydropyran analog **13f** (hM_4_ EC_50_ = 120 nM). This endeavor revealed that the benzyl amine tail group was not essential for hM_4_ activity.

**Table 2:**
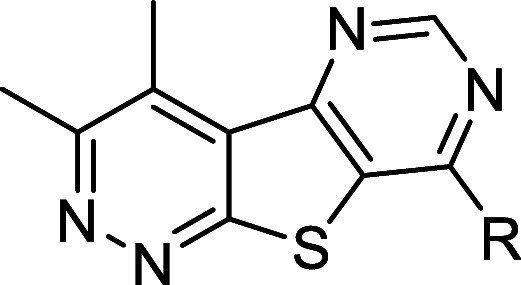

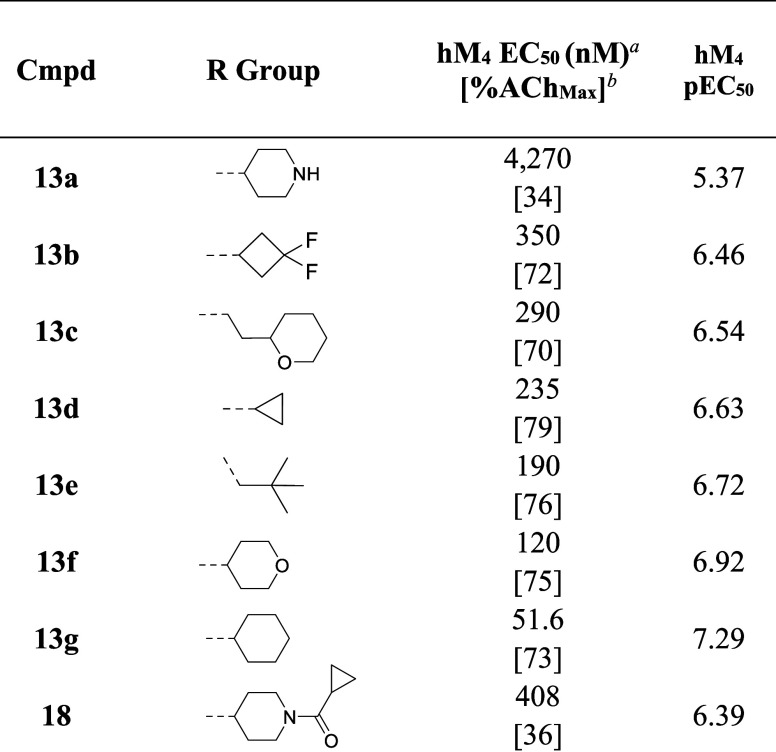
Structures and Activities for Analogs **13** and **18** ^a^

a Calcium mobilization assays with hM_4/Gqi5_-CHO cells performed in the presence of an EC_20_ fixed concentration of acetylcholine, *n* = 1–3 independent experiments in triplicate.

b Data are normalized to 100 μM acetylcholine [% ACh_Max_].

To analyze the importance of the thiophene of the tricyclic core, we synthesized the corresponding furan-containing tricycle (**26**) according to Scheme 2. Briefly, the reaction of chloride **21** with alcohol **22** in the presence of potassium carbonate and IPA afforded 3,4-dimethylfuro­[2,3-*c*]­pyridazine **23** after microwave irradiation. Intermediate **23** was then condensed with formamide, followed by formamidine acetate salt to afford pyrimidone **24,** which was then treated with POCl_3_/PCl_5_ to give chloride **25**. Chloride **25** readily underwent nucleophilic aromatic substitution to yield desired analogs **26**. Select analogs **26a**–**d** were screened against hM_4_ to determine functional potencies, with results highlighted in Table 3. While these analogs still displayed functional hM_4_ activity, the potency of these analogs was diminished. Most notably, analog **26c** was nearly 25-fold less potent when compared to its thiophene tricyclic counterpart, **15p**. Similarly, analog **26a** resulted in a 20.5-fold loss of functional hM_4_ activity as compared to **15d**.

**Table 3:**
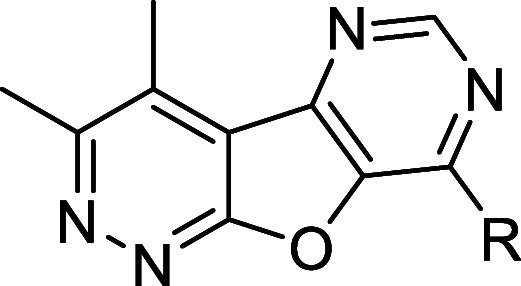

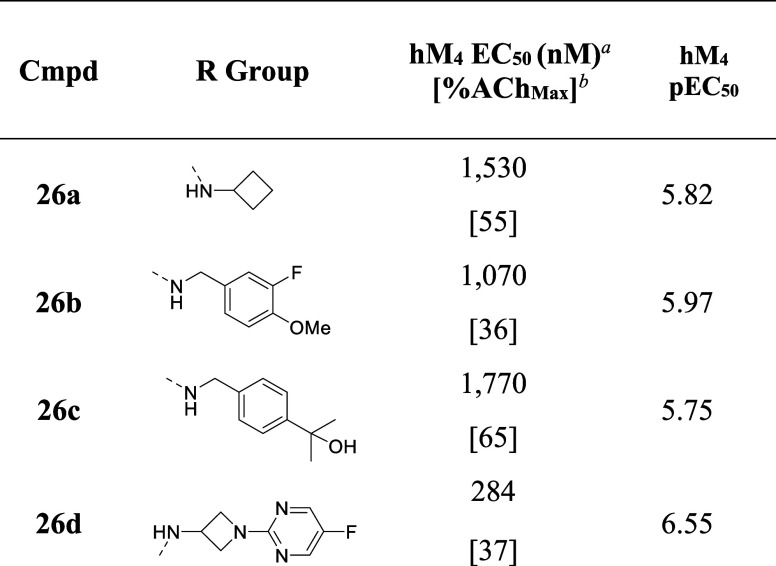
Structures and Activities for Analog **26** ^a^

a Calcium mobilization assays with hM_4/Gqi5_-CHO cells performed in the presence of an EC_20_ fixed concentration of acetylcholine, *n* = 1–3 independent experiments in triplicate.

b Data are normalized to 100 μM acetylcholine [% ACh_Max_].

Moving forward, we explored modifications to the pyrimidine ring of the tricyclic core to assess the impact each nitrogen contributes to hM_4_ potency. To do so, we began with the synthesis of analogs **32** (Scheme 2). Intermediate **28** was prepared in a similar manner, as previously described for intermediate **23**. Intermediate **28** was first saponified to the corresponding lithium salt **29,** which then underwent decarboxylation under microwave irradiation. Further reaction with Meldrum’s salt in the presence of trimethyl orthoformate yielded intermediate **30**. Heating intermediate **30** in diphenyl ether at a high temperature resulted in pyridinone **31**, which could be readily chlorinated in the presence of POCl_3_. Subsequent aromatic nucleophilic substitution yielded desired analogs **32a**-**b**. To prepare analogs **37**, intermediate **28** was subjected to a copper-catalyzed Sandmeyer reaction to generate bromide **33,** which could readily undergo a Suzuki–Miyaura coupling with pinacol borane **34** to afford carboxylate **35**. A two-step process was followed, in which intermediate **35** was first subjected to TFA and heat to yield 3,4-dimethyl-8*H*-pyrano­[4′,3′:4,5]­thieno­[2,3-*c*]­pyridazin-8-one and then further transformed in the presence of NH_4_OH and heat to generate 3,4-dimethylpyrido­[4′,3′:4,5]­thieno­[2,3-*c*]­pyridazin-8-ol, which was then converted to chloride **36** upon treatment with POCl_3_. Subsequent nucleophilic aromatic substitution with various amines gave analogs **37**. Finally, analogs **41** were prepared by subjecting intermediate **33** to standard Suzuki–Miyaura coupling conditions with pinacol borane **38**. Subsequent treatment with hydrazine and heat led to the pyridazinone intermediate **40**. After treatment with POCl_3_ to give the chloride intermediate, which readily underwent aromatic nucleophilic substitution, the desired analogs **41** were obtained. Analogs **32**, **37**, and **41** were screened against hM_4_ to determine functional potencies, with results highlighted in Table 4.

**Table 4:**
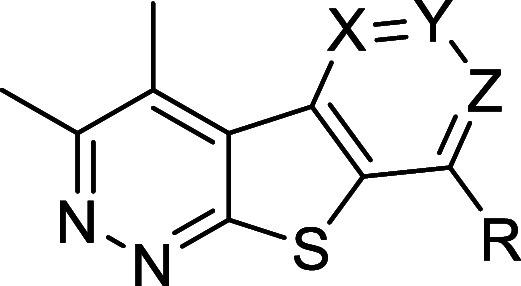

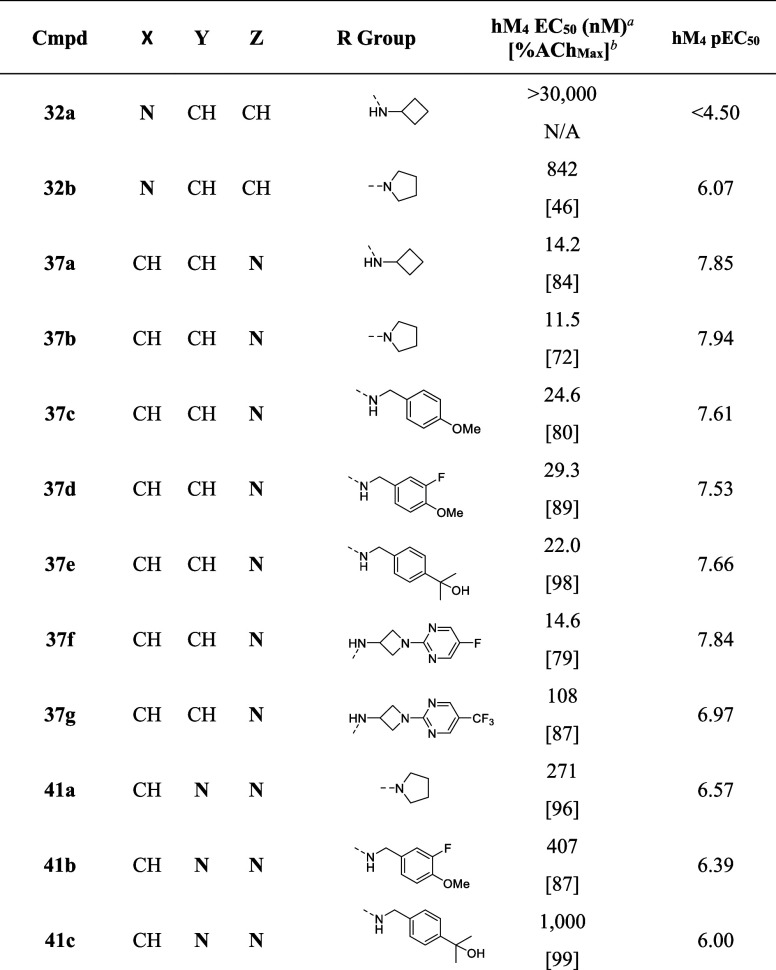
Structures and Activities for Analogs **32**, **37**, and **41** ^a^

a Calcium mobilization assays with hM_4/Gqi5_-CHO cells performed in the presence of an EC_20_ fixed concentration of acetylcholine, *n* = 1–3 independent experiments in triplicate.

b Data are normalized to 100 μM acetylcholine [% ACh_Max_].

It became quite clear that nitrogen at the 7-position (“Z”) of the pyrimidine ring was essential for low nanomolar potency. Comparing analog **32a** (hM_4_ EC_50_ > 30 μM) and **32b** (hM_4_ EC_50_ = 842 nM) with analogs **37a** (hM_4_ EC_50_ = 14.2 nM) and **37b** (hM_4_ EC_50_ = 11.5 nM), respectively, highlights the importance of this nitrogen. Alternatively, it was noted that nitrogen at the 5-position (“X”) of the pyrimidine ring was not required for activity. In fact, analogs lacking this nitrogen were often more potent than the pyrimidine predecessor. For example, the pyrimidine analog **15m** (hM_4_ EC_50_ = 247 nM) was 10-fold less potent than the corresponding pyridine analog **37c** (hM_4_ EC_50_ = 24.6 nM). Similarly, pyrimidine analog **15p** (hM_4_ EC_50_ = 73.4 nM) was 3-fold less potent than the analogous pyridine analog **37e** (hM_4_ EC_50_ = 22.0 nM). Moreover, the pyridazine ring (“Y” and “Z” are nitrogen) resulted in a loss of potency when compared to the pyrimidine predecessor. For example, the pyrimidine analog **15e** (hM_4_ EC_50_ = 48.8 nM) was 5.5-fold more potent than the corresponding pyridazine **41a** (hM_4_ EC_50_ = 271 nM). Furthermore, the pyrimidine analog **15p** was 13-fold more potent than the pyridazine analog **41c** (hM_4_ EC_50_ = 1.0 μM). These results further illustrate the significance of nitrogen at the 7-position.

We next turned our attention to evaluating various substitutions at the 3- or 6-position of the 3,4-dimethylpyrimido­[4′,5′:4,5]-thieno­[2,3-*c*]­pyridazine core (Scheme 3). Synthesis of the 6-methyl analog **45** began with intermediate **29,** which was microwave irradiated in the presence of acetic anhydride to yield the oxazinone **42**. Intermediate **42** was then converted into pyrimidinone **43** in the presence of ammonium carbonate and acetic acid with microwave irradiation. Following POCl_3_ treatment to afford chloride **44**, nucleophilic substitution afforded final compounds **45**. To synthesize the 3-chloro or 3-methoxy analogs **49** and **50**, intermediate **47** was first generated in a similar manner as previously described for intermediate **29**. HATU coupling of intermediate **47** with ammonium chloride gave the primary amide which after treatment with trimethylorthoformate under heat afforded the cyclized pyrimidone intermediate **48**. Following POCl_3_ treatment to afford the chloropyrimidine, nucleophilic substitution afforded final compounds **49**. These analogs could further be treated with sodium methoxide to yield final compounds **50**. To synthesize the 3-difluoromethyl analogs **55**, starting pyridazine **46** was first converted to the *N*-oxide with *m*CPBA and then treated with acetic anhydride and heated to afford intermediate **51**. Cyclization with ethyl thioglycolate followed by deprotection of the acetyl group and subsequent oxidation with MnO_2_ afforded aldehyde **52**. Following treatment with DAST to afford the difluoromethyl intermediate **53**, final compounds **55** were synthesized in a similar manner as described for **49** and **50**. Analog **58** was synthesized by first subjecting intermediate **14** to NBS to afford bromide **56,** which readily underwent displacement in the presence of potassium acetate. Following nucleophilic aromatic substitution to give intermediate **57**, deprotection of the acetyl group afforded the final compound **58**. Final compound **59** was generated as an unexpected side product after treating **15′** with MeMgBr in an attempt to synthesize **19a**. Functional hM_4_ potencies for analogs **45**, **49**, **50**, **55**, and **57–59** were determined, and the results are highlighted in Table 5.

**Scheme 3:**
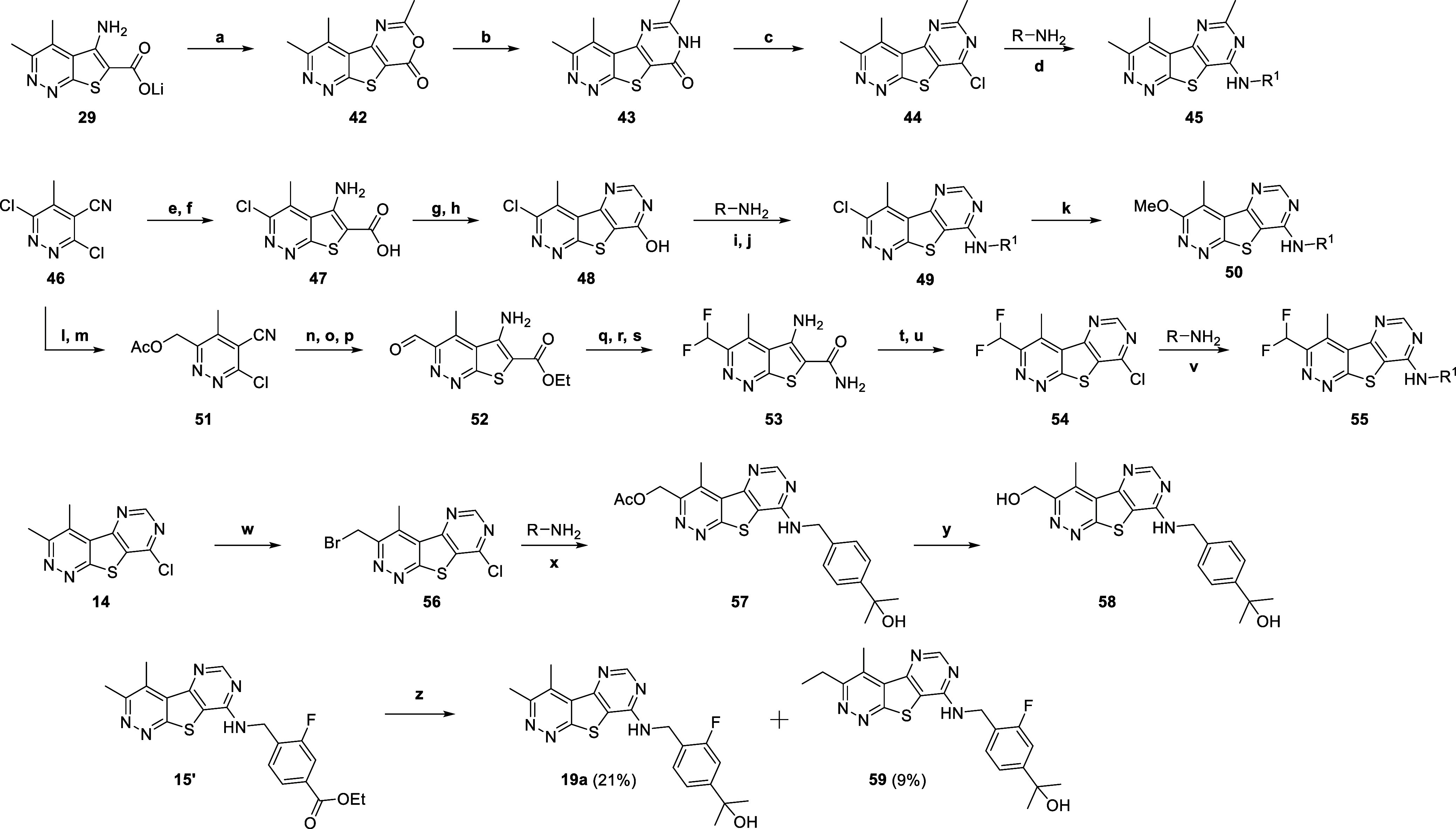
Synthesis of Analogs **45**, **50**, **54**, **55**, and **57–59** ^a^

a Reagents and conditions: (a) Ac_2_O, microwave irradiated at 180 °C, 15 min, 72%; (b) (i) MeCO_2_NH_4_, AcOH, microwave irradiated at 150 °C, 15 min, 60%; (c) POCl_3_, microwave irradiated at 150 °C, 15 min, 80%; (d) amine, DIEA, NMP, 75 °C, 3 h, 13–15%; (e) methyl thioglycolate, Na_2_CO_3_, DMF, 0 °C, then 23 °C, 0.5 h, 85%; (f) 1 M LiOH, THF, 70 °C, 18 h, 92%; (g) NH_4_Cl, HATU, DIEA, NMP, 18 h, 97%; (h) CH­(OEt)_3_, 155 °C, 3 h, 79%; (i) POCl_3_, TEA, 90 °C, 1 h, 85%; (j) amine, TEA, NMP, microwave irradiated at 120 °C, 10 min, 16–37%; (k) NaOMe, MeOH, microwave irradiated at 130 °C, 15 min, 9–36%; (l) *m*CPBA, DCM, 18 h, 81%; (m) Ac_2_O, 125 °C, 16 h, 33%; (n) ethyl thioglycolate, Na_2_CO_3_, DMF, 43 °C, 3 h, then 90 °C, 1 h, 94%; (o) K_2_CO_3_, EtOH, 2 h, 99%; (p) MnO_2_, DCE/THF (1:1), 80 °C, 75%; (q) DAST, DCE, 0 °C, then 23 °C for 1 h, 44%; (r) 1 M LiOH, MeOH/THF (1:4), 4 h, 88%; (s) NH_4_Cl, HATU, DIEA, NMP, 2 h, 76%; (t) CH­(OEt)_3_, 150 °C, 3 h, 99%; (u) POCl_3_, TEA, DCE, 100 °C, 3 h, 20%; (v) amine, DIEA, NMP, microwave irradiated at 120 °C, 15 min, 26–63%; (w) NBS, (BzO)_2_, CCl_4_, 83 °C, 2 h, 76%, (x) (i) KOAc, DMF, 15 min; (ii) amine, DIEA, 30 min, 93%; (y) LAH, THF, −78 °C, 1 h, 13%; (z) MeMgBr, THF, 2 h.

**Table 5:**
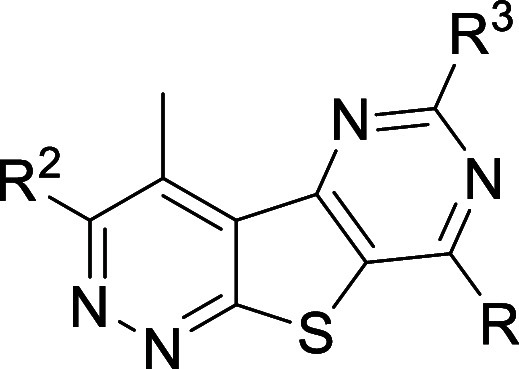

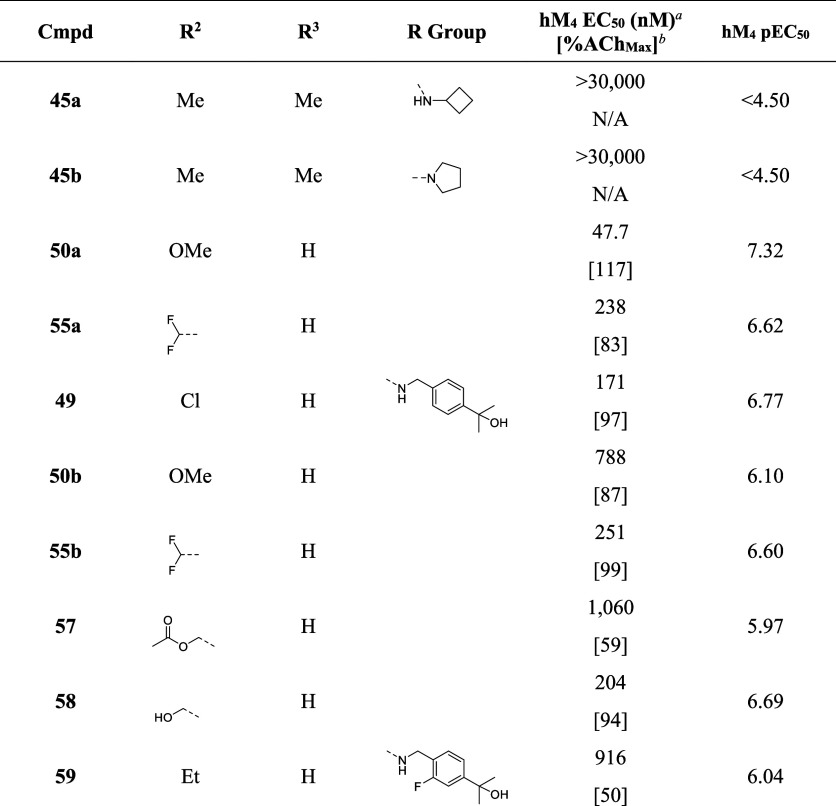
Structures and Activities for Analogs **45**, **49**, **50**, **55**, and **57–59** ^a^

a Calcium mobilization assays with hM_4/Gqi5_-CHO cells performed in the presence of an EC_20_ fixed concentration of acetylcholine, *n* = 1–3 independent experiments in triplicate.

b Data are normalized to 100 μM acetylcholine [% ACh_Max_].

Methylation of the pyrimidine ring at the 6-position was detrimental and resulted in a drastic loss of activity (**45a** and **45b**). While substitutions at the 3-position of the pyridazine ring were tolerated, these modifications also resulted in a loss of potency. When compared to **15p** (hM_4_ EC_50_ = 73.4 nM), the chlorine analog **49** (hM_4_ EC_50_ = 171 nM) resulted in a two-fold loss of potency, while the methoxy analog **50b** (hM_4_ EC_50_ = 788 nM) displayed a more dramatic loss in potency (∼11-fold). Interestingly, substituting the methyl group for a difluoromethyl group (**55b**; hM_4_ EC_50_ = 251 nM) led to only a three-fold loss of potency. Moreover, going from a methyl substituent (**19a**, hM_4_ EC_50_ = 285 nM) to an ethyl group (**59**; hM_4_ EC_50_ = 916 nM) also led to a loss of potency (3.4-fold). These data, taken together, demonstrate the tight structure activity relationship around the tricyclic ring.

### In Vitro DMPK and Molecular Pharmacology Profiling

Of these compounds, **15e**, **15k**, **15p**, **20b**, **13g**, **37d**, and **37e** were selected based on potency (hM_4_ EC_50_ < 75 nM) and chemical diversity to advance into a battery of molecular pharmacology and in vitro DMPK assays to assess their suitability to advance further down the testing cascade toward a putative preclinical candidate (Tables 6 and 7). In relation to physicochemical properties, these analogs all possessed molecular weights less than 450 Da with **13g**, **15p**, and **20b** having the most attractive CNS *x* log *P* values (2.8–3.4).^40,41^ Analogs **13g**, **15e**, and **37d** displayed high human and rat predicted hepatic clearance based on microsomal CL_int_ data (human CL_hep_ > 15 mL/min/kg; rat CL_hep_ > 56 mL/min/kg); however, all other analogs tested displayed moderate human and rat predicted hepatic clearance based on microsomal CL_int_ data (human CL_hep_ of 8.2–14 mL/min/kg, rat CL_hep_ of 32–53 mL/min/kg). While **13g**, **15p**, **20b**, **37d**, and **37e** were highly bound to human plasma protein (*f*_u,plasma_ 0.005–0.009), only **20b** and **37d** were also highly bound to rat plasma protein (*f*_u,plasma_ 0.006–0.008). By contrast, analogs **13g**, **15e**, **15k**, **15p**, and **37e** exhibited reduced rat plasma protein binding profiles (rat *f*_u,plasma_ 0.012–0.107). Interestingly, compounds **15e** and **15k** displayed the best overall protein binding values (rat *f*_u,plasma_ 0.029–0.107; rat *f*_u,brain_ 0.031–0.065; human *f*_u,plasma_ 0.060–0.067); however, these analogs were not progressed forward due to either high predicted hepatic clearance or poor brain distribution. These data led us to select compound **15p** (**VU6008055**) for further profiling.

**Table 6:** In Vitro DMPK and Rat PBL Data for Select Analogs

	**15e**	**15k**	**15p**	**20b**	**13g**	**37d**	**37e**
property	**VU6006867**	**VU6007458**	**VU6008055**	**VU6009990**	**VU6009096**	**VU6009585**	**VU6009795**
*M*_W_	285.37	382.42	379.48	436.51	298.41	368.43	378.49
*x* log *P*	2.2	1.29	3.4	3.18	3.4	3.66	3.86
TPSA	54.8	92.6	83.8	92.7	51.6	59.9	70.9
hM_4_ EC_50_ (nM)	48.8	27.6	73.4	26.5	51.6	29.3	22.0
In Vitro PK Parameters							
CL_int_ (mL/min/kg), rat	471	60	89	66	801	699	224
CL_hep_ (mL/min/kg), rat	61	32	39	34	64	64	53
CL_int_ (mL/min/kg), human	423	33	17	42	188	156	40
CL_hep_ (mL/min/kg), human	20	13	9	14	19	19	14
rat *f*_u,plasma_ ^a^	0.107	0.029	0.020	0.008	0.040	0.006	0.012
human *f*_u,plasma_ ^a^	0.06	0.067	0.007	0.009	0.008	0.006	0.008
rat *f*_u,brain_ ^a^	0.065	0.031	0.015	0.019	0.004	0.007	0.022
Brain Distribution (0.25 h) (SD Rat; 0.2 mg/kg IV)							
*K*_p,brain:plasma_ ^b^	3.19	0.37	0.55	0.02	4.02	BLQ	0.4
*K*_p,uu,brain:plasma_ ^c^	1.94	0.4	0.41	0.04	0.4	BLQ	0.73

a *f*_u_ = Fraction unbound; equilibrium dialysis assay; brain = rat brain homogenates.

b *K*_p_ = total brain to total plasma ratio.

c *K*_p,uu_ = unbound brain (brain *f*_u_ × total brain) to unbound plasma (plasma *f*_u_ × total plasma) ratio.

**Table 7:** **VU6008055 (15p)** Muscarinic EC_50_s and Selectivity Profiles of Various Species^b^

Muscarinic Selectivity^a^					
EC_50_ (nM) [% AchMax] pEC_50_					
	human	rat	dog	cyno	minipig
M_4_	73.4 [91] 7.13	19.5 [81] 7.71	36.3 [72] 7.44	63.5 [89] 7.20	34.3 [72] 7.46
M_2_	2820 [49] 5.5	2410 [53] 5.62	2790 [38] 5.55	3000 [34] 5.52	3000 [43] 5.52
M_1_	inactive	inactive	n.d.	n.d.	n.d.
M_3_	inactive	inactive	inactive	n.d.	n.d.
M_5_	inactive	inactive	n.d.	n.d.	n.d.

a Calcium mobilization assay; values are an *n* ≥ 1 independent experiments in triplicate (n.d. = not determined).

b Data are normalized to 100 μM acetylcholine [% ACh_Max_].

When evaluated utilizing a calcium mobilization assay, **VU6008055** (**15p**) displayed high subtype selectivity across the human mAChRs (M_1_, M_3_, and M_5_ = inactive; M_2_ = 41-fold selectivity) in addition to exhibiting no appreciable species differences in M_4_ activity (<4-fold) (Figure 3, Table 7).^42,43^ In a competition binding experiment, **VU6008055** completely displaced the binding of the M_4_ subtype-selective radioligand [^3^H]­MK-6884 at both human and rat M_4_ (Figure 3B). Moreover, **VU6008055** demonstrated acceptable CYP_450_ profiles against CYP1A2, CYP2B6, CYP2C8, CYP2C9, CYP2C19, CYP2D6, and CYP3A4 (IC_50_s > 15 μM).^44^ **VU6008055** did not exhibit time-dependent inhibition of any of the CYPs tested. Next, we assessed in vitro brain penetration potential utilizing MDCKII-MDR1 transfected cells.^44^ **VU6008055** exhibited an efflux ratio (ER) of 0.83 and a *P*_app_ (A-B) of 6.4 × 10^–6^. To further evaluate the potential involvement of P-glycoprotein 1 (P-gp) in the transport of **VU6008055**, the same experiment was performed in the presence of a known P-gp inhibitor (elacridar), giving an ER = 1.1 and a *P*_app_ (A-B) of 8.0 × 10^–6^. These data indicated that **VU6008055** is not a substrate of the efflux transporter P-gp and is classified as having moderate brain permeability, which was acceptable to move forward. We next assessed the in vitro permeability and active transport in MDCKII-BCRP transfected cells, and similar to the MDCKII-MDR1 assay results, **VU6008055** exhibited moderate permeability; however, the ratio between permeabilities with (ER = 1.3) and without (ER = 7.5) a BCRP inhibitor suggests that **VU6008055** is a substrate of the human efflux transporter BCRP.

**Figure 3:**
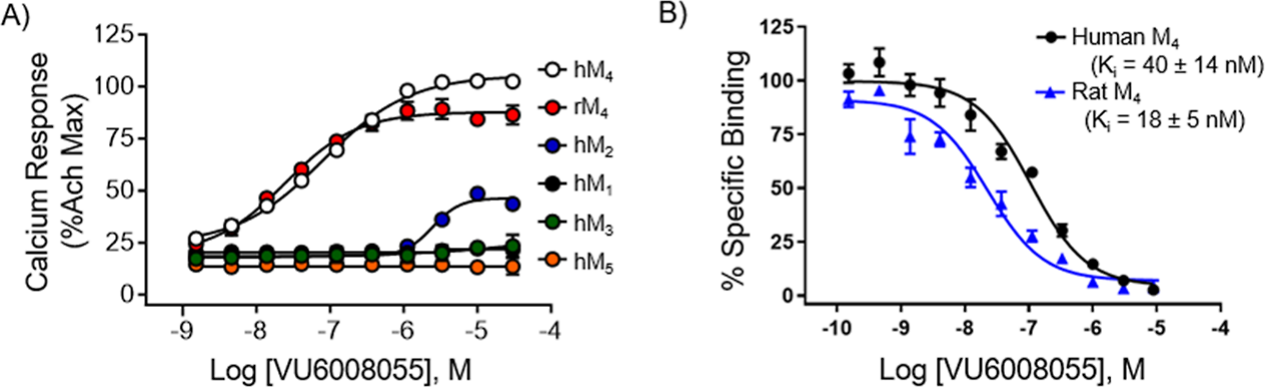
Dose response curves of **VU6008055** showing (A) selectivity with human M_1_–M_5_ and rat M_4_ and (B) competition binding against [^3^H]­MK-6884 at human (*n* = 3) and rat (*n* = 2) M_4_.

We next turned our attention to investigating the off-target and safety and toxicity profiles of **VU6008055**. In a Lead Profiling Screen employing radioligand displacement to evaluate ancillary pharmacology, a panel of 68 potential off-target GPCRs, ion channels and transporters were screened. The study revealed no off-target activity of **VU6008055** (≤50% inhibition at 10 μM across all off-targets screened).^44^ Additionally, the compound was negative in a 4-strain Ames assay (with and without S9). A glutathione (GSH) trapping study in human liver microsomes to access potential reactive intermediates/metabolites was also negative. Finally, an electrophysiology panel of cardiac ion channels was clean (<13% at 10 μM).^44^ Thus, **VU6008055** possessed acceptable overall safety/toxicity profiles and was advanced toward preclinical candidate selection.

### In Vivo DMPK and Behavioral Pharmacology

**VU6008055** was next evaluated in our standard rat IV plasma:brain level (PBL) cassette paradigm (0.2 mg/kg compound, 1 mg/kg total dose, 9.9% EtOH:39.6 PEG400:50.5% DMSO) sampled at a set 15 min time point to assess CNS penetration. **VU6008055** had a *K*_p_ of 0.55 (plasma, 92.0 ng/mL; brain, 50.4 ng/g) and a *K*_p,uu_ of 0.41. These data were in alignment with the previously discussed human predicted CNS penetration from the MDCKII-MDR1 P-gp in vitro assay and supported continued advancement.

As we planned to evaluate our compound in rodent pharmacodynamic (PD) assays, we next assessed in vivo rat IV/PO PK parameters. As highlighted in Table 8, **VU6008055** displayed high oral bioavailability (≥100% at a 3 mg/kg dose and 89% at a 30 mg/kg dose) and low plasma clearance (4.7 mL/min/kg) in rat. Moreover, the volume of distribution was moderate (rat *V*_ss_ = 0.7 L/kg), indicating wide tissue distribution, and elimination half-life was acceptable (rat *t*_1/2_ = 2.63 h). In a rat single PO dose escalation PK study, **VU6008055** demonstrated an increase in the mean AUC_0–∞_ from 3 to 30 mg/kg. Using a preclinical model of antipsychotic-like activity, **VU6008055** produced a robust dose-dependent blockade of amphetamine-induced hyperlocomotion (AHL) after oral administration following a 30 min pretreatment interval in rats (MED = 0.3 mg/kg, Figure 4). At the end of the study, brain:plasma *K*_p_s and *K*_p,uu_s were determined at all dose groups (*K*_p_s = 0.26–034; *K*_p,uu_s = 0.18–0.23) with mean *C*_brain,unbound_ ranging from 0.71 nM (0.1 mg/kg) to 69 nM (10 mg/kg) (Table 9). These data were in alignment with our PBL cassette data.

**Table 8:** In Vivo Rat IV/PO PK Profile of **VU6008055**

IV PK					
species	dose (mg/kg)	*t*_1/2_ (hr)^e^	MRT (h)^e^	CL_p_ (mL/min/kg)^e^	*V*_ss_ (L/kg)^e^
rat (SD)^a^	1	2.63	2.65	4.7	0.7
dog^b^	1	4.17	5.05	5.0	1.4
monkey^c^	1	4.54	3.16	5.0	0.9
minipig^d^	2	5.50	4.9	7.7	2.2

PO PK					
species	dose (mg/kg)	*T*_max_ (h)	*C*_max_ (ng/mL)	AUC_0–∞_ (h·ng/mL)	% *F*
rat (SD)^f^	3	1.5	1695	14,801	>100
rat (SD)^f^	30	3.0	8985	101,444	89
dog^g^	3	2.0	766	5920	54
monkey^h^	3	2.0	960	4696	44
minipig^i^	5	1.2	600	8000	50

a Male Sprague–Dawley rats (*n* = 3); vehicle = 7% EtOH, 60% PEG 400, 33% Saline (2 mL/kg).

b Male beagle dogs (*n* = 3); vehicle = 6.7% EtOH, 60% PEG400, 33.3% water (1 mL/kg).

c Male cynomolgus monkeys (*n* = 3); vehicle = 6.7% EtOH, 60% PEG400, 33.3% water (1 mL/kg).

d Male Göttingen Minipig (*n* = 3); vehicle = 10% HP-betaCD (2.5 mL/kg).

e *t*_*1/2*_ = Terminal phase plasma half-life; MRT = mean residence time; Cl_p_ = plasma clearance observed; *V*_ss_ = volume of distribution at steady-state.

f Male Sprague–Dawley rats (*n* = 2); vehicle = 10% Tween-80, 90% water (10 mL/kg).

g Male beagle dogs (*n* = 3); vehicle = 0.5% MC, 0.1% Tween-80, 99.4% water (5 mL/kg).

h Male cynomolgus monkeys (*n* = 3); vehicle = 0.5% MC, 0.1% Tween-80, 99.4% water (5 mL/kg).

i Male Göttingen Minipig (*n* = 3); vehicle = 10% HP-betaCD (5 mL/kg).

**Figure 4:**
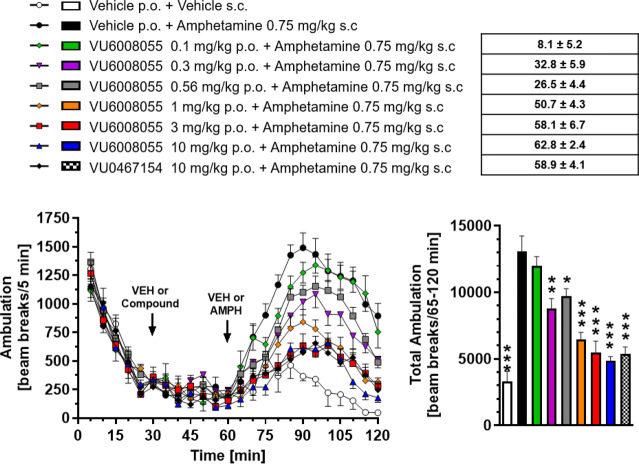
Systemic PO administration of **VU6008055** blocked amphetamine-induced hyperlocomotion in male Sprague–Dawley rats. (A) Time course of locomotor activity and (B) total locomotor activity during the 55 min period following amphetamine administration. Data are means ± SEM of 6–8 animals per group. *p < 0.05, **p < 0.01, ***p < 0.001 vs vehicle + amphetamine. Vehicle = 10% Tween 80 in H_2_O.

**Table 9:** Relationship Between Total (Mean *C*_brain_) and Unbound (Mean *C*_brain,u_) Brain Concentrations of **VU6008055** (**15p**) and PD Effects on Amphetamine (0.75 mg/kg, SC)-Induced Hyperlocomotion in Rats at 1.5 h

dose (mg/kg)	mean reversal of AHL (%)	mean *C*_plasma_ ^a^ (μM)	mean *C*_plasma,u_ ^b^ (nM)	mean *C*_brain_ ^a^ (μM)	mean *C*_brain,u_ ^c^ (nM)	brain:plasma mean *K*_p_	brain:plasma mean *K*_p,uu_
0.1	8.1	0.19	4.03	0.05	0.71	0.26	0.18
0.3	33	0.78	16.3	0.25	3.44	0.32	0.21
0.56	26	1.14	23.9	0.33	4.65	0.29	0.19
1	51	1.99	41.8	0.68	9.58	0.34	0.23
3	44	6.89	145	2.07	29.0	0.30	0.20
10	63	14.2	299	4.90	68.6	0.34	0.23

a At 1.5 h postadministration.

b Estimated unbound plasma concentration based on the rat *f*_u,plasma_ (0.020).

c Estimated unbound brain concentration based on the rat *f*_u,brain_ (0.015).

Next, we examined the effects of **VU6008055** in a preclinical model of glutamatergic hypofunction, which is also predictive of antipsychotic-like activity, specifically the noncompetitive *N*-methyl-d-aspartate (NMDA) glutamate receptor antagonist MK-801-induced hyperlocomotion (Figure 5). A 60 min pretreatment with **VU6008055** (0.3–3 mg/kg, p.o.) robustly blocked the hyperlocomotion induced by acute administration of MK-801 (0.18 mg/kg, s.c.), at the 3 mg/kg dose in rats. In comparison, **VU6008055** was more efficacious at attenuating the hyperlocomotor effects induced by amphetamine (MED = 0.3 mg/kg) than the MK-801 effects (MED = 3 mg/kg). Finally, we evaluated the potential for the development of tolerance in the rat AHL model when **VU6008055** was administered orally twice daily for 7 days (Figure 6). Following this chronic dosing regimen, **VU6008055** (3 mg/kg) produced an approximately 50.6% attenuation of amphetamine-induced hyperlocomotion that was similar in magnitude to the 45.7% attenuation observed after a single acute dose of 3 mg/kg of **VU6008055**. Thus, we did not observe the induction of tolerance to the antipsychotic-like activity of **VU6008055** with chronic dosing.

**Figure 5:**
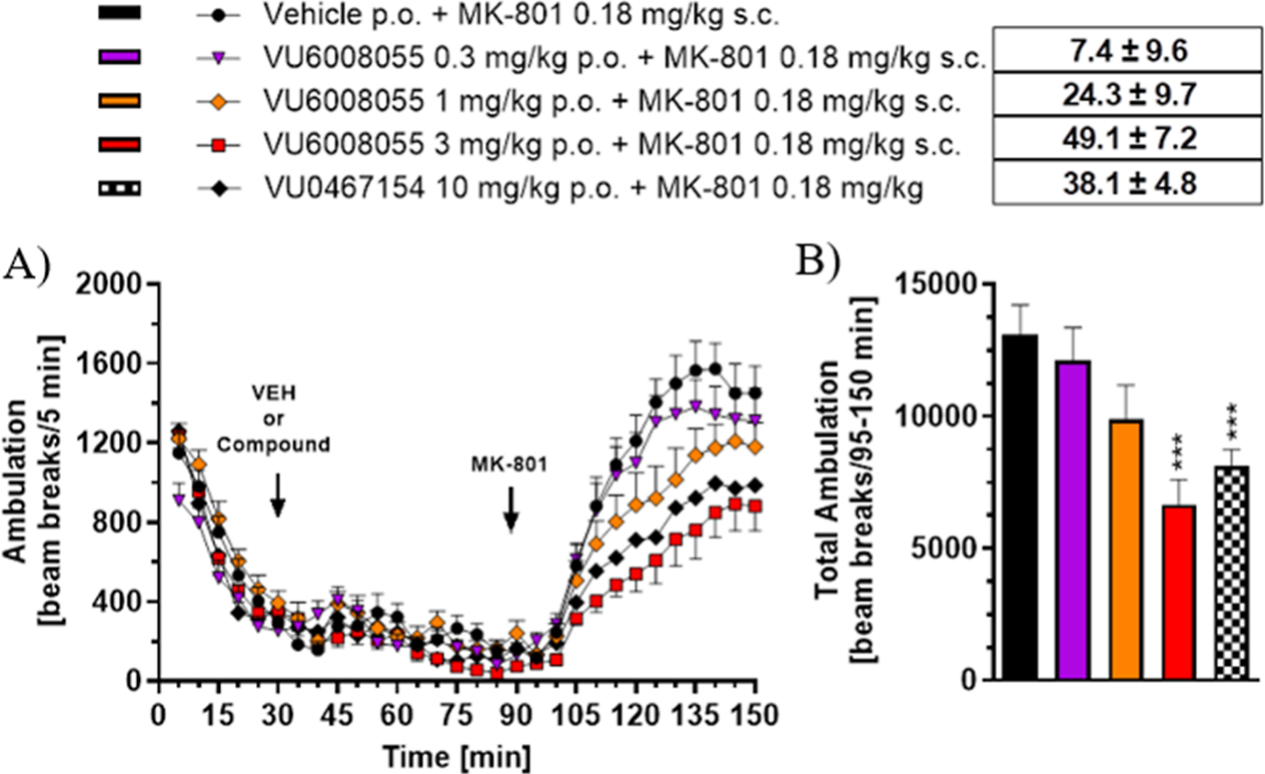
Systemic PO administration of **VU6008055** attenuated MK-801-induced hyperlocomotion in male Sprague–Dawley rats. (A) Time course of locomotor activity and (B) total locomotor activity during the 55 min period following MK-801 administration. Data are means ± SEM of 10–12 animals per group. ***p < 0.001 vs vehicle + MK-801. Vehicle = 10% Tween 80 in H_2_O.

**Figure 6:**
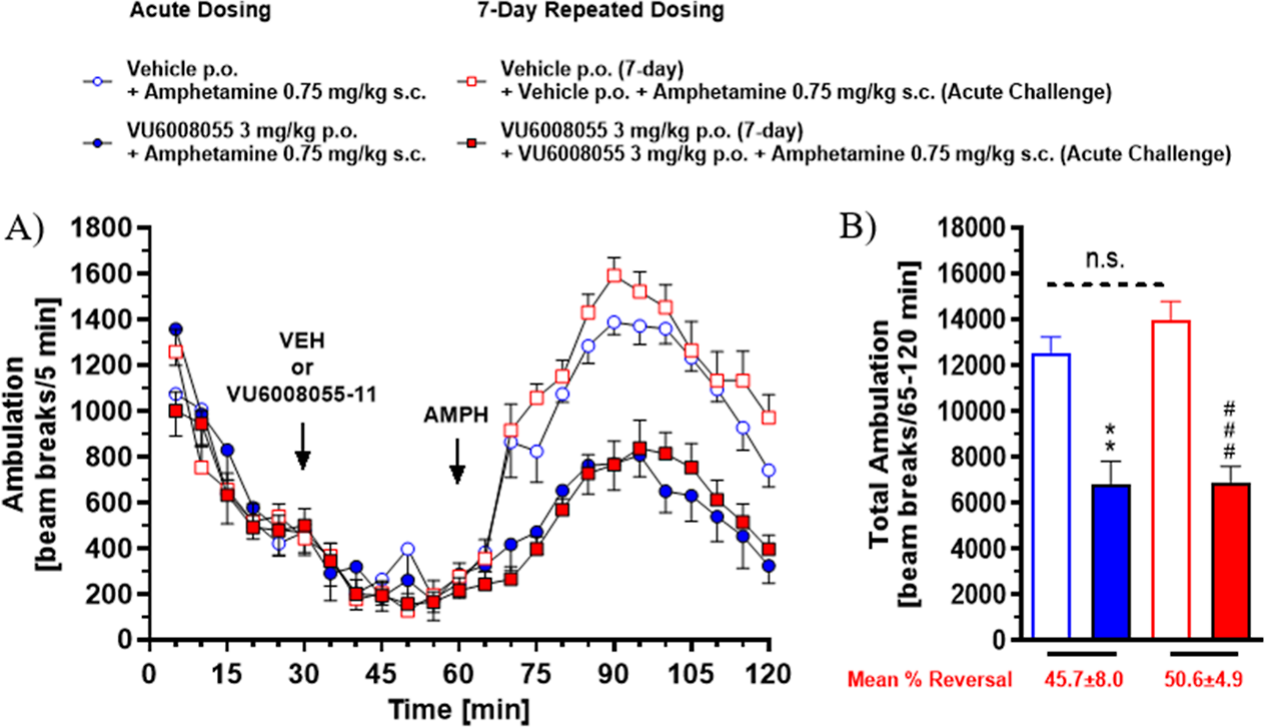
Seven day, twice daily repeated PO dosing of **VU6008055** does not alter its ability to block amphetamine-induced hyperlocomotion in male Sprague–Dawley rats. (A) Time course of locomotor activity and (B) total locomotor activity during the 55 min period following amphetamine administration. Rats were pretreated acutely or repeatedly (7 days, twice daily) by oral gavage with vehicle or **VU6008055** (3 mg/kg). On day 8, vehicle or **VU6008055** (3 mg/kg) was administered orally after 30 min by subcutaneous administration of amphetamine (0.75 mg/kg). Data are means ± SEM of 6 (blue curves, acute dosing) or 12 (red curves; repeated dosing) animals per group. **p < 0.01 vs vehicle + amphetamine (acute dosing), ^###^ *p* < 0.001 vs vehicle + amphetamine (7 day repeated dosing). Vehicle = 10% Tween 80 in H_2_O.

Another well-established preclinical assay used to screen and measure antipsychotic-like activity is the conditioned avoidance response (CAR) model.^45,46^ A feature that distinguishes all clinically approved antipsychotic drugs from other psychotherapeutics is their ability to disrupt CAR. Generally, rats treated with noncataleptic doses of antipsychotic drugs fail to acquire an avoidance response to a conditioned stimulus (e.g., tone and/or light), while their escape response to an unconditioned stimulus (e.g., foot shock) is unaffected. Given the robust efficacy demonstrated by **VU6008055** in both hyperlocomotion models, we tested our selective M_4_ PAM in CAR utilizing the atypical antipsychotic risperidone as a positive control (Figure 7). As expected, rats treated with risperidone displayed disrupted avoidance responses while maintaining their escape response when presented with a mild electric foot shock, whereas **VU6008055** had only a moderate effect on avoidance and escape responses at 30 mg/kg. When combined with a threshold dose of risperidone, **VU6008055** affected avoidance and escape responses to a larger degree at lower doses.

**Figure 7:**
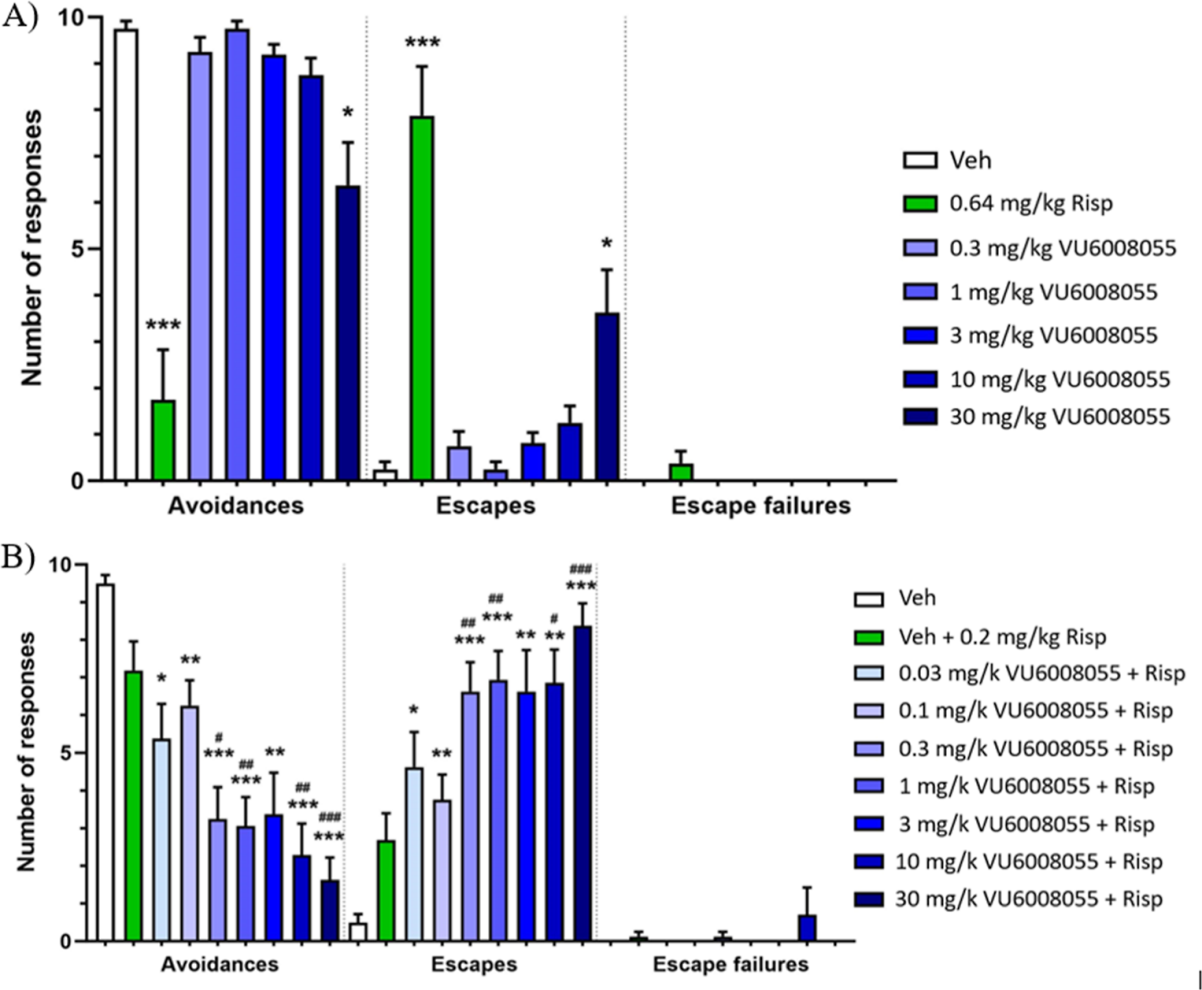
Effects of **VU6008055** in the CAR task in male Wistar-Han rats. (A) Effect of **VU6008055** 0.3–30 mg/kg in the CAR task in male Wistar–Han rats. A fully effective 0.64 mg/kg dose of risperidone used as a positive control for the assay. (B) Effect of **VU6008055** 0.03–30 mg/kg in the CAR task in male Wistar-Han rats in the presence of a threshold dose of 0.2 mg/kg risperidone. Data are expressed as means +SEM for groups of 8–16 rats. Comparisons by Brown–Forsythe and Welch test. Significant differences from the vehicle-treated controls are denoted by *p < 0.01, **p < 0.01, ***p < 0.001. Significant differences from Risperidone alone are denoted by #*p* < 0.05, ##*p* < 0.01, ###*p* < 0.001. Risperidone was dosed 30 min prior to testing. Other groups were dosed 60 min prior to testing.

With efficacy observed in two different, but dissociable, pharmacological models of hypoglutamatergic and hyperdopaminergic activity, we next conducted IV/PO PK in multiple preclinical species in parallel (Table 8). **VU6008055** displayed favorable oral bioavailability in dog as well as nonhuman primate (NHP) (54% and 44%, respectively) at 3 mg/kg doses and minipig (50%) at a 5 mg/kg dose. Moreover, we observed low plasma clearance in all three species (dog and NHP CL_p_ = 5.0 mL/min/kg; minipig CL_p_ = 7.7 mL/min/kg). Similar to rat, the volume of distribution was moderate in all higher species (dog *V*_ss_ = 1.4 L/kg; NHP *V*_ss_ = 0.9 L/kg; minipig *V*_ss_ = 2.2 L/kg), and elimination half-life was acceptable (dog *t*_1/2_ = 4.17 h; NHP *t*_1/2_ = 4.54 h; minipig *t*_1/2_ = 5.5 h).

### In-Depth DMPK Profiling^44^

Utilizing microsomes and cryopreserved hepatocytes, the metabolic stability of **VU6008055** was assessed in rat, dog, NHP, human, and minipig (hepatocytes only) at concentrations up to 1 μM. **VU6008055** displayed low intrinsic clearance in minipig (14.8 mL/min/kg), rat (25.2 mL/min/kg), and human (<5.17 mL/min/kg) hepatocytes as well as rat (35.8 mL/min/kg) and human (5.17 mL/min/kg) liver microsomes. Moderate clearance was observed in both NHP microsomes (29.0 mL/min/kg) and hepatocytes (23.5 mL/min/kg), while high clearance was observed in dog hepatocytes (38.3 mL/min/kg) with low to moderate clearance observed in dog microsomes (15.5 mL/min/kg). Next, liver microsomes (human or NHP) in combination with alamethicin (to enhance Phase II metabolism) were utilized to evaluate Phase I and Phase II metabolism. Clearance was assessed in the presence of cofactors NADPH (Phase I) and/or UDPGA (Phase II). After 1 h of incubation, human liver microsomes displayed >85% of **VU6008055** remaining, regardless of cofactor added. Conversely, NHP liver microsomes displayed high turnover in the presence of NADPH (17% remaining), while only minimal turnover was noted in the presence of UDPGA (80% remaining). These studies indicate that Phase I metabolism plays a major role in the metabolism of **VU6008055** in NHPs. Extrahepatic metabolism was also investigated using intestinal microsomes (rat, dog, NHP, and human) in the presence of cofactors NADPH and/or UDPGA. **VU6008055** was stable in all four species tested with >90% of compound remaining after 1 h.

To further elevate **VU6008055** toward candidate status and into IND-enabling studies, we performed additional studies to understand the compound’s CYP profile to minimize drug-drug interactions (DDIs) in the clinic. The first study assessed the ability of **VU6008055** to induce the expression of CYP1A, CYP2B6, and CYP3A4 by gene expression measurement (mRNA) in cryopreserved hepatocytes from three individual donors. This study indicated that **VU6008055** induces CYP2B6 and CYP3A4 at concentrations ≥3 μM, a concentration 41-fold above the human M_4_ PAM EC_50_ potency.

As **VU6008055** is a nitrogen-rich aromatic heterocycle, the involvement of aldehyde oxidase (AO)-mediated metabolism was assessed in both human and monkey liver S9 fractions in the presence and absence of AO inhibitors.^44^ AO-mediated metabolism catalyzes the oxidation of nitrogen-containing aromatic heterocycles. Incorporation of azaheterocycles into small-molecule drug candidates is a common strategy to circumvent CYP_450_ metabolism; thus, AO metabolism is now frequently encountered among drug discovery and development programs.^47,48^ After 90 min, no turnover was observed in either species, suggesting that **VU6008055** is not a substrate of AO metabolism.

In parallel to these CYP and AO studies, we performed multispecies metabolite identification (MetID) studies in cryopreserved hepatocytes (rat, dog, minipig, NHP, human) and observed comparable coverage of metabolites across human and NHP. Across all species, there was a total of 10 metabolites identified with only 9 metabolites associated with the human and NHP profiles. For human, metabolic turnover was moderate (59.1% parent remaining after 4 h) with metabolite MT4 (7.89%) and MT5 (29.6%) identified as the predominant metabolites (based on MS peak areas; Figure 8). Although NHP afforded a metabolic profile quite similar to human, turnover proved to be extensive with little parent drug remaining at 4 h (23.5% parent remaining), reiterating the high turnover observed in the previously discussed Phase I metabolism study. Additionally, the predominate NHP metabolites were MT2 (6.77%), MT4 (3.2%), and MT5 (48.9%). Minipig hepatocytes also displayed strong turnover (18.8% parent remaining), while moderate metabolic turnover was observed in both rat and dog (41.4% and 71.6% parent remaining, respectively); however, the latter three species produced species-specific metabolites (MT3).

**Figure 8:**
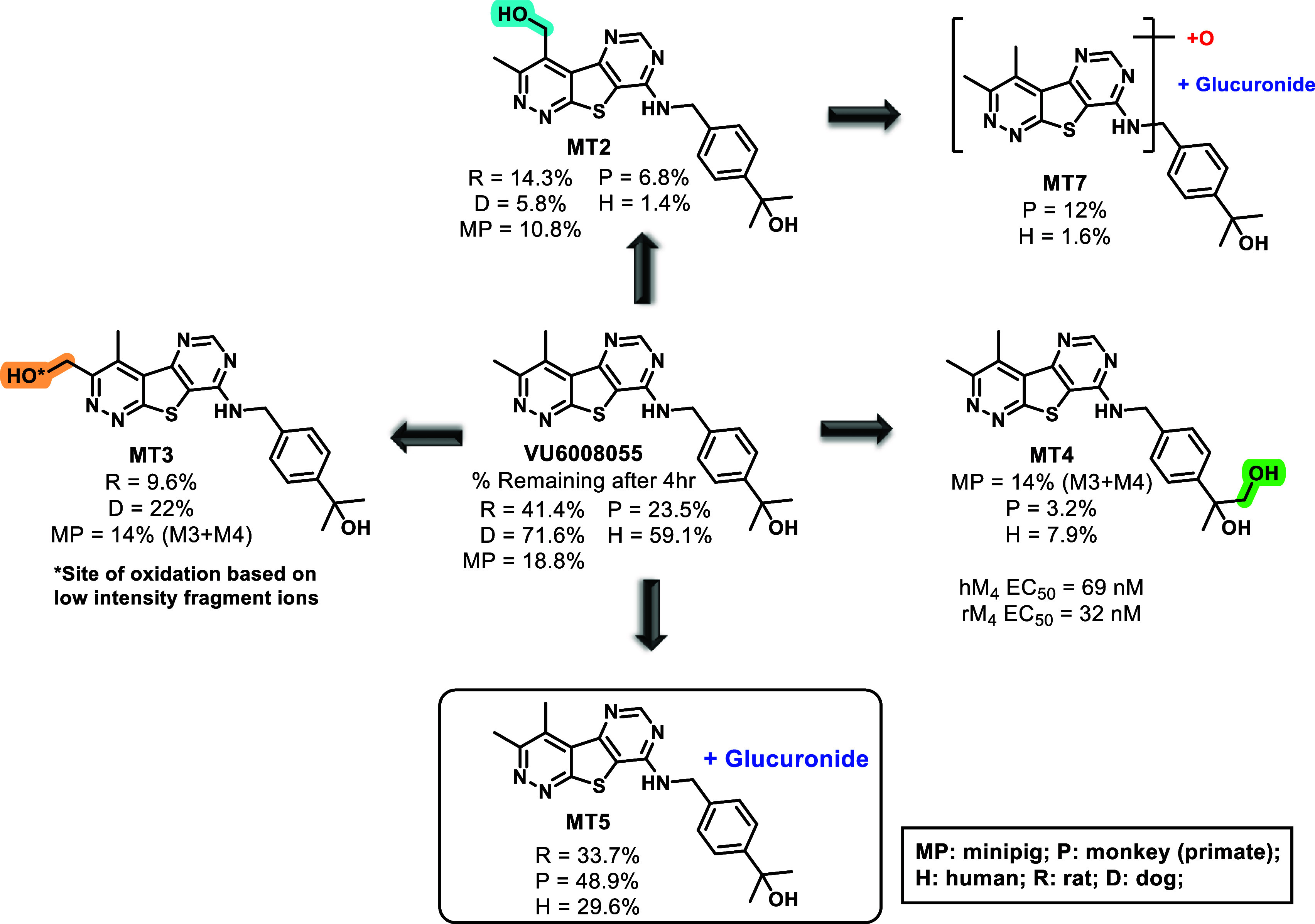
Structures of most prominent metabolites of **VU6008055** from multispecies hepatocyte MetID. Potency was determined for the most abundant, nonglucuronide human metabolite (**MT4**) using a calcium mobilization assay.

Finally, we investigated the fraction of CYP-mediated metabolism of **VU6008055** using a substrate depletion approach employing cryopreserved pooled hepatocytes from multiple species (human, rat, dog, and NHP) in the presence and absence of a CYP_450_ inhibitor, 1-aminobenzotriazole (ABT). Rat and dog had the highest fraction of CYP-mediated metabolism (93% and 87% respectively); however, both NHP and human showed >50% non-CYP-mediated metabolism (56% and 51% respectively). These data corroborate the high levels of glucuronidation (a Phase II metabolism process) observed for both NHP and human in the MetID study.

With a multitude of data in hand from several preclinical species, we next turned to human PK and dose predictions using two diverse approaches: in vitro–in vivo clearance extrapolation (IVIVE) and physiologically based pharmacokinetic modeling (PBPK). Based on the IVIVE scaling approach, humans are predicted to have low clearance (CL_hep_ = 0.77 mL/min/kg) with a low to moderate volume of distribution (*V*_ss_ = 0.44 L/kg) and a moderate half-life (*t*_1/2_ = 6.6 h). The IVIVE approach also predicted a moderate to high oral bioavailability (% *F* = 48%) and a once daily dose (QD) of 820 mg or a twice daily dose (BID) of 640 mg. Based on the PBPK scaling approach, humans were predicted to have low clearance (CL_hep_ = 0.77 mL/min/kg) with a low to moderate volume of distribution (*V*_ss_ = 0.74 L/kg) and a moderate half-life (*t*_1/2_ = 11.5 h). The PBPK approach also predicted a moderate to high oral bioavailability (% *F* = 38–45%) and a once daily dose of 850 mg or a twice daily dose of 410 mg. The human PK predictions based on the IVIVE and PBPK approaches are well aligned and suggest that **VU6008055** is likely suitable for once or twice daily dosing.

### Translational Biomarker Strategies

#### Pharmacologic Magnetic Resonance Imaging

To comprehensively understand the effects of selective potentiation of M_4_ on region-specific brain activation, **VU6008055** alone and in combination with amphetamine were evaluated using pharmacologic magnetic resonance imaging (phMRI) techniques, specifically changes in cerebral blood volume (CBV) using previously reported methods (see experimental timeline Figure 10a).^11^ This imaging approach allowed for the assessment of whether the efficacy of **VU6008055** observed in attenuating amphetamine-induced behavioral activation in rats could be recapitulated using an imaging modality that offers translatability from rodent preclinical studies into clinical studies in humans. As shown in the group-averaged CBV maps (Figure 9) and regional CBV time courses (Figure 10b), amphetamine (1 mg/kg s.c.) robustly increased CBV in both the caudate putamen and nucleus accumbens, brain regions with a high expression level of the dopamine transporter, as well as in extrastriatal areas, including the prefrontal and cingulate cortices, medial dorsal thalamic nucleus, and the ventral posterior lateral and ventral posterior medial thalamic nuclei in rats. In addition, the finding shown in the group-averaged CBV maps (Figure 9) and regional CBV time courses (Figure 10b) also demonstrate that a 30 min pretreatment with either a dose of 1 or 10 mg/kg p.o. of **VU6008055** dose-dependently attenuated amphetamine-evoked increases in CBV in the caudate putamen (*F*_2,25_ = 6.571, *p* < 0.01), nucleus accumbens (*F*_2,25_ = 6.268, *p* < 0.01), prefrontal cortex (*F*_2,26_ = 6.485, *p* < 0.01), cingulate cortex (*F*_2,26_ = 4.268, *p* < 0.05), medial dorsal thalamic nucleus (*F*_2,26_ = 3.657, *p* < 0.05), and ventral posterior lateral/ventral posterior medial thalamic nuclei (*F*_2,26_ = 6.578, *p* < 0.01). In addition, there were also no significant CBV responses detected in groups treated with vehicle/vehicle (Figure 10c) or a dose of 10 mg/kg po of **VU6008055** when administered alone. These data also confirmed that similar to inhibiting amphetamine-induced hyperlocomotion, **VU6008055** also blocked amphetamine-induced brain activation, supporting a role for M_4_ in the direct and indirect modulation of dopaminergic signaling across multiple brain regions. Collectively, these data suggest that changes in CBV, as assessed by phMRI may serve as a translational biomarker of M_4_ target engagement in clinical studies.

**Figure 9:**
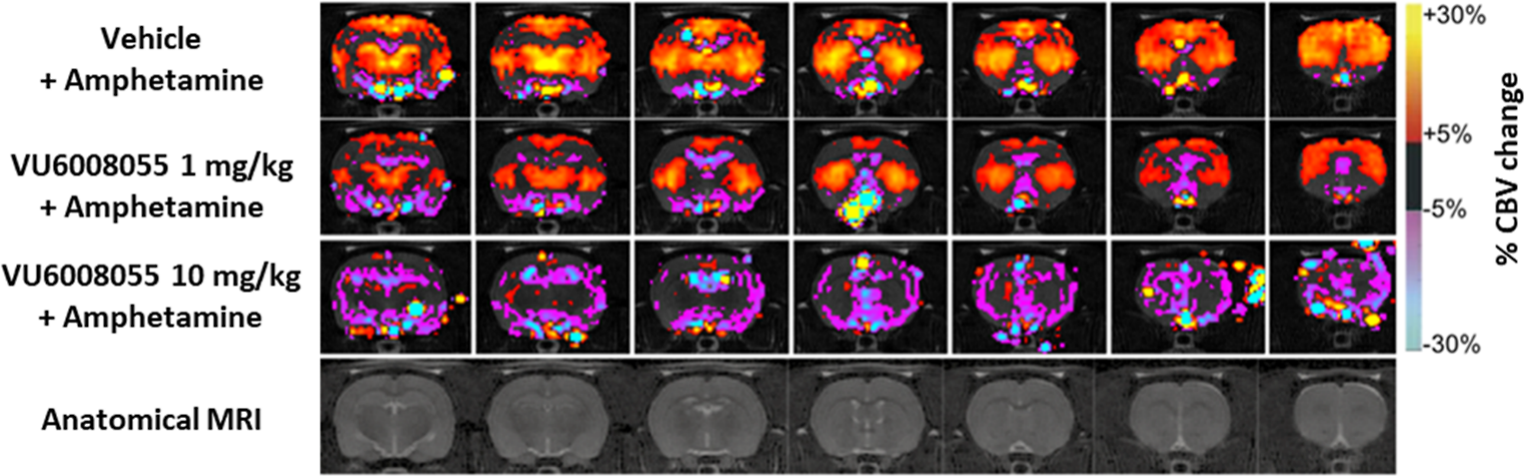
Systemic PO administration of **VU6008055** attenuates amphetamine-induced increases in CBV in male Sprague–Dawley rats as measured by phMRI. Group-averaged CBV maps illustrate the dose-related blockade of amphetamine (1 mg/kg s.c.)-induced changes in CBV by 1 and 10 mg/kg p.o. of **VU6008055**. Group-averaged CBV maps were generated by colorizing mean percent change in CBV for minutes 31–40 onto corresponding voxels of the template brain. Data are means ± SEM of 8–10 rats per group.

**Figure 10:**
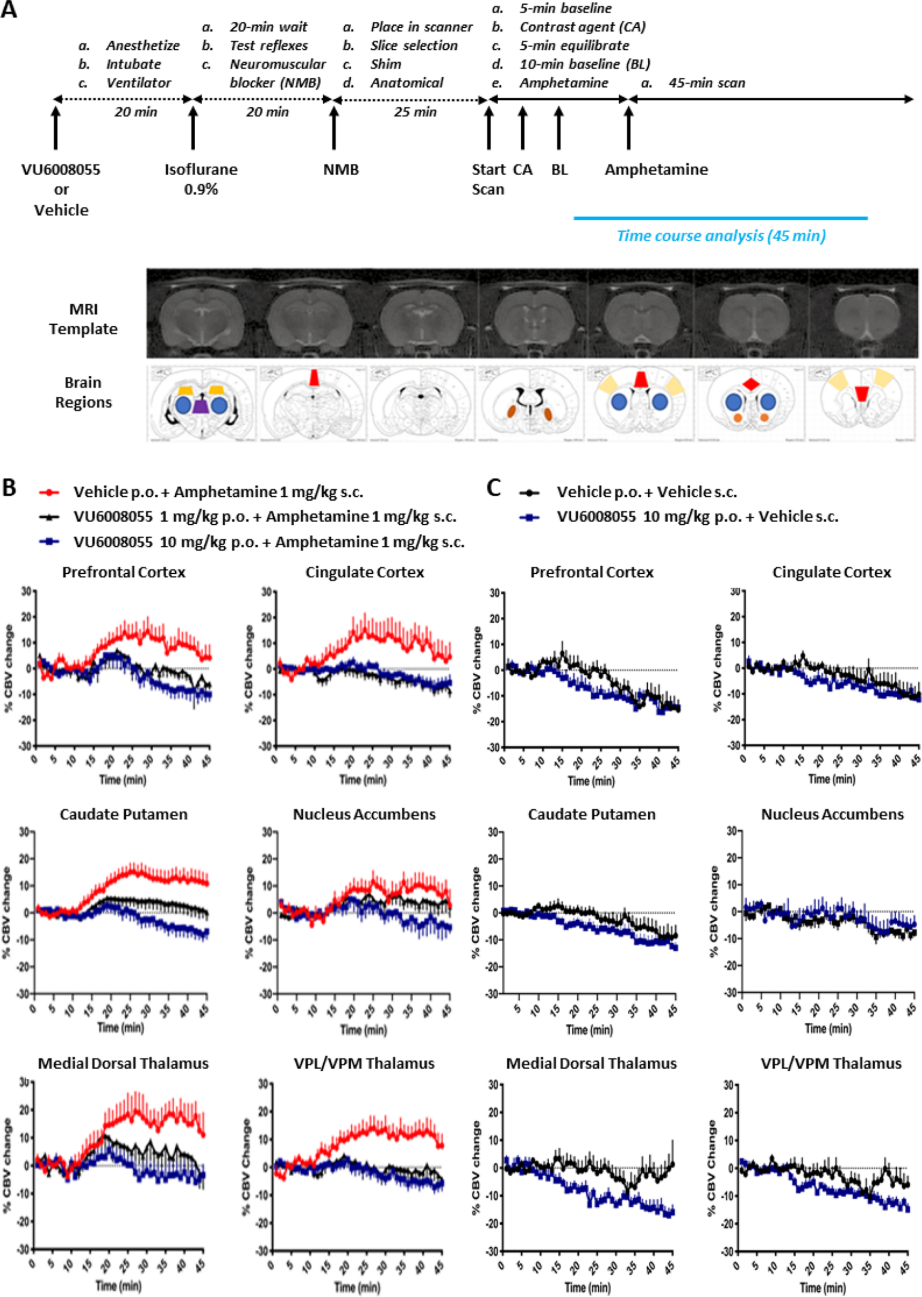
Time course of regional CBV changes following administration of **VU6008055** alone or in combination with amphetamine in male Sprague–Dawley rats. (A) Experimental timeline, structural MRI template, and identified brain regions assessed for CBV changes. Time courses of percent CBV changes for each region-of-interest (average of left and right hemispheres) following (B) administration of vehicle + amphetamine (red trace), **VU6008055** 1 mg/kg + amphetamine (black trace), and **VU60080555** 10 mg/kg + amphetamine or (C) vehicle + vehicle (black trace) and **VU6008055** + vehicle (blue trace). Amphetamine was injected at time point 10 min. Statistical comparisons of the 31–40 min bin were made using one-way ANOVA and Dunnett’s post hoc test (B) or one-tailed Mann–Whitney *U* test (GraphPad Prism 8.0). Data are presented as mean ± SEM of 8–10 animals per group.

### Quantitative EEG

Finally, since changes in quantitative electroencephalography (qEEG) have been reported to serve as a translational neurophysiological marker of different arousal states across species that can indicate therapeutic or adverse effects, we evaluated the dose-related effects of selective potentiation of M_4_ by **VU6008055** on changes in qEEG using previously reported methods by our group.^49^ As reported in the Supporting Information, we implanted rats with transmitters for telemetric recording of EEG (DSI), electromyographic (EMG), and motor activity; then, we collected EEG and EMG data using Dataquest A.R.T. 4.3 software (DSI) with a continuous sampling rate of 500 Hz. Blinded observers manually scored each 10 s epoch using Neuroscore 3.0 software as awake, NREM or REM sleep based on accepted characteristic oscillatory patterns.^49^ State-dependent relative power spectra from frontal electrodes were computed in 1 Hz bins from 0.5 to 100 Hz using a Fast Fourier Transformation and calculated as a percent of total power as previously described.^49^ Next we examined the pharmacological effects of **VU6008055** on arousal only during the epoch in which rats were awake. The power spectrum 1–2 h post dosing was expressed as the percent change within each respective 1 Hz interval from the 1 h interval prior to dosing (baseline). **VU6008055**-induced qEEG changes were represented as changes in power bands according to convention as delta (0.5–4 Hz), theta (4–8 Hz), alpha (8–13 Hz), beta (13–30 Hz), low gamma (30–50 Hz), and high gamma (50–100 Hz).^49^ As shown in Figure 11, qEEG analysis revealed that **VU6008055** produced a robust, dose-dependent increase in high gamma power, an EEG marker of increased arousal, during wake periods at doses of 0.3 mg/kg (78–79, 80–100 Hz), 1 mg/kg (78–100 Hz), 3 mg/kg (89–100 Hz), and 10 mg/kg (80–100 Hz; *p* < 0.05 vs vehicle). These data demonstrate that qEEG measures may be useful translational biomarkers for assessing target engagement for the M_4_ PAM mechanism.

**Figure 11:**
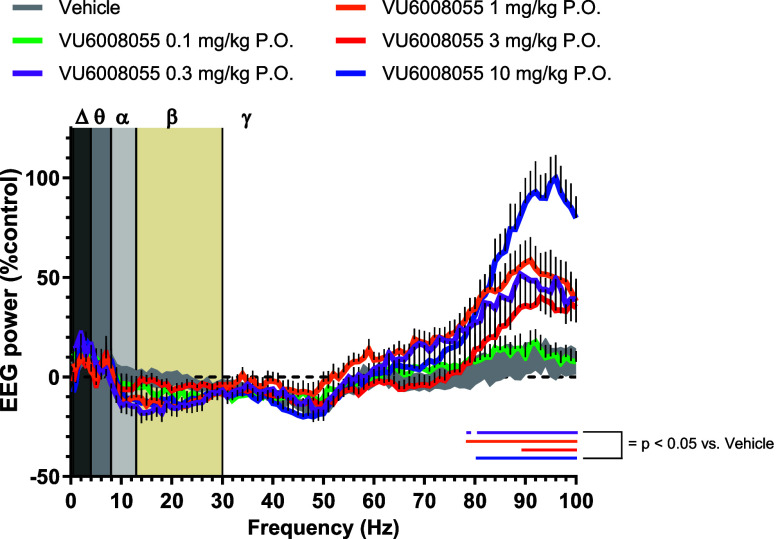
Systemic PO administration of **VU6008055** increases arousal in male Sprague–Dawley rats. The changes in relative spectral power in the frontal cortex (% change from baseline, BL) during waking epochs within the 1–2 h period following dosing with **VU6008055** are shown. Relative power was summed in 1 Hz bins (0.5–100 Hz) from all 10 s waking epochs and expressed as a percent change (±SEM) from respective power within the same frequency bin during waking epochs from the 1 h BL period prior to dosing. Gray/tan vertical bars represented frequency bands (Δ, delta 0.5–4 Hz; θ theta 4–8 Hz; α alpha, 8–13 Hz; β beta, 13–30 Hz; γ gamma 30–100 Hz). Corresponding colored horizontal dots/lines represented the frequencies at each dose that were statistically different from vehicle-treated rats. Vehicle = 10% Tween 80 in H_2_O.

## Conclusion

In summary, a scaffold hopping exercise based on a “tie-back” strategy previously utilized in our laboratory resulting in the discovery of M_4_ PAM **1 (VU6007215)** was applied to previously disclosed M_4_ PAM **4 (VU0467485)**. This approach proved to be a successful strategy to generate tricyclic, highly potent (hM_4_ EC_50_ < 100 nM) M_4_ PAM analogs devoid of the classic β-amino carboxamide moiety. Of the compounds analyzed, **VU6008055** (**15p**) displayed a superior overall profile that supported continued progression. Not only was **VU6008055** highly potent on human and rat M_4_, but the compound was also highly selective over the other mAChR evaluated. Additionally, **VU6008055** exhibited a low-to-moderate in vitro predicted hepatic clearance profile in human and low in vivo plasma clearance in rat. This was a substantial improvement over previously reported tricycle **VU6007215 (1)** (human CL_hep_ = 18 mL/min/kg; rat CL_hep_ = 63 mL/min/kg).^23^ **VU6008055** displayed a similar CNS distribution of unbound drug when compared to **VU6007215** as well as modest brain and plasma fraction unbound. **VU6008055** was moderately brain-penetrant and was not a substrate for the efflux transporter P-gp; however, it is a potential substrate of efflux transporter BCRP. **VU6008055** also displayed an excellent CYP inhibition profile with IC_50_’s ≥ 15 μM, a great improvement over predecessor **VU6008677** (**2**) (CYP 1A2 IC_50_ < 100 nM). Unlike **VU6016235** (**3**) (dog: CL_p_ = 24 mL/min/kg, % *F* = 25%), **VU6008055** exhibited excellent multispecies IV/PO PK (rat: CL_p_ = 4.7 mL/min/kg, % *F* = ≥ 89%; dog: CL_p_ = 5.0 mL/min/kg, % *F* = 54%; NHP: CL_p_ = 5.0 mL/min/kg, % *F* = ≥ 44%). Due to its attractive DMPK profile and improved physicochemical properties, **VU6008055** was progressed further into in vivo pharmacokinetic/pharmacodynamic (PK/PD) profiling. **VU6008055** showed robust efficacy in two preclinical models of antipsychotic activity (AHL and MK-801-induced hyperlocomotion). Compared to predecessor analog **VU0467485** (MED = 10 mg/kg), **VU6008055** was superior at blocking AHL in rats (MED of 0.3 mg/kg).^14^ Moreover, chronic administration of **VU6008055** did not induce tolerance in the rat AHL model. Evaluation in the CAR model revealed that, unlike typical and atypical antipsychotics, **VU6008055** did not diminish avoidance responses in rats at doses effective in AHL. Yet, higher doses of **VU6008055** or the presence of risperidone increased the effect of **VU6008055** in the CAR model. In depth DMPK analysis revealed that **VU6008055** was metabolically stable in hepatocytes and microsomes and was not a substrate of AO-metabolism. CYP induction studies showed that **VU6008055** induced CYP2B6 and CYP3A4 at concentrations well above the human M_4_ PAM EC_50_ potency (≥3 μM).

Finally, we evaluated the utility of phMRI and qEEG as biomarker strategies to assess M_4_ target engagement in clinical studies. In the phMRI studies, **VU6008055** dose-dependently blocked amphetamine-brain activation as determined by changes in CBV. Similarly, in the qEEG studies, **VU6008055** exhibited a dose-dependent increase in high gamma power correlating to increased arousal. These data indicated that phMRI and qEEG may be useful biomarker strategies to effectively determine target engagement in a clinical setting. Unfortunately, **VU6008055** was predicted to have a human dose projection of 820–850 mg QD or 410–640 mg BID. The high dose prediction is mostly driven by suboptimal PK parameters, mainly moderate bioavailability (between 40 and 50%) and low free fraction (0.7%) in higher species. Additionally, solubility and formulation challenges resulted in suboptimal exposure levels. Because of these, the human target total plasma concentration is 11 μM, and the dose to achieve this high concentration is between 820 and 850 mg QD. Despite **VU6008055** displaying clean off-target and safety/toxicity profiles, the potential risk of drug-induced liver injury associated with high daily dosing regiments prevented further development of **VU6008055**.^50^ Nevertheless, given its exceptional pharmacology and PK/PD profiles, **VU6008055** can serve as another valuable tool compound to study selective M_4_ activation in vivo. Next generation M_4_ PAMs without the limitations of **VU6008055** are currently under development with Neumora Therapeutics, and updates will be provided in due course.

## Methods

### General Information

^44^ All chemicals were purchased from commercial vendors and used without further purification. All NMR spectra were recorded on a 400 MHz AMX Bruker NMR spectrometer. ^1^H and ^13^C chemical shifts are reported as δ values in ppm downfield with the deuterated solvent as the internal standard. Low resolution mass spectra were obtained on an Agilent 6120/6150 or Waters QDa (Performance) SQ MS with the ESI source. High resolution mass spectra were obtained on an Agilent 6540 UHD Q-TOF with the ESI source. Normal phase column chromatography was performed on a Teledyne ISCO CombiFlash R_*f*_+ system. For compounds that were purified on a Gilson preparative reversed-phase HPLC, the system comprised of a 333 aqueous pump with solvent selection valve, 334 organic pump, GX 271 or GX-281 liquid hander, two column switching valves, and a 155 UV detector. Solvents for extraction, washing, and chromatography were HPLC grade. All final compounds were found to be >95% pure by HPLC-MS analysis.

### Synthesis^44^

#### 5-Amino-3,4-dimethylthieno­[2,3-*c*]­pyridazine-6-Carboxamide (**10**)

A microwave vial was charged with 3-mercapto-5,6-dimethylpyridazine-4-carbonitrile (83 mg, 0.50 mmol), 2-chloroacetamide (70 mg, 0.75 mmol), sodium carbonate (162 mg, 1.5 mmol), and NMP (2 mL). The mixture was subjected to a microwave reactor at 100 °C for 1 h and then at 100 °C on benchtop overnight. The reaction mixtures were cooled to room temperature and poured onto ice water. The precipitate was collected, washed with cold water, and dried under vacuum to provide the title compound as a pale green powder (65 mg, 59%). ES-MS [M + 1]^+^: 223.1.

#### 3,4-Dimethylpyrimido­[4′,5′:4,5]­thieno­[2,3-*c*]­pyridazin-8-ol **(12)**

In a 500 mL round-bottom flask, a suspension of intermediate **10** (3.5 g, 15.7 mmol) in triethyl orthoformate (100 mL, 600 mmol) was heated to reflux. After 6 h, the reaction mixture was concentrated. To the residue was added additional triethyl orthoformate (100 mL, 600 mmol), and the reaction was heated at reflux. After 16 h, LCMS shows complete conversion. The reaction was concentrated to dryness under reduced pressure and azeotroped with toluene (2 × 100 mL). The material was carried through without further purification (3.5 g). ES-MS [M + 1]^+^: 233.2; ^1^H NMR (400 MHz, DMSO-*d*_6_) δ: 8.43 (s, 1H), 2.92 (s, 3H), 2.77 (s, 3H), OH proton not observable.

#### 8-Chloro-3,4-dimethylpyrimido­[4′,5′:4,5]­thieno­[2,3-*c*]­pyridazine (**14)**

To a suspension of intermediate **12** (3.0 g, 11.6 mmol) in 1,2-dichloroethane (58 mL) was added trimethylamine (2.4 mL, 17.4 mmol), followed by the slow addition of phosphorus oxychloride (35 mL, 375.5 mmol). The reaction mixture was heated to reflux. After 16 h, the reaction mixture was cooled to room temperature and concentrated under vacuum. The residue was suspended in DCM (150 mL), and trimethylamine (5 mL) was added. The resulting solution was filtered to remove insoluble phosphate salts. The filtrate was concentrated under reduced pressure to yield a dark brown residue, which was purified using flash chromatography on silica gel (0–50% EtOAc/DCM) to yield the title compound as a fluffy powder (1.63 g, 56% yield). ES-MS [M + 1]^+^: 251.0; ^1^H NMR (400 MHz, DMSO-*d*_6_): δ 9.33 (s, 1H), 3.02 (s, 3H), 2.83 (s, 3H); ^13^C NMR (100 MHz, CDCl_3_): δ 162.4, 157.5, 157.1, 155.8, 154.6, 136.2, 132.9, 127.2, 19.9, 14.5.

#### 2-(4-(((3,4-Dimethylpyrimido­[4′,5′:4,5]­thieno­[2,3-*c*]­pyridazin-8-yl)­amino)­methyl)­phenyl)­propan-2-ol (**15p**, **VU6008055**)

A solution of 8-chloro-3,4-dimethylpyrimido­[4′,5′:4,5]­thieno­[2,3-*c*]­pyridazine (1.98 g, 7.88 mmol), 2-(4-(aminomethyl)­phenyl)­propan-2-ol (2.12 g, 10.24 mmol), and DIEA (5.49 mL, 31.5 mmol) in NMP (39.4 mL) was subjected to a microwave reactor for 30 min at 120 °C. After cooling at room temperature, the mixture was diluted with DMSO and syringe-filtered to remove any insoluble salts. The crude material was purified using reverse phase HPLC to afford the title compound (1.78 g, 60% yield) as an off-white powder. ES-MS [M+1]^+^: 380.4. ^1^H NMR (400 MHz, DMSO-*d*_6_): δ 8.73 (t, *J* = 5.9 Hz, 1H), 8.68 (s, 1H), 7.41 (d, *J* = 8.4 Hz, 2H), 7.30 (d, *J* = 8.4 Hz, 2H), 4.95 (s, 1H), 4.75 (d, *J* = 5.8 Hz, 2H), 2.99 (s, 3H), 2.77 (s, 3H), 1.39 (s, 6H). ^13^C NMR (101 MHz, DMSO): δ 161.9, 157.0, 156.5, 155.2, 152.9, 149.4, 136.5, 134.7, 127.4, 126.9 (2C), 124.6 (2C), 116.1, 70.6, 43.4, 32.0 (2C), 19.4, 13.7; HR-MS (Q-TOF, ES+) calcd for C_20_H_21_N_5_OS, 380.1540; found, 380.1541.

### Molecular Pharmacology

#### Calcium Mobilization Assay

Compound-evoked increases to an EC_20_ concentration of acetylcholine (ACh) in intracellular calcium were measured using Chinese hamster ovary (CHO) cells stably expressing human, rat, dog, cyno, or minipig muscarinic receptors (M_1_–M_5_; M_2_ and M_4_ cells were cotransfected with G_qi5_). The stable cells were cultured in F12 medium containing 10% fetal bovine serum, 20 mM HEPES, 100 units/mL antibiotics/antimycotic, 0.5 mg/mL G418, and 0.2 mg/mL hygromycin (M_2_ and M_4_ G_qi5_ coexpressing cells only). All reagents used were from Life Technologies (Carlsbad, CA) unless otherwise noted.

Briefly, the day before the assay, cells (15,000 cells/20 μL/well) were plated in black-walled, clear-bottomed, 384 well plates (Greiner Bio-One, Monroe, NC) in the culture medium without G418 and hygromycin and then incubated overnight at 37 °C in the presence of 5% CO_2_. The next day, calcium assay buffer [Hank’s balanced salt solution (HBSS), 20 mM HEPES, 2.5 mM Probenecid, 4.16 mM sodium bicarbonate (Sigma-Aldrich, St. Louis, MO)] was prepared to dilute compounds, agonists, and Fluo-4-acetomethoxyester (Fluo-4-AM), fluorescent calcium indicator dye. Compounds were serially diluted 1:3 into 10-point concentration response curves in DMSO using the Bravo Liquid Handler (Agilent, Santa Clara, CA), transferred to a 384 well daughter plates using an Echo acoustic liquid handler (Beckman Coulter, Indianapolis, Indiana), and diluted in assay buffer to a 2× final concentration. The agonist plates were prepared using acetylcholine (ACh, Sigma-Aldrich, St. Louis, MO) concentrations for the EC_20_, EC_80,_ and EC_MAX_ responses by diluting in assay buffer to a 5× final concentration. The 2× dye solution (2.3 μM) was prepared by mixing a 2.3 mM Fluo-4-AM stock in DMSO with 10% (w/v) pluronic acid F-127 in a 1:1 ratio in an assay buffer. Using a microplate washer (BioTek, Winooski, VT), cells were washed with assay buffer 3 times to remove the medium. After the final wash, 20 μL of assay buffer remained in the cell plates. Immediately, 20 μL of the 2× dye solution (final, 1.15 μM) was added to each well of the cell plate using a Multidrop Combi dispenser (Thermo Fisher, Waltham, MA). After cells were incubated with the dye solutions for 50 min at 37 °C in the presence of 5% CO_2_, the dye solutions were removed and replaced with assay buffer using a microplate washer, leaving 20 μL of assay buffer in the cell plate, and the cell plate allowed to incubate for 10 min at 37 °C. The compound, agonist, and cell plates were placed inside the Functional Drug Screening System 7000 (FDSS7000, Hamamatsu, Japan) to measure the calcium flux. After establishment of a fluorescence baseline for 2–3 s (2–3 images at 1 Hz; excitation, 480 ± 20 nm; emission, 540 ± 30 nm), 20 μL (2×) of the test compound or vehicle was added to the cells, and the response was measured. 140 s later, 10 μL (5×) of an EC_20_ concentration of ACh (Sigma-Aldrich, St. Louis, MO) or vehicle was added to the cells, and the response of the cells was measured. Approximately 125 s later, an EC_80_ or EC_MAX_ concentration of ACh was added. Calcium fluorescence was recorded as fold over basal fluorescence, and raw data were normalized to the maximal response to ACh. Compound-evoked increase in calcium response in the absence of ACh agonist was determined as ago activity of PAMs. Compound-evoked increase in calcium response in the presence of ACh EC_20_ agonist was determined as potentiator activity of the PAM. Potency (EC_50_) and maximum response (% ACh Max) for compounds were determined using a four-parameter logistical equation using GraphPad Prism (La Jolla, CA) or the Dotmatics software platform (Woburn, MA)

$$y=bottom+\frac{top-bottom}{1+10^{\left(\log EC_{50}-A\right)Hill\;slope}}$$

where *A* is the molar concentration of the compound; bottom and top denote the lower and upper plateaus of the concentration–response curve; Hill slope is the Hill coefficient that describes the steepness of the curve; and EC_50_ is the molar concentration of the compound required to generate a response halfway between the top and bottom.

### Radioligand Binding Assay

Membranes were made from CHO cells stably expressing the rat or human M_4_ receptor (coexpressing G_qi5_). Radioligand competition binding assays were performed as previously described (Anderson et al., 2002) with minor modifications. In brief, M_4_ PAM, **VU6008055**, was serially diluted into assay buffer and added to each well of a 96-well plate, along with 10 μg/well cell membrane in the presence of 100 μM acetylcholine and approximately 500 pM [^3^H]-MK6884 (RC Tritec, Teufen, Switzerland). Following a 3 h incubation period on the shaker at room temperature, the membrane-bound ligand was separated from free ligand by filtration through glass fiber 96-well filter plates (Unifilter-96, GF/B; PerkinElmer, Boston, MA). Forty microliters of scintillation fluid was added to each well, and the membrane-bound radioactivity was determined by scintillation counting (TopCount; PerkinElmer Life and Analytical Sciences, Boston, MA). Nonspecific binding was determined using 10 μM VU6014442 (a structural analog of MK6884).

## Supplementary Material



## Notes

General methods for the synthesis and characterization for key compounds, experimental details for all assays performed (DMPK, behavioral pharmacology, and translational pharmacology), additional ancillary pharmacology data (Eurofins Panlabs and Cardiac Ion Channel Panel), and human dose projections (PDF)

## Abbreviations

ACh: acetylcholine
AD: Alzheimer’s disease
AHL: amphetamine-induced hyperlocomotion
AO: aldehyde oxidase
BHB: brain homogenate binding
BID: twice a day
CAR: conditioned avoidance response
CBV: cerebral blood volume
CL_hep_: hepatic clearance
CL_int_: intrinsic clearance
CNS: central nervous system
CYP: cytochrome P450
DDI: drug–drug interactions
DMPK: drug metabolism and pharmacokinetics
EMG: electromyographic
FDA: food and drug administration
hM4: muscarinic acetylcholine receptor subtype 4, human
IND: investigational new drug
mAChR: muscarinic acetylcholine receptor
MED: minimum effective dose
MetID: metabolite identification
NHP: nonhuman primate
PAM: positive allosteric modulator
P-gp: P-glycoprotein 1
PBL: plasma-to-brain levels
PD: pharmacodynamic
PK: pharmacokinetic
phMRI: pharmacologic magnetic resonance imaging
PPB: plasma protein binding
QD: once daily
qEEQ: quantitative EEG
rM4: muscarinic acetylcholine receptor subtype 4, rat
SAR: structure activity relationship
μW: microwave irradiated
