# Supplementary material for: Discovery of Pre-Clinical Candidate VU6008055/AF98943: A Highly Selective, Orally Bioavailable, and Structurally Distinct Tricyclic M4 Muscarinic Acetylcholine Receptor Positive Allosteric Modulator (PAM) with Robust In Vivo Efficacy
Source: ACS Chem Neurosci. 2025 May 26;16(11):2141–62. doi: 10.1021/acschemneuro.5c00277 (PMC12142581; doi:10.1021/acschemneuro.5c00277)
Supplement: Supplementary file 1 [file cn5c00277_si_001.pdf]

Supplemental Information for

Discovery of Pre-Clinical Candidate VU6008055/AF98943: A Highly Selective, Orally Bioavailable, and Structurally Distinct Tricyclic M_4 Muscarinic Acetylcholine Receptor Positive Allosteric Modulator with Robust *In Vivo* Efficacy

Julie L. Engers[a,b], Sean R. Bollinger[a,b], Alison R. Gregro[a,b], Rory A. Capstick[a,b], Paul K. Spearing[a,b], Madeline F. Long[a,b], James C. Tarr[a,b], Katherine J. Watson[a,b], Sichen Chang[a,b], Vincent B. Luscombe[a,b], Alice L. Rodriguez[a,b], Hyekyung P. Cho[a,b], Aidong Qi[a,b], Colleen M. Niswender[a,b,e,f], Michael Bubser[a,b], Robert W. Gould[a,b], William Hudson Robb[a,b], Nellie Byun[a,b,g], John Gore[g,h,i], Carrie K. Jones[a,b,f], Morten S. Thomsen[j], Thomas M. Bridges[a,b], Olivier Boutaud[a,b], P. Jeffrey Conn[a,b,f], Darren W. Engers[a,b], Craig W. Lindsley[a,b,c,d*], Kayla J. Temple[a,b*]

[a]Warren Center for Neuroscience Drug Discovery, Vanderbilt University, Nashville, TN 37232, USA
[b]Department of Pharmacology, Vanderbilt University School of Medicine, Nashville, TN 37232, USA
[c]Department of Chemistry, Vanderbilt University, Nashville, TN 37232, USA
[d]Department of Biochemistry, Vanderbilt University, Nashville, TN 37232, USA
[e]Vanderbilt Kennedy Center, Vanderbilt University School of Medicine, Nashville, TN 37232, USA
[f]Vanderbilt Brain Institute, Vanderbilt University School of Medicine, Nashville, TN 37232, USA
[g]Vanderbilt University Institute of Imaging Science, Vanderbilt University School of Medicine, Nashville, TN 37232, USA
[h]Department of Radiology and Radiological Sciences, Vanderbilt University Medical Center, Nashville, TN 37232, USA
[i]Department of Biomedical Engineering, Vanderbilt University, Nashville, TN 37232, USA
[j]Neuroscience Research, H. Lundbeck A/S, Valby, DK-2500, Denmark.

*Corresponding authors' email:
kayla.temple@vanderbilt.edu
craig.lindsley@vanderbilt.edu

Table of Contents

Experimental Synthetic Procedures and Spectroscopic Data S2
DMPK Methods S12
Lead Profiling Screen to Assess Off-Target Liabilities S16
Evaluation of Potential Cardiac Risks S18
In-vitro Determination of Brain Penetration Potential S18
Metabolic Stability S20
Multi-species Hepatocyte MetID S28
ADME-Tox: Genetic Toxicity S33
In vivo Behavioral Pharmacology S35
Pharmacologic MRI S37
EEG Procedures S38
Human Dose Predictions S39

Experimental Synthetic Procedures and Spectroscopic Data

General Instrumentation Methods.

All NMR spectra were recorded on a 400 MHz AMX Bruker NMR spectrometer. ^{1}H and ^{13}C chemical shifts are reported in δ values in ppm downfield with the deuterated solvent as the internal standard. Data are reported as follows: chemical shift, multiplicity (s = singlet, d = doublet, t = triplet, q = quartet, b = broad, m = multiplet), integration, coupling constant (Hz). Low resolution mass spectra were obtained on an Agilent 6120/6150 or Waters QDa (Performance) SQ MS with ESI source. *Method A (Agilent 6120/6150):* MS parameters were as follows: fragmentor: 70, capillary voltage: 3000 V, nebulizer pressure: 30 psig, drying gas flow: 13 L/min, drying gas temperature: 350 ºC. Samples were introduced via an Agilent 1290 UHPLC comprised of a G4220A binary pump, G4226A ALS, G1316C TCC, and G4212A DAD with ULD flow cell. UV absorption was generally observed at 215 nm and 254 nm with a 4 nm bandwidth. Column: Waters Acquity BEH C18, 1.0 x 50 mm, 1.7 um. Gradient conditions: 5% to 95% CH_3CN in H_2O (0.1% TFA) over 1.4 min, hold at 95% CH_3CN for 0.1 min, 0.5 mL/min, 55 ºC. *Method B (Agilent 6120/6150):* MS parameters were as follows: fragmentor: 100, capillary voltage: 3000 V, nebulizer pressure: 40 psig, drying gas flow: 11 L/min, drying gas temperature: 350 ºC. Samples were introduced via an Agilent 1200 HPLC comprised of a degasser, G1312A binary pump, G1367B HP-ALS, G1316A TCC, G1315D DAD, and a Varian 380 ELSD (if applicable). UV absorption was generally observed at 215 nm and 254 nm with a 4 nm bandwidth. Column: Thermo Accucore C18, 2.1 x 30 mm, 2.6 um. Gradient conditions: 7% to 95% CH_3CN in H_2O (0.1% TFA) over 1.6 min, hold at 95% CH_3CN for 0.35 min, 1.5 mL/min, 45 ºC. *Method C (Waters QDa (Performance) SQ MS):* MS parameters were as follows: cone voltage: 15 V, capillary voltage: 0.8 kV, probe temperature: 600° C. Samples were introduced via an Acquity I-Class PLUS UPLC comprised of a BSM, FL-SM, CH-A, and PDA. UV absorption was generally observed at 215 nm and 254 nm; 4 nm bandwidth. Column: Phenomenex EVO C18, 1.0 x 50 mm, 1.7 um. Column temperature: 55° C. Flow rate: 0.4 mL/min. Default gradient: 5% to 95% CH_3CN (0.05% TFA) in H_2O (0.05% TFA) over 1.4 min (curve 6), hold at 95% CH_3CN for 0.1 min. "Polar" (2% to 70% CH_3CN (0.05% TFA) in H_2O (0.05% TFA) over 0.8 min (curve 6), transition to 95% CH_3CN over 0.1 min (curve 6), hold at 95% CH_3CN for 0.6 min.) and "Non-Polar" (40% to 95% CH_3CN (0.05% TFA) in H_2O (0.05% TFA) over 1.4 min (curve 6), hold at 95% CH_3CN for 0.1 min.) gradients were also available. *Method D (Waters QDa (Performance) SQ MS):* MS parameters were as follows: cone voltage: 15 V, capillary voltage: 0.8 kV, probe temperature: 600° C. Samples were introduced via an Acquity I-Class PLUS UPLC comprised of a BSM, FL-SM, CH-A, and PDA. UV absorption was generally observed at 215 nm and 254 nm with a 4 nm bandwidth. Column: Phenomenex EVO C18, 1.0 x 50 mm, 1.7 um. Column temperature: 55° C. Flow rate: 0.4 mL/min. Default gradient: 5% to 95% CH_3CN in H_2O (5 mM NH_4HCO_3) over 1.4 min (curve 6), hold at 95% CH_3CN for 0.1 min. "Polar" (2% to 70% CH_3CN in H_2O (5 mM NH_4HCO_3) over 0.8 min (curve 6), transition to 95% CH_3CN over 0.1 min (curve 6), hold at 95% CH_3CN for 0.6 min.) and "Non-Polar" (40% to 95% CH_3CN

in H_2O (5 mM NH_4HCO_3) over 1.4 min (curve 6), hold at 95% CH_3CN for 0.1 min.) gradients were also available. High resolution mass spectra were obtained on an Agilent 6540 UHD Q-TOF with ESI source. MS parameters were as follows: fragmentor: 150, capillary voltage: 3500 V, nebulizer pressure: 60 psig, drying gas flow: 13 L/min, drying gas temperature: 275 ºC. Samples were introduced via an Agilent 1200 UHPLC comprised of a G4220A binary pump, G4226A 3 ALS, G1316C TCC, and G4212A DAD with ULD flow cell. UV absorption was observed at 215 nm and 254 nm with a 4 nm bandwidth. Column: Agilent Zorbax Extend C18, 1.8 µm, 2.1 x 50 mm. Gradient conditions: 5% to 95% CH_3CN in H_2O (0.1% formic acid) over 1 min, hold at 95% CH_3CN for 0.1 min, 0.5 mL/min, 40 ºC. For compounds that were purified on a Gilson preparative reversed-phase HPLC, the system comprised of a 333 aqueous pump with solvent selection valve, 334 organic pump, GX 271 or GX-281 liquid hander, two column switching valves, and a 155 UV detector. UV wavelength for fraction collection was user-defined, with absorbance at 254 nm always monitored. Method 1: Phenomenex Axia-packed Luna C18, 30 x 50 mm, 5 µm column. Mobile phase: CH_3CN in H_2O (0.1% TFA). Gradient conditions: 0.75 min equilibration, followed by user defined gradient (starting organic percentage, ending organic percentage, duration), hold at 95% CH_3CN in H_2O (0.1% TFA) for 1 min, 50 mL/min, 23 ºC. Method 2: Phenomenex Axia packed Gemini C18, 50 x 250 mm, 10 um column. Mobile phase: CH_3CN in H_2O (0.1% TFA). Gradient conditions: 7 min equilibration, followed by user defined gradient (starting organic percentage, ending organic percentage, duration), hold at 95% CH_3CN in H_2O (0.1% TFA) for 7 min, 120 mL/min, 23 ºC. Normal phase column chromatography was performed on a Teledyne ISCO CombiFlash® R*f*+ system. Solvents for extraction, washing and chromatography were HPLC grade. All final compounds were found to be >95% pure by LCMS analysis.

General Procedure for the Preparation of Analogs **13** (Scheme 1).

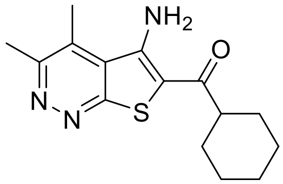

(5-Amino-3,4-dimethylthieno[2,3-*c*]pyridazin-6-yl)(cyclohexyl)methanone (11g). To a mixture of 3-mercapto-5,6-dimethylpyridazine-4-carbonitrile (200 mg, 1.21 mmol) in isopropyl alcohol (8 mL) was added 2-bromo-1-cyclohexylethan-1-one (175 µL, 1.45 mmol) and 10% potassium hydroxide (aq) (1.12 mL). After one hour, the reaction was concentrated in vacuo and used without further purification. ES-MS $[M+1]^+$: 290.2.

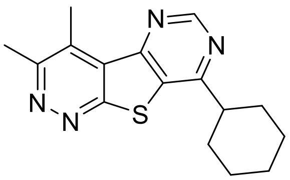

8-Cyclohexyl-3,4-dimethylpyrimido[4',5':4,5]thieno[2,3-*c*]pyridazine (13g, VU6009096). To a mixture of intermediate **11g** (350 mg, 1.21 mmol) and formamide (6.4 mL) in an open to air vial heated to 150 °C was added formamidine acetate (1.26 g, 12.1 mmol) in six equal portions over three hours. After three more hours, more formamidine acetate (1.26 g, 12.1 mmol) was added. After an additional 16 hours, more formamidine acetate (1.26 g, 12.1 mmol) was added. The reaction was cooled to room temperature before being diluted with water (100 mL) and extracted with dichloromethane (2 x 50 mL). All organic layers were combined, dried ($MgSO_4$) and concentrated in vacuo. Purification by reverse phase HPLC afforded the title compound (30 mg, 9% yield). ES-MS $[M+1]^+$: 299.2. ^{1}H NMR (400 MHz, $CDCl_3$) δ 9.35 (s, 1H), 3.13 (s, 3H), 3.01 (tt, *J* = 11.7, 3.5 Hz, 1H), 2.93 (s, 3H), 2.16 – 2.02 (m, 2H), 1.98 (dt, *J* = 12.9, 3.2 Hz, 2H), 1.85 (qd, *J* = 12.4, 3.3 Hz, 3H), 1.60 – 1.33 (m, 3H). ^{13}C NMR (101 MHz, $CDCl_3$) δ 169.9, 162.9, 157.1, 156.0, 155.2, 135.8, 131.3, 128.1, 46.4, 30.9 (2C), 26.3 (2C), 25.9, 20.0, 14.5. HR-MS (Q-TOF, ES+) calc'd for $C_{16}H_{18}N_4S$, 299.1325; found, 299.1324.

<u>General Procedure for the Preparation of Analogs **15** (Scheme 1).</u>

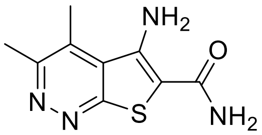

5-Amino-3,4-dimethylthieno[2,3-*c*]pyridazine-6-carboxamide (10). To a microwave vial charged with 3-mercapto-5,6-dimethylpyridazine-4-carbonitrile (83 mg, 0.50 mmol), 2-chloroacetamide (70 mg, 0.75 mmol), sodium carbonate (162 mg, 1.5 mmol) and NMP (2 mL). The mixture was subjected to a microwave reactor at 100 °C for 1 hour then 100 °C on bench top overnight. The reaction mixtures were cooled to room temperature and poured onto ice water. The precipitate was collected, washed with cold water and dried under vacuum to provide title compound as a pale green powder (65 mg, 59%). ES-MS $[M+1]^+$: 223.1.

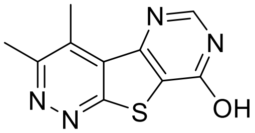

3,4-Dimethylpyrimido[4',5':4,5]thieno[2,3-*c*]pyridazin-8-ol (12). In a 500 mL round-bottomed flask, a suspension of intermediate **10** (3.5 g, 15.7 mmol) in triethyl orthoformate (100 mL, 600 mmol) was heated to reflux. After 6 hours, the reaction mixture was concentrated. To the residue was added additional triethyl orthoformate (100 mL, 600 mmol) and the reaction was heated at reflux. After 16 hours, LCMS shows complete conversion. The reaction was concentrated to dryness under reduced pressure and azeotroped with toluene (2 x 100 mL). The material was carried through without further purification (3.5 g). ES-MS $[M+1]^+$: 233.2; ^{1}H NMR (400 MHz, DMSO-d_6) δ 8.43 (s, 1H), 2.92 (s, 3H), 2.77 (s, 3H), OH proton not observable.

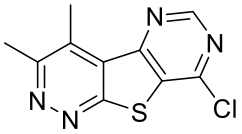

8-Chloro-3,4-dimethylpyrimido[4',5':4,5]thieno[2,3-*c*]pyridazine (14). To a suspension of intermediate **12** (3.0 g, 11.6 mmol) in 1,2-dichloroethane (58 mL) was added trimethylamine (2.4 mL, 17.4 mmol) followed by the slow addition of phosphorus oxychloride (35 mL, 375.5 mmol). The reaction mixture was heated to reflux. After 16 hours, the reaction mixture was cooled to room temperature and concentrated under vacuum. The residue was suspended in DCM (150 mL) and trimethylamine (5 mL) was added. The resulting solution was filtered to remove insoluble phosphate salts. The filtrate was concentrated under reduced pressure to yield a dark brown residue which was purified using flash chromatography on silica gel (0-50% EtOAc/DCM) to yield the title compound as a fluffy powder (1.63 g, 56% yield). ES-MS $[M+1]^+$: 251.0; ^{1}H NMR (400 MHz, DMSO-d_6) δ 9.33 (s, 1H), 3.02 (s, 3H), 2.83 (s, 3H); ^{13}C NMR (100 MHz, $CDCl_3$) δ 162.4, 157.5, 157.1, 155.8, 154.6, 136.2, 132.9, 127.2, 19.9, 14.5.

Preparation of 2-(4-(aminomethyl)phenyl)propan-2-ol.

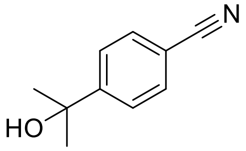

4-(2-Hydroxypropan-2-yl)benzonitrile. To a solution of 4-iodobenzonitrile (10.0 g, 43.7 mmol) in THF (218 mL) at -78 °C was added *n*-butyl lithium (2.5*M* in hexanes, 22.7 mL, 56.8 mmol) dropwise as to maintain the temperature below -70 °C. After 1 hour, acetone (32.0 mL, 436.6 mmol) was added while maintaining the temperature below -70 °C. The dry ice bath was removed. After 16 hours at room temperature, a saturated solution of NH_4Cl (100 mL) was added, followed by EtOAc (250 mL). The layers were separated. The aqueous layer was extracted with EtOAc (2 x 200 mL). The combined organic layers were washed with brine, dried (Na_2SO_4), filtered and concentrated. The residue was purified by flash column chromatography on silica gel (0-60% EtOAc/hexanes) to provide the title compound as a viscous oil (4.88 g, 69% yield). ES-MS $[M+1]^+$: 162.4; ^{1}H NMR (400 MHz, DMSO-d_6) δ 7.76 (dd, J = 10, 2 Hz, 2H), 7.66 (dd, J = 8.6, 2 Hz, 2H), 5.28 (s, 1H), 1.43 (s, 6H).

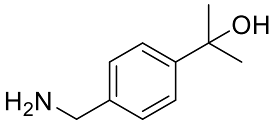

2-(4-(Aminomethyl)phenyl)propan-2-ol. To a solution of 4-(2-hydroxypropan-2-yl)benzonitrile (4.88 g, 30.3 mmol) in THF (200 mL) was added a solution of lithium aluminum hydride (2.0*M* in THF, 45.4 mL, 90.8 mmol) dropwise at 0 °C. After 30 min at 0 °C, the ice bath was removed and the reaction was heated to reflux. After 30 min, a creamy paste was formed. The heat was removed. At 0 °C, a saturated solution of Rochelle salt (50 mL) was added slowly followed by MeOH (50 mL). The mixture was stirred at room temperature for 1 hour and filtered through a pad of Celite which was further rinsed with 15% MeOH in DCM. The collected filtrate was dried ($MgSO_4$), filtered and concentrated. The residue was purified by flash column chromatography on silica gel using

a solution of DCM/MeOH/NH_4OH (10:89:1) with DCM as a co-solvent to provide the title compound as a white crystalline solid (4.25 g, 85% yield). ES-MS $[M+1]^+$: 166.3; ^1H NMR (400 MHz, DMSO-d_6) δ 7.38 (d, J = 8.3 Hz, 2H), 7.24 (d, J = 8.4 Hz, 2H), 4.93 (bs, 1H), 3.68 (s, 2 H), 1.41 (s, 6H), NH protons not observable; ^{13}C NMR (100 MHz, DMSO-d_6) δ 148.9, 142.1, 126.9, 124.7, 71.0, 45.9, 32.5.

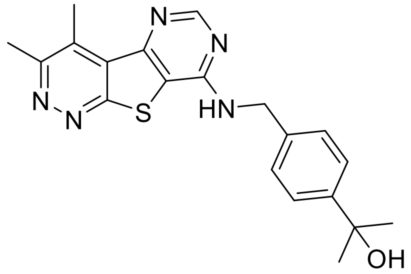

2-(4-(((3,4-Dimethylpyrimido[4',5':4,5]thieno[2,3-*c*]pyridazin-8-yl)amino)methyl)phenyl)propan-2-ol (15p, VU6008055). A solution of 8-chloro-3,4-dimethylpyrimido[4',5':4,5]thieno[2,3-*c*]pyridazine (1.98 g, 7.88 mmol,), 2-(4-(aminomethyl)phenyl)propan-2-ol (2.12 g, 10.24 mmol) and DIEA (5.49 mL, 31.5 mmol) in NMP (39.4 mL) was subjected to a microwave reactor for 30 minutes at 120 °C. After cooling at room temperature, the mixture was diluted with DMSO and syringe filtered to remove any insoluble salts. The crude material was purified using reverse phase HPLC to afford the title compound (1.78 g, 60% yield) as an off-white powder. ES-MS $[M+1]^+$: 380.4. ^1H NMR (400 MHz, DMSO-d_6) δ 8.73 (t, J = 5.9 Hz, 1H), 8.68 (s, 1H), 7.41 (d, J = 8.4 Hz, 2H), 7.30 (d, J = 8.4 Hz, 2H), 4.95 (s, 1H), 4.75 (d, J = 5.8 Hz, 2H), 2.99 (s, 3H), 2.77 (s, 3H), 1.39 (s, 6H). ^{13}C NMR (101 MHz, DMSO) δ 161.9, 157.0, 156.5, 155.2, 152.9, 149.4, 136.5, 134.7, 127.4, 126.9 (2C), 124.6 (2C), 116.1, 70.6, 43.4, 32.0 (2C), 19.4, 13.7; HR-MS (Q-TOF, ES+) calc'd for $C_{20}H_{21}N_5OS$, 380.1540; found, 380.1541.

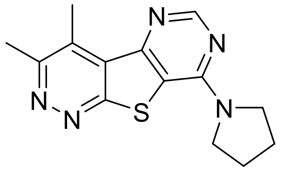

3,4-Dimethyl-8-(pyrrolidin-1-yl)pyrimido[4',5':4,5]thieno[2,3-*c*]pyridazine (15e, VU6006867). A solution of 8-chloro-3,4-dimethylpyrimido[4',5':4,5]thieno[2,3-*c*]pyridazine (40 mg, 0.16 mmol), pyrrolidine (30 µL, 0.36 mmol) and DIEA (58 µL, 0.58 mmol) in NMP (1.0 mL) was microwave irradiated for 15 minutes at 180 °C. After cooling at room temperature, the mixture was diluted with DMSO and syringe filtered to remove any insoluble salts. The crude material was purified using reverse phase HPLC to afford the title compound (5.6 mg, 12% yield). ES-MS $[M+1]^+$: 286.2. ^1H NMR (400 MHz, $CDCl_3$) δ 8.64 (s, 1H), 4.21 - 3.77 (m, 4H), 3.05 (s, 3H), 2.85 (s, 3H), 2.16 – 2.05 (m, 4H). ^{13}C NMR (101 MHz, $CDCl_3$) δ 162.5, 157.0, 156.5, 155.0, 154.4, 135.1, 127.9, 116.6, 48.2 (2C), 25.5 (2C), 20.1, 14.4. HR-MS (Q-TOF, ES+) calc'd for $C_{14}H_{15}N_5S$, 286.1121; found, 286.1122.

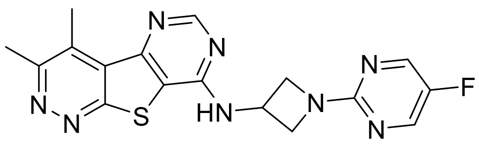

N-(1-(5-Fluoropyrimidin-2-yl)azetidin-3-yl)-3,4-dimethylpyrimido[4',5':4,5]thieno[2,3-*c*]pyridazin-8-amine (15k, VU6007458). A solution of intermediate 3,4-dimethylpyrimido[4',5':4,5]thieno[2,3-*c*]pyridazin-8-ol (20 mg, 0.086 mmol), 1-(5-fluoropyrimidin-2-yl)azetidin-3-amine (22 mg, 0.13 mmol), BOP (50 mg, 0.11 mmol), and DBU (20 mg, 0.13 mmol) in NMP (1.0 mL) was stirred at ambient temperature for 18 hours. The mixture was diluted with DMSO and syringe filtered to remove any insoluble salts. The crude material was purified using reverse phase HPLC to afford the title compound (5.4 mg, 16% yield). ES-MS $[M+1]^+$: 383.1. ^{1}H NMR (400 MHz, $CDCl_3$) δ 8.82 (s, 1H), 8.25 (d, J = 0.8 Hz, 2H), 5.45 (d, J = 6.5 Hz, 1H), 5.20 (qt, J = 7.1, 5.0 Hz, 1H), 4.64 (dd, J = 9.4, 7.3 Hz, 2H), 4.13 (dd, J = 9.5, 5.0 Hz, 2H), 3.08 (s, 3H), 2.88 (s, 3H). ^{13}C NMR (101 MHz, $CDCl_3$) δ 162.2, 160.1 (d, J_{C-F} = 1.7 Hz, 1C), 157.2, 156.8, 155.3, 154.7, 152.6 (d, J_{C-F} = 249.2 Hz, 1C), 145.7 (d, J_{C-F} = 21.8 Hz, 2C), 135.6, 128.6, 117.1, 58.2 (2C), 42.3, 20.0, 14.4. HR-MS (Q-TOF, ES+) calc'd for $C_{17}H_{15}FN_8S$, 383.1197; found, 383.1197.

General Procedure for the Preparation of Analogs **20** (Scheme 1).

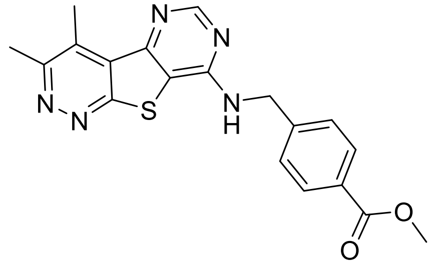

Methyl 4-(((3,4-dimethylpyrimido[4',5':4,5]thieno[2,3-*c*]pyridazin-8-yl)amino)methyl)benzoate. A solution of 8-chloro-3,4-dimethylpyrimido[4',5':4,5]thieno[2,3-*c*]pyridazine (251 mg, 1.0 mmol), methyl 4-(aminomethyl)benzoate hydrochloride (223 mg, 1.1 mmol) and DIEA (350 µL, 2.0 mmol) in NMP (5.0 mL) was microwave irradiated for 30 minutes at 120 °C. After cooling at room temperature, the mixture was syringe filtered to remove any insoluble salts and purified using reverse phase HPLC to afford the title compound (157 mg, 42% yield). ES-MS $[M+1]^+$: 380.1.

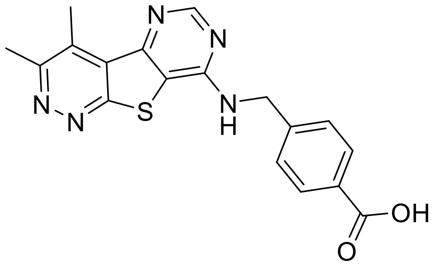

4-(((3,4-Dimethylpyrimido[4',5':4,5]thieno[2,3-*c*]pyridazin-8-yl)amino)methyl)benzoic acid (15w, VU6062615). To a solution of 4-(((3,4-dimethylpyrimido[4',5':4,5]thieno[2,3-*c*]pyridazin-8-yl)amino)methyl)benzoate (85 mg, 0.22 mmol) in THF/MeOH (2:1) (3.0 mL) was added an aqueous solution of 1*M* LiOH (2.4 mL, 2.4 mmol) and the mixture was stirred at 50 °C for 30 minutes. After cooling to 0 °C, the mixture was diluted with water and the pH was adjusted to 3-4 then concentrated. The material was carried forward without further purification. ES-MS $[M+1]^+$: 366.2. ^{1}H NMR (400 MHz, DMSO-d_6) δ 8.97 (t, *J* = 5.9 Hz, 1H), 8.68 (s, 1H), 7.90 (dt, *J* = 8.4, 2.1 Hz, 3H), 7.48 (dt, *J* = 8.3, 1.8 Hz, 2H), 4.85 (d, *J* = 5.9 Hz, 2H), 3.01 (s, 3H), 2.78 (s, 3H).

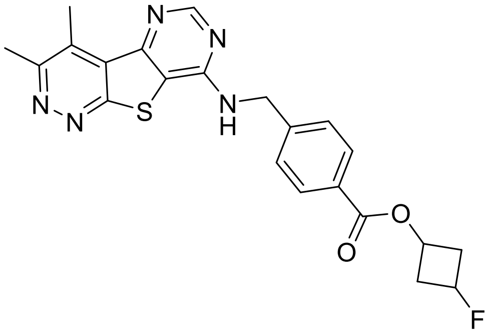

3-Fluorocyclobutyl 4-(((3,4-dimethylpyrimido[4',5':4,5]thieno[2,3-*c*]pyridazin-8-yl)amino)methyl)benzoate (20b, VU6009990). To a solution of intermediate 4-(((3,4-dimethylpyrimido[4',5':4,5]thieno[2,3-*c*]pyridazin-8-yl)amino)methyl)benzoic acid (30 mg, 0.082 mmol), HATU (90 mg, 0.24 mmol), and DIEA (98 µL, 0.56 mmol) in NMP (1.0 mL) was added 3-fluorocyclobutan-1-amine hydrochloride (52 mg, 0.41 mmol) and the mixture was stirred at ambient temperature for 20 minutes. The mixture was syringe filtered to remove any insoluble salts and purified using reverse phase HPLC to afford the title compound (1.1 mg, 3% yield). ES-MS $[M+1]^+$: 437.2. ^{1}H NMR (400 MHz, DMSO) (2.3:1 mixture of diastereomers) δ 8.80 (t, *J* = 5.9 Hz, 1H), 8.68 – 8.59 (m, 2H), 7.84 – 7.76 (m, 2H), 7.47 – 7.45 (m, 2H), 5.26 (dtt, *J* = 57.0, 6.4, 3.6 Hz, 0.3H minor); 4.9 (dp, *J* = 55.6, 6.8, 0.7H major), 4.83 (d, *J* = 5.8, 2H), 4.65 – 4.42 (m, 0.3H minor), 4.04 – 3.92 (m, 0.7H major), 2.98 (s, 3H), 2.77 (s, 3H), 2.76 – 2.68 (m, 1.6H minor + major), 2.53 – 2.38 (m, 1H minor + major), 2.34 – 2.20 (m, 1.4H minor + major); ^{13}C NMR (101 MHz, DMSO) (2.3:1 mixture of diastereomers) δ 165.81 (minor), 165.48 (major), 161.88, 157.11, 156.58, 155.20, 153.11, 142.41 (major), 142.39 (minor), 134.82, 133.03 (minor), 132.93 (major), 127.49, 127.40 (2C), 127.04 (2C), 116.20, 87.09 (d, *J* = 196.3 Hz, minor), 81.41 (d, *J* = 208.2 Hz, major), 40.71 (d, *J* = 8.4 Hz), 38.58 (d, *J* = 19.3 Hz), 37.45 (d, *J* = 21.5 Hz), 34.52 (d, *J* = 24.8 Hz), 19.44, 13.71. HR-MS (Q-TOF, ES+) calc'd for $C_{22}H_{21}FN_6OS$, 437.1554; found, 437.1553.

General Procedure for the Preparation of Analogs **37** (Scheme 2).

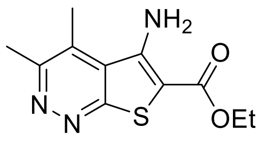

Ethyl 5-amino-3,4-dimethylthieno[2,3-*c*]pyridazine-6-carboxylate (28). To a 20 mL microwave vial was added commercially available starting material **21** (1.20 g, 7.16 mmol), potassium carbonate (1.98 g, 14.3 mmol), IPA (15 mL), and ethyl thioglycolate (**27**) (0.87 mL, 7.88 mmol). After 20 min at 105 °C in the microwave reactor, the reaction was cooled to room temperature and added to water (150 mL). The solids were filtered and washed with water (3x) to yield intermediate **28** as a green powder (1.42 g, 79% yield). ES-MS $[M+1]^+$: 252.2; ^1H NMR (400 MHz, $CDCl_3$) δ 6.18 (s, 2H), 4.41 (q, *J* = 7 Hz, 2H), 2.85 (s, 3H), 2.77 (s, 3H), 1.44 (t, *J* = 7.12 Hz, 3H).

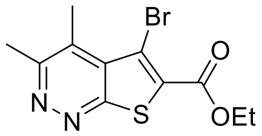

Ethyl 5-bromo-3,4-dimethylthieno[2,3-*c*]pyridazine-6-carboxylate (33). To a mixture of intermediate ethyl 5-amino-3,4-dimethylthieno[2,3-*c*]pyridazine-6-carboxylate (3.8 g, 15.1 mmol) and MeCN (25 mL) at 23 °C was slowly added via addition funnel over 20 minutes a solution of cupric bromide (1.69 g, 7.56 mmol) and *tert*-butyl nitrite (2.53 mL, 21.17 mmol) in MeCN (20 mL). After complete addition, the reaction stirred at 23 °C for 1.5 hours. The reaction was poured into 500 mL water and vigorously stirred. The suspension was vacuum filtered and washed with water (~1000 mL). The solids were isolated and dried by high vacuum to give title compound (3.12 g, 66% yield). Material was carried forward without further purification. ES-MS $[M+1]^+$: 316.0.

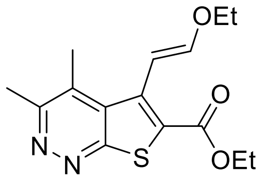

Ethyl (*E*)-5-(2-ethoxyvinyl)-3,4-dimethylthieno[2,3-*c*]pyridazine-6-carboxylate (35). A mixture of intermediate ethyl 5-bromo-3,4-dimethylthieno[2,3-*c*]pyridazine-6-carboxylate (12.3 g, 38.9 mmol), (*E*)-2-(2-ethoxyvinyl)-4,4,5,5-tetramethyl-1,3,2-dioxaborolane (8.66 mL, 40.9 mmol), and Cs_2CO_3 (38.1 g, 117 mmol) were added to a vessel followed by 1,4-dioxane (354 mL) and water (35.5 mL). The reaction mixture was degassed with bubbling of nitrogen through the solution for 15 minutes. Afterwards, Pd(dppf)Cl_2 (1.43 g, 1.95 mmol) was added and the reaction was heated to 85 °C for 16 hours. The mixture was cooled and additional Pd(dppf)Cl_2 (1.43 g, 1.95 mmol) and (*E*)-2-(2-ethoxyvinyl)-4,4,5,5-tetramethyl-1,3,2-dioxaborolane (8.66 mL, 40.9 mmol) were added. The mixture was stirred for an additional 72 hours at 85 °C then cooled to ambient temperature, filtered over a pad of Celite®, and washed with EtOAc. The filtrate was diluted with water and the organics were isolated. The aqueous

layer was further extracted with EtOAc (2x) then with $CHCl_3$/IPA (3:1) until the organic layer was no longer dark in color. The extractions were combined, dried ($MgSO_4$), filtered, and solvent was removed under vacuum to afford title compound which was carried forward without further purification. ES-MS $[M+1]^+$: 307.3.

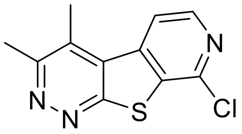

8-Chloro-3,4-dimethylpyrido[4',3':4,5]thieno[2,3-*c*]pyridazine (36). Step 1) A solution of ethyl (*E*)-5-(2-ethoxyvinyl)-3,4-dimethylthieno[2,3-*c*]pyridazine-6-carboxylate (627 mg, 2.05 mmol) in trifluoroacetic acid (10.8 mL, 140.3 mmol) was microwave irradiated at 120 °C for 1 hour. After cooling to ambient temperature, the reaction mixture was diluted with DCM and concentrated *in vacuo*. The crude sample was purified by normal-phase column chromatography on silica gel (0-40% EtOAc/DCM) to afford 3,4-dimethyl-8*H*-pyrano[4',3':4,5]thieno[2,3-*c*]pyridazin-8-one (368 mg, 51% yield) which was carried forward without further purification. ES-MS $[M+1]^+$: 233.2.

Step 2) To a reinforced pressure vessel containing 3,4-dimethyl-8*H*-pyrano[4',3':4,5]thieno[2,3-*c*]pyridazin-8-one (330 mg, 1.42 mmol) was added ammonium hydroxide (2.77 mL, 71.0 mmol). The vessel was capped and heated to 100 °C for 1.5 hours. The sample was transferred to a flask using DCM/MeOH and concentrated to dryness (azeotroped with methanol). The crude oil was sonicated in IPA and filtered to give 3,4-dimethylpyrido[4',3':4,5]thieno[2,3-*c*]pyridazin-8-ol (235 mg, 72% yield) which was used without further purification. ES-MS $[M+1]^+$: 232.2.

Step 3) To a solution of 3,4-dimethylpyrido[4,5]thieno[1,2-*c*]pyridazin-8-ol (249 mg, 1.08 mmol) was added phosphorous (V) oxychloride (10.0 mL, 108 mmol) and the mixture was microwave irradiation for 30 minutes at 150 °C. After cooling to ambient temperature, the sample was transferred to a flask and concentrated to dryness. The crude solid was basified with neat triethylamine until pH >8. The aqueous suspension was extracted with DCM (3x). The combined organics were passed through a phase separator, rinsed with DCM, and concentrated to give title compound (169 mg, 0.68 mmol). ES-MS $[M+1]^+$: 250.2.

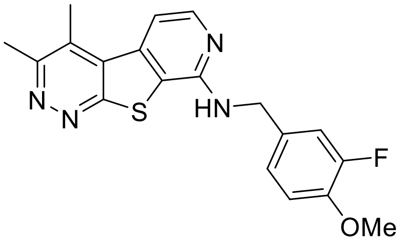

***N*-(3-Fluoro-4-methoxybenzyl)-3,4-dimethylpyrido[4',3':4,5]thieno[2,3-*c*]pyridazin-8-amine (37d, VU6009585).** In a vial were combined 8-chloro-3,4-dimethylpyrido[4',3':4,5]thieno[2,3-*c*]pyridazine (140 mg, 0.56

mmol), (3-fluoro-4-methoxyphenyl)methanamine (104 mg, 0.67 mmol), Cs_2CO_3 (0.06mL, 0.78mmol), $Pd_2(dba)_3$ (51 mg, 0.06 mmol), and Xantphos (65 mg, 0.11 mmol). The vial was capped and degassed (3x) followed by addition of 1,4-dioxane (2.8 mL). The vial was heated to 110 °C overnight then cooled to room temperature and diluted with water and $CHCl_3$/IPA (3:1). The organics were isolated and the aqueous layer was further extracted with $CHCl_3$/IPA (3:1) (3x). The organics were combined, passed through as phase separator, and concentrated. The crude sample was dissolved in DMSO and purified by reverse-phase HPLC (25-75 ACN/Water/0.1% TFA). The desired fractions were basified with saturated $NaHCO_3$ and the MeCN was removed. The aqueous layer was extracted with DCM (3x) and the combined organics were passed through a phase separator and concentrated. The free base was dissolved in DCM and 0.5 mL HCl in 1,4-dioxane (4M) was added. The sample was concentrated to afford title compound (103 mg, 46 % yield). ES-MS $[M+1]^+$: 369.3. ^1H NMR (400 MHz, DMSO-d_6) δ 8.11 (d, J = 6.3 Hz, 1H), 7.77 (d, J = 6.3 Hz, 1H), 7.34 (dd, J = 12.5, 2.1 Hz, 1H), 7.28 – 7.20 (m, 1H), 7.13 (t, J = 8.7 Hz, 1H), 4.80 (s, 2H), 3.80 (s, 3H), 2.85 (s, 3H), 2.84 (s, 3H). ^{13}C NMR (101 MHz, DMSO-d_6) δ 162.8, 156.0, 152.5, 150.1, 146.3, 146.2, 140.0, 135.1, 129.1, 123.9, 115.4, 115.2, 113.8, 113.7, 110.0, 56.0, 44.1, 19.7, 15.6. HR-MS (Q-TOF, ES+) calc'd for C19H17FN4OS, 369.1180; found, 369.1181.

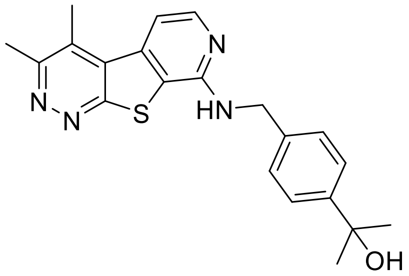

2-(4-(((3,4-Dimethylpyrido[4',3':4,5]thieno[2,3-*c*]pyridazin-8-yl)amino)methyl)phenyl)propan-2-ol (37e, VU6009795). Step 1) To a vial were combined intermediate 8-chloro-3,4-dimethylpyrido[4',3':4,5]thieno[2,3-*c*]pyridazine (100 mg, 0.4 mmol), methyl 4-(aminomethyl)benzoate hydrochloride (97 mg, 0.48 mmol), Cs_2CO_3 (184 mg 0.56 mmol), $Pd_2(dba)_3$ (37mg, 0.04 mmol), and Xantphos (46 mg, 0.08 mmol). The vessel was capped and degassed (3x) followed by addition of 1,4-dioxane (2.0 mL). The vial was heated to 110 °C overnight then cooled to room temperature and diluted with water and $CHCl_3$/IPA (3:1). The organics were isolated and the aqueous layer was further extracted with $CHCl_3$/IPA (3:1) (3x). The organics were combined, passed through as phase separator, and concentrated. The crude sample was dissolved in DMSO and purified by reverse-phase HPLC (10-50% ACN/Water/0.1% TFA). The desired fractions were basified with saturated $NaHCO_3$ and the MeCN was removed. The aqueous layer was extracted with DCM (3x) and the combined organics were passed through a phase separator and concentrated to give title compound (106 mg, 70% yield). ES-MS $[M+1]^+$: 379.4.

Step 2) To a suspension of methyl 4-(((3,4-dimethylpyrido[4',3':4,5]thieno[2,3-*c*]pyridazin-8-yl)amino)methyl)benzoate (106 mg, 0.28 mmol) in THF (2.8 mL) at ambient temperature was added

methylmagnesium bromide (470 µL, 1.4 mmol). The mixture was allowed to stir for 2 hours at ambient temperature after which the reaction was cooled to 0 °C and a saturated solution of NH_4Cl was added followed by dilution with water and EtOAc. The organic layer was isolated and the aqueous layer was extracted further with EtOAc (3x). The combined organics were dried ($MgSO_4$), filtered, and concentrated. The crude material was purified using reverse-phase HPLC (5-45% ACN/water/0.1% TFA). The desired fractions were basified with saturated aqueous $NaHCO_3$, the MeCN was removed, and the aqueous layer was extracted with DCM (3x). The combined organics were passed through a phase separator to give title compound (60 mg, 57% yield). ES-MS $[M+1]^+$: 379.2. ^{1}H NMR (400 MHz, DMSO-d_6) δ 8.17 (d, J = 5.6 Hz, 1H), 7.67 (t, J = 5.9 Hz, 1H), 7.58 (d, J = 5.7 Hz, 1H), 7.38 (d, J = 8.4 Hz, 2H), 7.29 (d, J = 8.3 Hz, 2H), 4.92 (s, 1H), 4.70 (d, J = 5.8 Hz, 2H), 2.81 (s, 3H), 2.79 (s, 3H), 1.38 (s, 6H). ^{13}C NMR (101 MHz, DMSO-d_6) δ 162.7, 155.7, 153.3, 148.9, 144.0, 138.4, 137.8, 133.6, 129.3, 126.7 (2C), 124.4 (2C), 118.8, 109.7, 70.5, 43.8, 32.0 (2C), 20.0, 15.5. HR-MS (Q-TOF, ES+) calc'd for $C_{21}H_{22}N_4OS$, 379.1587; found, 379.1588.

DMPK Methods

IV PK and plasma-brain level determination (PBL). **VU6008055**

In-life phase

Compounds were formulated as a solution in ethanol, PEG400, and saline (1:4:5 v/v, respectively) at a concentration of 1 mg/mL and administered as a single 0.2 mg/kg IV dose (1 mL/kg) to male, Sprague Dawley rats (n = 1; 342 gram body weights) via injection into a surgically-implanted jugular vein catheter. Blood samples were collected serially from a surgically implanted carotid artery catheter in each animal over multiple post-administration time points (0.033, 0.117, 0.25, 0.5, 1, 2, 4, 7, and 24 hours) into chilled, K2EDTA anticoagulant-fortified tubes and immediately placed on wet ice. The blood samples were then centrifuged (1700 rcf, 5 minutes, 4 °C) in order to obtain plasma samples, which were stored at -80 °C until analysis by LC-MS/MS.

For determination of the brain over plasma ratio (K_p), compounds were formulated in 8% ethanol, 32% PEG400 and 60% DMSO (v/v/v) and administered as a single 0.2 mg/kg IV dose (1 mL/kg) to male, Sprague Dawley rats (n = 1; 316 gram body weights) via injection into a surgically-implanted jugular vein catheter. At 15 minutes post dosing, blood sample was collected serially (i.e., terminally) into chilled, K2EDTA anticoagulant-fortified tube and immediately placed on wet ice. The blood sample was then centrifuged (1700 rcf, 5 minutes, 4 °C) to obtain plasma sample. At the same post-administration time point, whole brain sample was obtained by rapid dissection, rinsed with PBS, and immediately frozen in individual tissue collection box (dry ice). All brain and plasma samples were stored at -80 °C until analysis by LC-MS/MS.

Samples preparation for bioanalysis

Plasma samples from the in-life phase of the study were thawed at ambient temperature (benchtop), and then aliquots (20 µL per sample) were transferred to a 96-shallow-well (V-bottom) plate. Matrix-matched quality control (QC) samples and a standard curve of each compound (1 mg/mL DMSO stock solution) were prepared in blank rat plasma (K2EDTA-treated) or blank brain homogenate via serial dilution and transferred (20 µL each) to the plate along with multiple blank plasma and brain homogenate samples. Acetonitrile (120 µL) containing IS (10 nM carbamazepine) was added to each well of the plate to precipitate protein. The plate was then centrifuged (4000 rcf, 5 minutes, ambient temperature), and resulting supernatants (60 µL each) were transferred to a new 96-shallow-well (V-bottom) plate containing an equal volume (60 µL per well) of water (Milli-Q purified). The plate was then sealed in preparation for LC-MS/MS analysis.

Preparation of brain samples was identical to that of plasma samples except for the following modifications. While thawing, brains were weighed (inside their collection boxes using a universal empty collection box tare weight) and then subjected to mechanical homogenization (Mini-BeadBeater™, BioSpec Products, Inc., Bartlesville, OK) in the presence of zirconia/silica beads (1.0 mm) and extraction buffer (isopropanol:water, 7:3, v/v; 3 mL per sample, corrected for post-quantitation). Homogenized brain samples were then centrifuged (4000 rcf, 5 minutes, ambient temperature), and 5 µL of the supernatant was diluted in 15 µL of blank plasma for quantification of the analyte. The plasma standard curve and QCs were used for compounds quantitation in brain.

<u>Binding in plasma from rat and human.</u>

Determination of compounds' fraction unbound (f_u) in plasma from rat and human was conducted *in vitro* via equilibrium dialysis using HTDialysis membrane plates. Dialysis membranes (four paired strips per HTD assay) were hydrated as described by the manufacturer and inserted into the HTD plate, which was assembled and prepared for sample addition by the dispensing of blank buffer (DPBS, 100 µL/well) into the 'top half' of each membrane-split well. Each compound was diluted into plasma from each species (5 µM final concentration), which was aliquoted in triplicate to the 'bottom half' of the prepared HTD plate wells. The HTD plate was sealed and incubated for 6 hours at 37 °C. Following incubation, each well (both top and bottom halves) were transferred (20 µL) to the corresponding wells of a 96-shallow-well (V-bottom) plate. The daughter plates were then matrix-matched (buffer side wells received equal volume of plasma, and plasma side wells received equal volume of buffer), and extraction solution (120 µL; acetonitrile containing 50 nM carbamazepine as IS) was added to all wells of both daughter plates to precipitate protein and extract test article. The plates were then sealed and centrifuged (3500 rcf) for 10 minutes at ambient temperature. Supernatant (60 µL) from each well of the daughter plates was then transferred to the corresponding wells of new daughter plates (96-shallow-well, V bottom) containing water (Milli-Q, 60 µL/well), and the plates were sealed in preparation for LC-MS/MS analysis (see below).

f_u was calculated as (analyte to IS MS peak area ratio from Trans-buffer side) / (analyte to IS MS peak area ratio from Cis-plasma side). Mean values for each species were calculated from 3 replicates.

Binding in brain homogenate from rat.

Determination of fraction unbound (f_u) in brain homogenate from rat was conducted using the same methodology and procedure than described for plasma protein binding assay with the following modifications: 1) a final compound concentration of 1 μM was used, 2) naïve rat brains were homogenized in DPBS (1:3 composition of brain: DPBS, w/w) using a Mini-Bead Beater™ machine in order to obtain brain homogenate.

The diluted fraction unbound (f_{u2}) in brain was calculated as (analyte to IS MS peak area ratio from Trans-buffer side) / (analyte to IS MS peak area ratio from Cis-brain homogenate side). Undiluted fraction unbound for the brain was calculated using the following equation:

$$f_u = \frac{1/4}{\left(\left(\frac{1}{f_{u2}}\right) - 1\right) + 1/4}$$

Mean values for each species were calculated from 3 replicates.

Intrinsic Clearance in Rat and Human Liver Microsomes

The *in vitro* intrinsic clearance (CL_{int}) was investigated in commercially obtained hepatic microsomes from rat and human donors using the substrate depletion (i.e., loss-of-parent vs. time, or $t_{1/2}$ method) approach with analyte detection via liquid chromatography-tandem mass spectrometry (LC-MS/MS). For each species, mean %parent remaining values at each time point were calculated from replicates raw data (analyte:IS peak area ratios) and used to determine *in vitro* $t_{1/2}$ and CL_{int}.

Experiments were carried out using a robot-assisted (TECAN model Evo 200). Compound was incubated (1 μM final concentration) in buffer (100 mM potassium phosphate pH 7.4 with 3 mM $MgCl_2$) containing hepatic microsomes (0.5 mg/mL final concentration) from multiple species, discretely, at 37 °C under constant orbital shaking. After 5 minutes (pre-incubation), reactions were initiated by addition of nicotinamide adenine dinucleotide phosphate (NADPH, 1 mM final concentration). At selected time intervals (0, 3, 7, 15, 25, and 45 minutes) post-addition of NADPH, aliquots (50 μL) were taken and placed into a 96-shallow-well plate containing ice cold acetonitrile (150 μL) with carbamazepine (IS, 50 nM). The plates were then centrifuged (3000 rcf at 4 °C) for 10 minutes. The supernatants were transferred to a new 96-shallow-well daughter plate and diluted (1:1 v/v) with water (Milli-Q filtered). The plates were then sealed in preparation for LC-MS/MS analysis (see below).

Raw LC-MS/MS peak area data generated from the assay samples were used to construct natural log-transformed %parent remaining vs. time plots (using t = 0 minute post-NADPH addition sample data as starting point set to 100%). *In vitro* compound half-life ($t_{1/2}$) values were obtained using the following equation:

$$t_{1/2} = \frac{\ln(2)}{k}$$

Where k is the slope from linear regression analysis of the natural log-transformed data (using means from all replicates at each time point). Resulting $t_{1/2}$ values were then used to calculate hepatic CL_{int} values according to the following equation and with the use of species-specific scale-up factors for liver weight (grams) per total body weight (kg):

$$CL_{int} = \frac{[illegible]}{[illegible]} \times \frac{[illegible]}{[illegible]} \times \frac{[illegible]}{[illegible]} \times \frac{[illegible]}{[illegible]}$$

[a]Scale-up factors used are 45 (rat) and 20 (human).[1]

Predicted hepatic clearance (CL_{hep}) was calculated using the following equation:

$$CL_{hep} = \frac{Q_h * CL_{int}}{Q_h + CL_{int}}$$

Q_h represents hepatic blood flow (mL/min/kg): 21 for human, 70 for rat, and 90 for mouse.

<u>LC-MS/MS Analysis</u>

Prepared samples were injected (10 µL each) onto an AB Sciex Triple Quad 4500 mass spectrometer system with an Agilent 1260 Infinity II pump and autosampler. Mass spectrometer conditions are described in **Table S1.** Quantitation of compounds was performed via AB Sciex Multiquant software using the raw analyte:IS peak area ratios. The typical detection range was 0.5 ng/mL to ≥ 5,000 ng/mL utilizing a quadratic equation regression with 1/x2 weighting.

Correction for dilution of all brain samples (in extraction buffer and subsequently in blank plasma, as previously described) was performed post-quantitation. The corrections for dilution in extraction buffer employed correction factors specific to each brain weight (not shown).

Table S1. LC-MS/MS Conditions*

Injection volume	10 µL	
Mobile phase A	0.5% Formic Acid in Water	
Mobile phase B	0.5% Formic Acid in Acetonitrile	
Flowrate	0.5 mL/min	
Gradient	Time	% Mobile Phase B
	0.0	5
	0.2	5
	0.8	95
	1.5	95
	1.7	5
	2.7	Stop
Column	Fortis C18 (50 x 3.0 mm, 3 µm)	
Data collection and analysis software/version	Analyst v. 1.7.1	
Ionization mode	Positive Electrospray	

Curtain gas (psi)	40
GS1 (psi)	40
GS2 (psi)	40
Capillary voltage (V)	5500
Source TurboIonSpray® temp. (°C)	500

Lead Profiling Screen to Assess Off-Target Liabilities

Table S2. Lead Profiling Screen – Eurofins Panlabs for **VU6008055.**

Assay Name	Species	% inh at 10 µM
Adenosine A_1	hum	45
Adenosine A_{2A}	hum	50
Adenosine A_3	hum	17
Adrenergic α_{1A}	rat	19
Adrenergic α_{1B}	rat	9
Adrenergic α_{1D}	hum	-4
Adrenergic α_{2A}	hum	-0
Adrenergic β_1	hum	12
Adrenergic β_2	hum	6
Androgen (Testosterone)	hum	3
Bradykinin B_1	hum	-10
Bradykinin B_2	hum	-7
Calcium Channel L-Type, Benzothiazepine	rat	19
Calcium Channel L-Type, Dihydropyridine	rat	-9
Calcium Channel N-Type	rat	15
Cannabinoid CB_1	hum	4
Dopamine D_1	hum	6
Dopamine D_{2S}	hum	11
Dopamine D_3	hum	1
Dopamine $D_{4.2}$	hum	13
Endothelin ET_A	hum	7
Endothelin ET_B	hum	8
Epidermal Growth Factor (EGF)	hum	2
Estrogen ER_α	hum	5
$GABA_A$, Flunitrazepam, Central	rat	13
$GABA_A$, Muscimol, Central	rat	5
$GABA_{B1A}$	human	-3
Glucocorticoid	hum	16
Glutamate, Kainate	rat	26
Glutamate, NMDA, Agonism	rat	6

Glutamate, NMDA, Glycine	rat	2
Glutamate, NMDA, Phencyclidine	rat	-12
Histamine H_1	hum	5
Histamine H_2	hum	-9
Histamine H_3	hum	21
Imidazoline I_2, Central	rat	18
Interleukin IL-1	mouse	18
Leukotriene, Cysteinyl $CysLT_1$	hum	31
Melatonin MT_1	hum	14
Muscarinic M_1	hum	2
Muscarinic M_2	hum	9
Muscarinic M_3	hum	3
Neuropeptide Y Y_1	hum	13
Neuropeptide Y Y_2	hum	5
Nicotinic Acetylcholine	hum	-5
Nicotinic Acetylcholine α1, Bungarotoxin	hum	-2
Opiate δ_1 (OP1, DOP)	hum	5
Opiate κ (OP2, KOP)	hum	2
Opiate μ (OP3, MOP)	hum	4
Phorbol Ester	mouse	-1
Platelet Activating Factor (PAF)	hum	4
Potassium Channel [K_{ATP}]	ham	-10
Potassium Channel hERG	hum	2
Prostanoid EP_4	hum	22
Purinergic P2X	rabbit	18
Purinergic P2Y	rat	0
Rolipram	rat	8
Serotonin (5-Hydroxytryptamine) $5\text{-}HT_{1A}$	hum	-8
Serotonin (5-Hydroxytryptamine) $5\text{-}HT_{2B}$	hum	-6
Serotonin (5-Hydroxytryptamine) $5\text{-}HT_3$	hum	-3
Sigma σ_1	hum	-1
Sodium Channel, Site 2	rat	23
Tachykinin NK_1	hum	-5
Thyroid Hormone	rat	-4
Transporter, Dopamine (DAT)	hum	11
Transporter, GABA	rat	-3
Transporter, Norepinephrine (NET)	hum	36
Transporter, Serotonin (5-Hydroxytryptamine) (SERT)	hum	-10

Evaluation of Potential Cardiac Risks

Table S3. Cardiac Ion Channel Panel – Charles River Laboratories for **VU6008055**

Ion Channel	Concentration (μM)	Mean % Inhibition	Standard Deviation	Standard Error	n
hCav1.2	10	12.4	2.5	1.4	3
hCav3.2	10	8.3	4.9	2.5	4
hHCN2	10	3.9	4.6	2.7	3
hERG	10	4.2	4.0	2.3	3
hKv1.5	10	0.3	12	6.9	3
hKvLQT1/mink	10	1.5	10.6	5.3	4
hKv1.3	10	-1.0	4.1	2	4
hNav1.5 (Tonic)	10	3.0	4.0	2.3	3
hNav1.5 (Phasic)	10	2.8	1.7	1.0	3

The *in vitro* effects of **VU6008055** were evaluated at room temperature using the QPatch HT® (Sophion Bioscience A/S, Denmark), an automatic parallel patch clamp system. **VU6008055** was evaluated at a concentration of 10 μM and tested in at least 3 cells ($n \geq 3$). The duration of exposure to each test article concentration was at least 3 minutes.

In-vitro Determination of Brain Penetration Potential

Assessment of *in vitro* permeability and active transport in MDR1-transfected MDCKII cell line.

The apparent permeability of **VU6008055** was investigated through a monolayer of MDR1 (human multi drug resistance gene or P-gp) transfected MDCKII (Madin Darby canine kidney) cell line grown on permeable filters. The ratio between the basolateral-to-apical (B→A) and the apical-to-basolateral (A→B) apparent permeability coefficients (P_{app}) with and without P-gp inhibitor (elacridar, 10 μM) was used to assess the potential involvement of P-gp in the transport of **VU6008055** (0.5 μM) through the cell layer. Permeability and efflux ratio data for **VU6008055** are shown in **Table S4.** [illegible]

VU6008055 total recovery in the assay was >80% in both directions (A→B) and (B→A). Metoprolol (high permeability reference compound), fenoterol (low permeability reference compound), and digoxin (P-gp substrate reference compound) were all within acceptable limits.

Table S4. Permeability coefficient (P_{app}) in MDCKII-MDR1 cells (cm/s × 10^{-6}) and estimated efflux ratio of **VU6008055**. Data shown as mean values and [range] (n=3, N=2).

Compound	Conc. (µM)	Direction	P_{app} (cm/s x 10^{-6})	Ratio B-A/A-B
**Without a P-gp inhibitor**				
VU6008055	0.5	A-B	6.4 [5.4-7.5]	0.83 [0.55-1.1]
VU6008055	0.5	B-A	5.0 [4.1-6.0]	
**With a P-gp inhibitor**				
VU6008055	0.5	A-B	8.0 [6.1-10]	1.1 [0.78-1.4]
VU6008055	0.5	B-A	8.2 [7.8-8.6]	

Based on P_{app} from MDCKII **VU6008055** is classified as having moderate permeability. A ratio between permeabilities (B-A/A-B) of 0.8 without P-gp inhibitor and 1.1 with P-gp inhibitor suggests that **VU6008055** is not a substrate for the efflux transporter P-gp.

<u>Assessment of *in vitro* permeability and active transport in BCRP-transfected MDCKII cell line.</u>

The apparent permeability of **VU6008055** was investigated through a monolayer of BCRP (Breast Cancer Resistance Protein) transfected MDCKII (Madin Darby canine kidney) cell line grown on permeable filters. The ratio between the basolateral-to-apical (B→A) and the apical-to-basolateral (A→B) apparent permeability coefficients (P_{app}) with and without BCRP inhibitor (KO 143, 1 µM) was used to assess the potential involvement of BCRP in the transport of **VU6008055** (1 µM) through the cell layer. Permeability and efflux ratio data for **VU6008055** are shown in

[illegible]. Metoprolol (high permeability reference compound), atenolol (low permeability reference compound), and prazosin (BCRP substrate reference compound) were all within acceptable limits. **VU6008055** total recovery in the assay was low (35-71%) in both directions (A→B) and (B→A). This low recovery might be due to unspecific binding as the assay was performed without addition of BSA (bovine serum albumin) to the incubation buffer.

Table S5. Permeability coefficient (P_{app}) in MDCKII-BCRP cells (cm/s × 10^{-6}) and estimated efflux ratio of **VU6008055**. Data shown as mean values and [range] (n=3, N=2).

Compound	Conc. (µM)	Direction	P_{app} (cm/s x 10^{-6})	Ratio B-A/A-B
**Without a BCRP inhibitor**				
VU6008055	1	A-B	2.3 [1.7-3.0]	7.5 [4.7-10]
VU6008055	1	B-A	16 [14-17]	
**With a BCRP inhibitor**				
VU6008055	1	A-B	8.2 [7.6-8.8]	1.3 [1.1-1.4]
VU6008055	1	B-A	10 [10-10]	

In line with results obtained in MDR1-MDCKII assay, the BCRP-MDCKII P_{app} A-B value indicates that **VU6008055** has moderate permeability. A ratio between permeabilities (B-A/A-B) of 7.5 without BCRP inhibitor and 1.3 with BCRP inhibitor suggests that **VU6008055** is a substrate of the human efflux transporter BCRP.

Metabolic Stability

Metabolic stability in liver microsomes and hepatocytes.

The metabolic stability of **VU6008055** was investigated in microsomes and cryopreserved hepatocytes from human, Sprague Dawley rat, Beagle dog, Cynomolgus monkey and Göttingen minipig (only hepatocytes) at 0.1, 0.3 and 1 µM incubation concentrations (**Table**).

VU6008055 has low intrinsic clearance in human, rat and minipig hepatocytes (and human and rat liver microsomes), moderate intrinsic clearance in monkey hepatocytes (and liver microsomes) and high intrinsic clearance in dog hepatocytes. Human intrinsic clearance in hepatocytes could not be determined as > 85% of the compound was remaining after end of incubation.

Monkey intrinsic clearance in hepatocytes was lower than in microsomes but as hepatocyte data is considered more physiologically relevant and was therefore used for IVIVE and PBPK predictions.

Table S6. VU6008055 Intrinsic clearance (CL_{hep}) of a 70 kg human, a 250 g Sprague Dawley rat, a 10 kg Beagle dog, a 4 kg Cynomolgus monkey and a 14 kg Göttingen minipig. Hepatocytes (mean ± SEM, n=3, N=3) and microsomes (mean ± SD, n=1, N=3).

	**Conc. (µM)**	**CL_{hep} (mL/min/kg)**				
		Human	Rat	Dog	Monkey	Minipig*
**Microsomes**	0.1	5.17 ± 0.20	35.8 ± 0.1	23.7 ± 0.3	30.0 ± 0.9	-
**Hepatocytes**		<5.17	25.2 ± 0.6	38.2 ± 1.5	23.5 ± 0.6	14.8 ± 0.02
**Microsomes**	0.3	6.17 ± 0.11	28.8 ± 0.3	19.3 ± 0.1	29.7 ± 0.5	-
**Hepatocytes**		<5.17	21.8 ± 0.5	38.8 ± 2.1	22.0 ± 0.3	13.7 ± 0.2
**Microsomes**	1	5.17 ± 0.10	25.2 ± 0.3	15.5 ± 0.2	29.0 ± 0.4	-
**Hepatocytes**		<5.17	25.2 ± 0.4	38.3 ± 2.0	23.5 ± 0.6	14.3 ± 0.5
**LBF**		**21.7**	**80.0**	**55.0**	**43.3**	**36.7**

*Mean ± SD, n=3, N=1

Positive controls in liver microsomes were Omaprazole, Diclofenac and Propafenone, and in hepatocytes Phthalazine, Propanolol, Diclofenac and Verapamil. All positive controls were within acceptance range. The lowest limit of quantification values are based on 15% of metabolism after 60 min of incubation. If >85% of compound is remaining at the end of incubation, CL_{int} is considered to be below the lower limit of quantification (LLOQ).

Metabolic stability in intestinal microsomes.

The metabolic stability of **VU6008055** was investigated in intestinal microsomes from human, Sprague Dawley rat, Beagle dog, and Cynomolgus monkey. **VU6008055** was incubated at 0.5 µM incubation concentration for 60 min and in the presence of co-factors (NADPH and UDPGA, respectively) and Alamethicin for enhancing phase II metabolism.

VU6008055 was stable in human, rat, dog and monkey intestinal microsomes with more than 90% test compound remaining in all species after 60 min incubation. Testosterone and 7-OH-coumarin were used as positive controls in all species and were performing as expected according to historical data.

Evaluation of phase 1 and phase 2 metabolism.

The metabolic stability of **VU6008055** was investigated in liver microsomes from human and Cynomolgus monkey. **VU6008055** was incubated at 1 µM incubation concentration and 0.5 mg/mL protein concentration for 60 min and in the presence of co-factors (NADPH and UDPGA) and Alamethicin for enhancing phase II metabolism.

VU6008055 data is presented in **Table S**. The lowest limit of quantification values is based on 15% of metabolism after 60 min of incubation. If > 85% of compound is remaining at the end of incubation, CL_{int} is considered to be below LLOQ. Positive controls for phase I metabolism were omeprazole, diclofenac and propafenone and for phase II metabolism were 7-Hydroxycoumarin. All controls show activity of both phase I and II metabolism.

Table S7. Liver microsomal clearance (CL_{hep}) of a 70 kg human and a 4 kg Cynomolgus monkey with and without NADPH and UDPGA (mean, n=2, 1 occasion).

**Compound (1µM)**	**Incubation condition**	**Human**		**Monkey**	
		CL_{hep} (mL/min/kg)	% Remaining T=60	CL_{hep} (mL/min/kg)	% Remaining T=60
**VU6008055**	With NADPH	<4.70	94	28.7	17
	With NADPH and UDPGA	<4.70	91	26.3	25
	With UDPGA	<4.70	110	9.80	80

Cytochrome P450 Inhibition Studies

In vitro tests were performed in order to assess the direct or time-dependent inhibitory (TDI) potential of **VU6008055** towards cytochrome P450 enzymes in pooled human liver microsomes. For direct inhibition, substrate and inhibitor (7 concentrations) were incubated simultaneously with the enzymes (Error! Reference source not found.). **VU6008055** showed no direct inhibition on CYP1A2, CYP2B6, CYP2D6 and CYP3A4, while showing a reversible inhibition of CYP2C8, CYP2C9 and CYP2C19 (39%, 49% and 35% decrease in formation of metabolite respectively, at the highest concentration).

Table S8. The direct inhibitory potential (IC_{50}, µM) of **VU6008055** towards seven cytochrome P450 enzymes (mean ± SD, n=3, N=1).

	**CYP 1A2**	**CYP 2B6**	**CYP 2C8**	**CYP 2C9**	**CYP 2C19**	**CYP 2D6**	**CYP 3A4-M**	**CYP 3A4-T**
				**VU6008055**				
IC_{50} (µM)	>15	>15	>15	>15	>15	>15	>15	>15
				**Positive controls**				
	α-Naph-thoflavone	Tamoxifen	Quercetin	Sulfaphenazole	Modafinil	Quinidine	Ketoconazole	Ketoconazole
				Passed				

Table S9. Time-dependent inhibition (IC_{50} fold change) of VU6008055 towards seven cytochrome P450 enzymes (n=3, N=1).

	**CYP 1A2**	**CYP 2B6**	**CYP 2C8**	**CYP 2C9**	**CYP 2C19**	**CYP 2D6**	**CYP 3A4-M**	**CYP 3A4-T**
				**VU6008055**				
IC_{50} (μM) (-) NADPH	>15	>15	>15	>15	>15	>15	>15	>15
IC_{50} (μM) (+) NADPH	>15	>15	>15	>15	>15	>15	>15	>15
IC_{50} fold change (μM)	NA	NA	NA	NA	NA	NA	NA	NA
				**Positive Controls**				
	Furafylline	Thio-TEPA	GemfibrozilGlucuronide	Tienilic Acid	S-fluoxetine	Paroxetine	Mifepristone	Mifepristone
				Passed				

NA: Not Applicable

The time-dependent inhibition potential of **VU6008055** was investigated by comparing its IC_{50} after 30 min pre-incubation with pooled human liver microsomes with NADPH to the ones obtained without NADPH (

Te). The test compound was assessed in triplicates. **VU6008055** did not exhibit a positive signal for time-dependent (NADPH-independent) nor time- and NADPH-dependent inhibition of any CYP tested.

Cytochrome P450 Induction Studies

The effect of **VU6008055** as a potential inducer of CYP1A2, CYP2B6, and CYP3A4 was assessed in cryopreserved human hepatocytes from 3 individual donors. The hepatocytes were plated on a collagen-coated plate and maintained in a cell culture incubator kept at 37 °C for 4 hours followed by a 0.25 mg/mL Matrigel overlay where after the hepatocytes were incubated for 24 hours before treatment with **VU6008055** (7 concentrations ranging between 0.1-100 µM) for additional 3 days (**Figure S1**). The medium was changed daily and at the end of incubation the culture medium containing **VU6008055** was removed, and the metabolism of selective substrates for CYP1A2, CYP2B6 and CYP3A4 was assessed. In addition, CYP induction was evaluated by gene expression measurement (mRNA). Only 5 test concentrations (0.1, 0.3, 1, 3, 10 µM) were used to assess the induction potential of **VU6008055**, as the kinetic solubility was measured to be 15 µM in PBS buffer.

Figure S1. Effects of **VU6008055** on fold induction in gene expression of CYP1A2, CYP2B, & CYP3A4 in cryopreserved human hepatocytes.

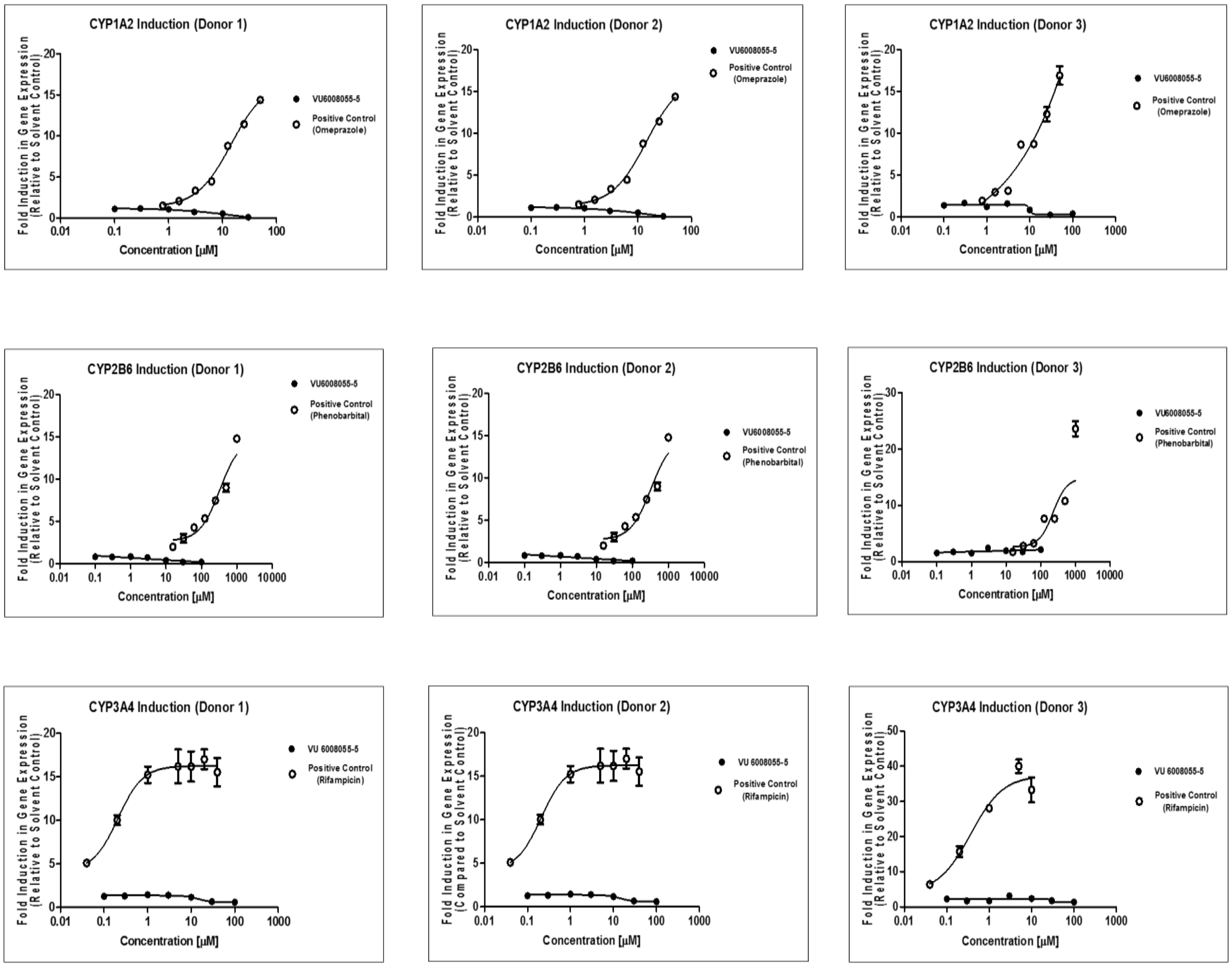

Table S10. Effects of **VU6008055** on CYP1A2, CYP2B6 and CYP3A4 gene expression in human hepatocytes (mean ± SD fold increase over solvent controls, n=3, N=1).

			**Enzyme activity**		
			**CYP1A2**	**CYP2B6**	**CYP3A4**
**Compound**	**Conc. (µM)**	**Donor #**	**Fold of DMSO control (% of positive control)**		
**VU6008055**	0.1	1	0.96 ± 0.08	0.97 ± 0.10	0.88 ± 0.03
		2	1.12 ± 0.09	0.86 ± 0.13	1.28 ± 0.10
		3	1.41 ± 0.23	1.54 ± 0.20	2.27 ± 0.50
	0.3	1	1.37 ± 0.36	1.36 ± 0.46	1.53 ± 0.80
		2	1.18 ± 0.08	0.82 ± 0.13	1.32 ± 0.14
		3	1.69 ± 0.22	1.73 ± 0.33	1.73 ± 0.32
	1	1	1.22 ± 0.05	1.45 ± 0.35	1.40 ± 0.75
		2	1.08 ± 0.04	0.88 ± 0.14	1.46 ± 0.05
		3	1.24 ± 0.05	1.51 ± 0.04	1.75 ± 0.17
	3	1	1.11 ± 0.06	1.41 ± 0.32	1.93 ± 0.50
		2	0.75 ± 0.06	0.75 ± 0.05	1.41 ± 0.13
		3	1.63 ± 0.18	2.43 ± 0.09	3.16 ± 0.26
	10	1	0.75 ± 0.07	1.95 ± 0.34	3.13 ± 0.31
		2	0.54 ± 0.03	0.43 ± 0.03	1.17 ± 0.05
		3	0.87 ± 0.05	1.93 ± 0.07	2.41 ± 0.05

EC_{50} values were not applicable due to lack of concentration-based response or maximum induction < 2-fold.

The result in Error! Reference source not found. indicates that **VU6008055** has the potential to induce CYP2B6 and CYP3A4 from a concentration of 3 µM. Omeprazole, phenobarbital and rifampicin, were used as control inducer compounds for CYP1A2, CYP2B6 and CYP3A4, respectively. Positive control induction expressed as fold of DMSO control were all above 7-fold.

These data were verified by a reporter gene activation study of human AhR, CAR and PXR (corresponding to activation of CYP1A, CYP2B and CYP3A respectively) where activation was detected in PXR at 4 µM (2.0-fold of DMSO control) and a tendency to activation of CAR at 10 µM (1.8-fold of DMSO control).

Fraction of CYP-mediated metabolism ($f_{m,CYP}$)

The metabolism of **VU6008055** was investigated in human, rat, dog and monkey hepatocytes. **VU6008055** was incubated at a test concentration of 0.3 µM in cryopreserved hepatocytes (1.0 x 106 cells/mL) at 37 °C for 4 hours and percent remaining was measured at 6 different timepoints. The study was performed with and without the addition of ABT (1-aminobenzotriazole) as a pan-CYP inhibitor to estimate the fraction of CYP-mediated metabolism ($f_{m,CYP}$) (**Figure S2**).

The fraction of CYP-mediated metabolism for **VU6008055** was determined to be 49%, 87%, 93% and 44% for human, Beagle dog, Sprague Dawley rat and Cynomolgus monkey respectively. The fractions of CYP-mediated metabolism in human hepatocytes of approximately 50% indicate a substantial contribution of non-CYP enzymes to the metabolic pathways for **VU6008055**.

Figure S2. Metabolic Stability of **VU6008055** in Cryopreserved Hepatocytes,

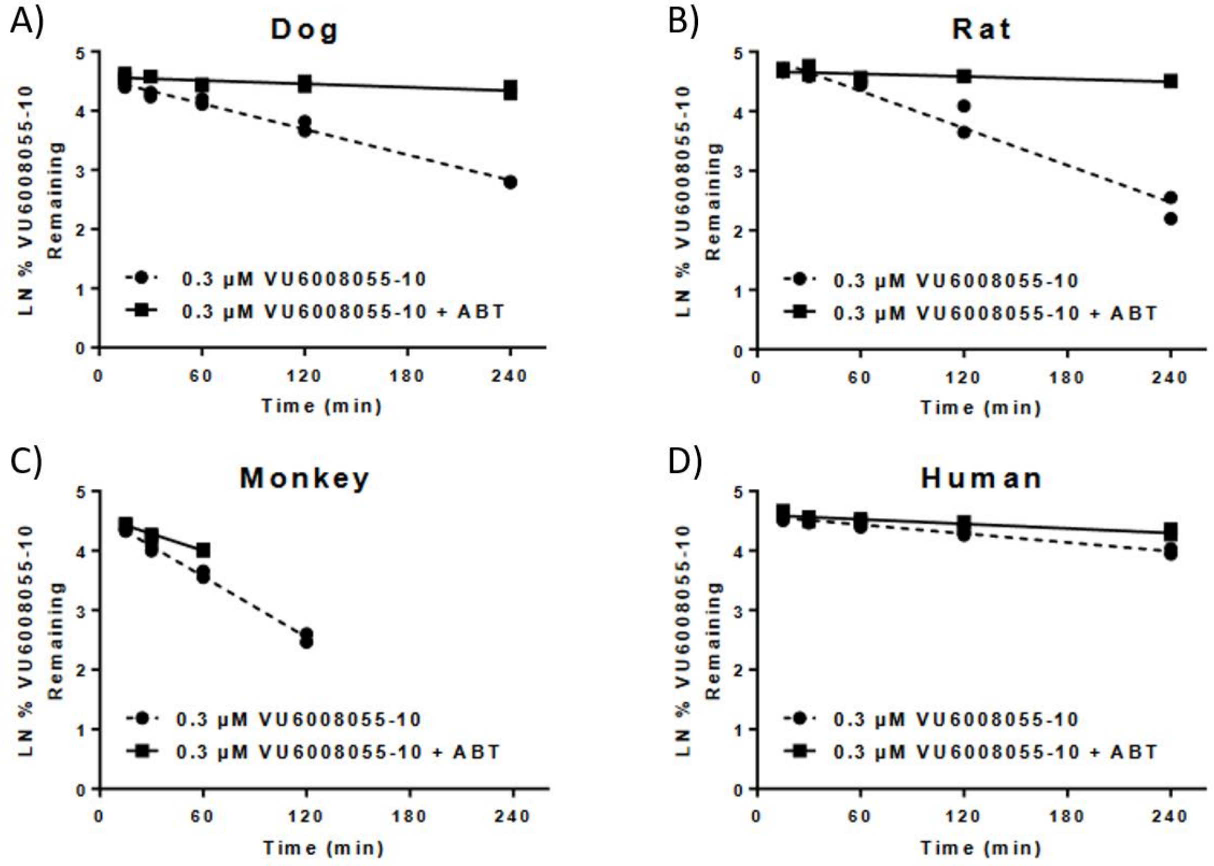

Assessment of involvement of aldehyde oxidase in metabolism

The involvement of aldehyde oxidase (AO) in the metabolism of **VU6008055** was assessed after incubation at 1 μM in human and monkey liver S9 mix (S9, 1.25 mg of protein/mL). The incubations were carried out in triplicates at six different time-points (0-90 min) in presence and absence of hydralazine and raloxifene (reference AO inhibitors). **VU6008055** was stable in both human and monkey liver S9 with 100% test compound remaining after 90 min incubation. Carbazeran (5 μM) was used as positive control and showed 100% turnover in both species in the absence of inhibitor.

Glutathione trapping.

Potential to form glutathione conjugates was investigated using a LC-HRMS technique through the GSH trapping assay in human liver microsomes. No glutathione conjugates were observed for **VU6008055** following 3.0 hours of incubation.

Figure S3. Extracted Ion Chromatogram of **VU6008055** in Human Liver Microsomes with Glutathione.

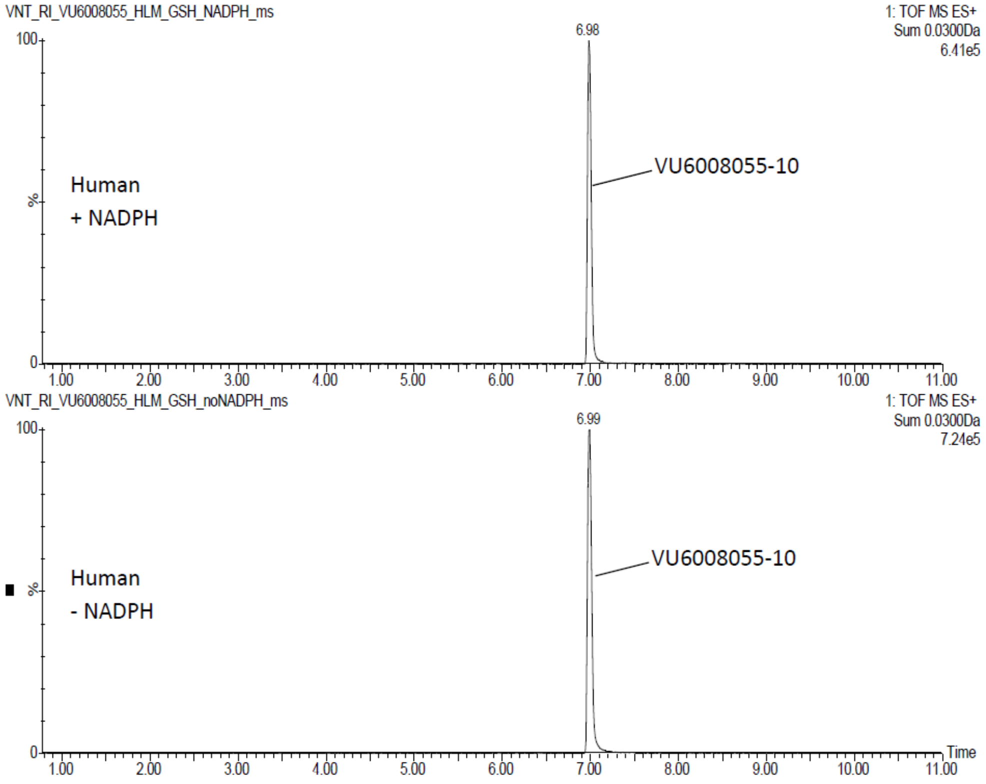

No glutathione or glutathione related conjugates were observed for **VU6008055** in HLM.

Multi-species Hepatocyte MetID

Qualitative metabolite profiling and tentative metabolite identification was performed in human, rat, dog, monkey and minipig hepatocytes. **VU6008055** (not radiolabeled) was incubated at a test concentration of 10 µM in cryopreserved hepatocytes (0.5 x 106 cells/mL) at 37 °C for 4 hours. T0 and T4 samples were quenched with an equal amount of acetonitrile and the supernatant were analyzed using accurate mass LC-MS and with UV detection.

VU6008055 incubations in human, rat, dog, monkey and minipig hepatocytes produced a total of 19 metabolites (**Table S11**). The primary metabolic pathways observed were direct glucuronidation or oxidation with and without additional glucuronide conjugation. Overall, the metabolic profile for monkey and minipig were similar to the human profile. The presence of hydroxylation and glucuronidation

metabolites in human hepatocytes indicate that **VU6008055** is likely to be metabolized via multiple metabolic pathways.

Table S11. Qualitative metabolite profiling in human, rat, dog, monkey and minipig hepatocytes

Peak ID	Tentative Metabolite Identification	*m/z*	Rt (min)	Human M+F	Rat M	Dog M	Monkey M	Minipig M
**VU6008055**	Parent (P)	380	7.08	X	X	X	X	X
MT1	P + O	396	6.68	X	X	X	X	X
MT2	P + O	396	5.89	X	X	X	X	X
MT3	P + O	396	5.76		X	X		X
MT4	P + O	396	5.73	X			X	X
MT5	P + glucuronide	556	4.74	X	X	X	X	X
MT6	P + O + glucuronide	572	4.35	X	X	X	X	X
MT7	P + O + glucuronide	572	4.00	X		X	X	
MT8	P + O + glucuronide	572	3.99	X	X		X	X
MT9	P + O + glucuronide	572	3.90	X	X	X	X	X
MT10	P + O + glucuronide	572	3.58	X		X	X	X

P = Parent, O = Oxidation, H = Hydroxylation

MPH = Minipig hepatocytes, RH = Rat hepatocytes, DH: Dog hepatocytes, HH: Human hepatocytes, PH: Monkey (Primate) hepatocytes. Retention times were obtained from the extracted ion chromatograms.

Table S12. Percent MS total peak areas of **VU6008055** and metabolites in each species.

<table>
<tr><th rowspan="2">Peak ID</th><th rowspan="2">Tentative Metabolite Identification</th><th colspan="5">% Total Peak Area</th></tr>
<tr><th>Rat</th><th>Dog</th><th>Monkey</th><th>Minipig</th><th>Human</th></tr>
<tr><td>VU6008055</td><td>Parent (P)</td><td>41.4</td><td>71.6</td><td>23.5</td><td>18.8</td><td>59.1</td></tr>
<tr><td>MT1</td><td>P + O</td><td>0.17</td><td>0.07</td><td>0.14</td><td>0.1</td><td>0.11</td></tr>
<tr><td>MT2</td><td>P + O</td><td>14.3</td><td>5.82</td><td>6.77</td><td>10.8</td><td>1.36</td></tr>
<tr><td>MT3</td><td>P + O</td><td>9.57</td><td>22</td><td>ND</td><td rowspan="2">14</td><td>ND</td></tr>
<tr><td>MT4</td><td>P + O</td><td>ND</td><td>ND</td><td>3.22</td><td>7.89</td></tr>
<tr><td>MT5</td><td>P + glucuronide</td><td>33.7</td><td>0.47</td><td>48.9</td><td>46.2</td><td>29.6</td></tr>
<tr><td>MT6</td><td>P + O + glucuronide</td><td>0.01</td><td>ND</td><td>0.14</td><td>1.1</td><td>0.06</td></tr>
<tr><td>MT7</td><td>P + O + glucuronide</td><td>ND</td><td><0.01</td><td>1.89</td><td>ND</td><td>0.12</td></tr>
<tr><td>MT8</td><td>P + O + glucuronide</td><td>0.29</td><td>ND</td><td>1.18</td><td>5.5</td><td>0.01</td></tr>
<tr><td>MT9</td><td>P + O + glucuronide</td><td>0.05</td><td><0.01</td><td>1.48</td><td>0.1</td><td><0.01</td></tr>
<tr><td>MT10</td><td>P + O + glucuronide</td><td>ND</td><td>0.04</td><td>12</td><td>0.5</td><td>1.61</td></tr>
</table>

Scheme S1. Proposed pathways and structures of **VU6008055** metabolites.

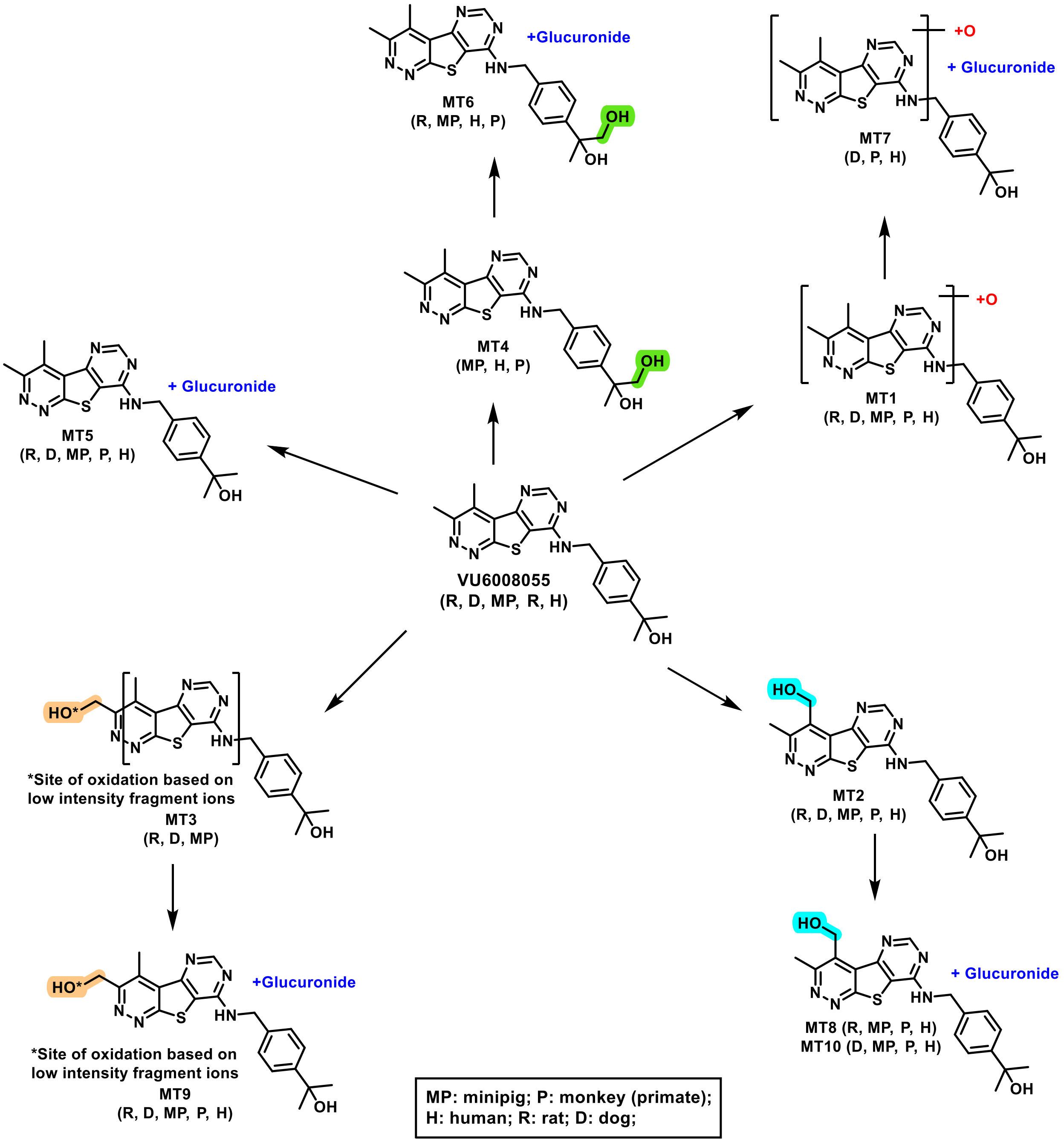

Scheme S2. **VU6008055** Fragmentation Pathways.

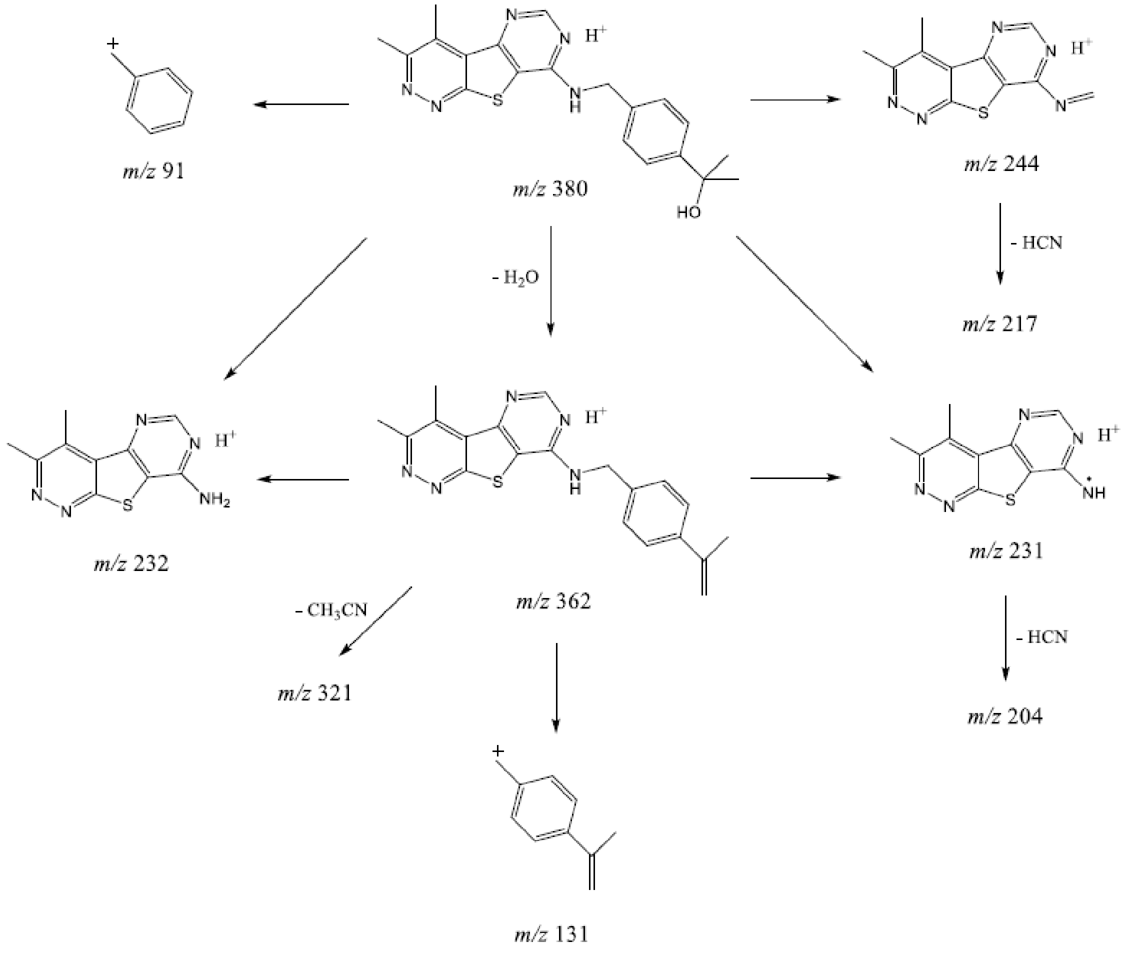

ADME-Tox: Genetic Toxicity

Table S13. Bacterial Cytotoxicity Profile of **VU6008055**

Compound I.D.	Client Compound I.D.	Test Concentration	%Effect 1st	2nd	3rd	Mean %Effect	Cytotoxicity (% of control)	Flags 1st	2nd	3rd
**Bacterial cytotoxicity (TA98 - S9)**										
100032130-1	VU6008055	6.0E-07 M	94.8	92.0	93.9	**93.6**	94			
100032130-1	VU6008055	1.2E-06 M	96.6	103.1	99.4	**99.7**	100			
100032130-1	VU6008055	2.5E-06 M	87.4	99.4	96.6	**94.5**	94			
100032130-1	VU6008055	5.0E-06 M	99.4	104.0	101.2	**101.6**	102			
100032130-1	VU6008055	1.0E-05 M	103.1	104.0	102.2	**103.1**	103			
100032130-1	VU6008055	2.5E-05 M	96.6	126.1	97.6	**106.8**	107			
100032130-1	VU6008055	5.0E-05 M	133.5	115.1	134.4	**127.6**	128			
100032130-1	VU6008055	1.0E-04 M	118.7	139.0	143.6	**133.8**	134			
**Bacterial cytotoxicity (TA100 - S9)**										
100032130-1	VU6008055	6.0E-07 M	96.6	99.8	93.5	**96.6**	97			
100032130-1	VU6008055	1.2E-06 M	97.7	91.4	94.5	**94.5**	95			
100032130-1	VU6008055	2.5E-06 M	93.5	94.5	100.8	**96.3**	96			
100032130-1	VU6008055	5.0E-06 M	84.0	94.5	94.5	**91.0**	91			
100032130-1	VU6008055	1.0E-05 M	90.3	98.7	104.0	**97.7**	98			
100032130-1	VU6008055	2.5E-05 M	99.8	94.5	144.9	**97.1**	97			{}
100032130-1	VU6008055	5.0E-05 M	97.7	99.8	131.3	**109.6**	110			
100032130-1	VU6008055	1.0E-04 M	143.9	160.7	167.0	**157.2**	157			
**Bacterial cytotoxicity (TA1535 - S9)**										
100032130-1	VU6008055	6.0E-07 M	99.3	97.3	98.3	**98.3**	98			
100032130-1	VU6008055	1.2E-06 M	102.4	98.3	90.2	**97.0**	97			
100032130-1	VU6008055	2.5E-06 M	97.3	96.3	93.2	**95.6**	96			
100032130-1	VU6008055	5.0E-06 M	92.2	96.3	93.2	**93.9**	94			
100032130-1	VU6008055	1.0E-05 M	95.3	100.3	95.3	**97.0**	97			
100032130-1	VU6008055	2.5E-05 M	95.3	94.3	122.6	**104.1**	104			
100032130-1	VU6008055	5.0E-05 M	103.4	142.9	116.6	**120.9**	121			
100032130-1	VU6008055	1.0E-04 M	136.8	142.9	144.9	**141.6**	142			
**Bacterial cytotoxicity (TA1537 - S9)**										
100032130-1	VU6008055	6.0E-07 M	100.4	100.4	100.4	**100.4**	100			
100032130-1	VU6008055	1.2E-06 M	99.3	96.1	95.1	**96.8**	97			
100032130-1	VU6008055	2.5E-06 M	98.2	96.1	95.1	**96.5**	96			
100032130-1	VU6008055	5.0E-06 M	95.1	96.1	97.2	**96.1**	96			
100032130-1	VU6008055	1.0E-05 M	93.0	99.3	93.0	**95.1**	95			
100032130-1	VU6008055	2.5E-05 M	109.9	96.1	133.1	**113.0**	113			
100032130-1	VU6008055	5.0E-05 M	112.0	127.8	124.6	**121.5**	121			
100032130-1	VU6008055	1.0E-04 M	187.0	172.2	185.9	**181.7**	182			

Notes:
1. Cytotoxicity is presented as % of control growth.
2. A cytotoxicity value of less than 60 % is flagged, and the compound is considered as toxic at the respective concentration.

{}: That replicate was excluded from the calculation

Table S14. Ames Test with **VU6008055**

Compound I.D.	Client Compound I.D.	Test Concentration	Count (# of wells)	Positive Significance (- to +++)	Fisher Exact Test (p-value)	Count Flag
**Ames fluctuation test (TA98 - S9)**						
100032130-1	VU6008055	5.0E-06 M	0	-	1.0000	
100032130-1	VU6008055	1.0E-05 M	0	-	1.0000	
100032130-1	VU6008055	5.0E-05 M	0	-	1.0000	
100032130-1	VU6008055	1.0E-04 M	1	-	0.5000	
**Ames fluctuation test (TA98 + S9)**						
100032130-1	VU6008055	5.0E-06 M	2	-	1.0000	
100032130-1	VU6008055	1.0E-05 M	3	-	0.5000	
100032130-1	VU6008055	5.0E-05 M	3	-	0.5000	
100032130-1	VU6008055	1.0E-04 M	4	-	0.3387	
**Ames fluctuation test (TA100 - S9)**						
100032130-1	VU6008055	5.0E-06 M	0	-	0.5000	-
100032130-1	VU6008055	1.0E-05 M	1	-	1.0000	
100032130-1	VU6008055	5.0E-05 M	2	-	0.5000	
100032130-1	VU6008055	1.0E-04 M	1	-	1.0000	
**Ames fluctuation test (TA100 + S9)**						
100032130-1	VU6008055	5.0E-06 M	12	-	1.0000	-
100032130-1	VU6008055	1.0E-05 M	9	-	0.3111	-
100032130-1	VU6008055	5.0E-05 M	7	-	0.1528	-
100032130-1	VU6008055	1.0E-04 M	6	-	0.0951	-
**Ames fluctuation test (TA1535 - S9)**						
100032130-1	VU6008055	5.0E-06 M	1	-	0.5000	
100032130-1	VU6008055	1.0E-05 M	0	-	1.0000	
100032130-1	VU6008055	5.0E-05 M	0	-	1.0000	
100032130-1	VU6008055	1.0E-04 M	1	-	0.5000	
**Ames fluctuation test (TA1535 + S9)**						
100032130-1	VU6008055	5.0E-06 M	1	-	1.0000	-
100032130-1	VU6008055	1.0E-05 M	0	-	0.5000	-
100032130-1	VU6008055	5.0E-05 M	0	-	0.5000	-
100032130-1	VU6008055	1.0E-04 M	2	-	0.5000	
**Ames fluctuation test (TA1537-S9)**						
100032130-1	VU6008055	5.0E-06 M	0	-	0.5000	-
100032130-1	VU6008055	1.0E-05 M	0	-	0.5000	-
100032130-1	VU6008055	5.0E-05 M	0	-	0.5000	-
100032130-1	VU6008055	1.0E-04 M	1	-	1.0000	-
**Ames fluctuation test (TA1537 + S9)**						
100032130-1	VU6008055	5.0E-06 M	1	-	1.0000	
100032130-1	VU6008055	1.0E-05 M	1	-	1.0000	
100032130-1	VU6008055	5.0E-05 M	1	-	1.0000	
100032130-1	VU6008055	1.0E-04 M	2	-	0.5000	

Notes:
1. Weak positive, if p < 0.05, denoted as "+"
   Strong positive, if p < 0.01, denoted as "++"
   Very strong positive, if p < 0.001, denoted as "+++"
2. When possible, compounds which score significantly below background are flagged.
   This may indicate low level cytotoxicity undetectable by the growth assay.
   The compounds are flagged as described below.
   if p < 0.05, flagged as "<"
   if p < 0.01, flagged as "<<"
   if p < 0.001, flagged as "<<<"
3. Hyphens (-) indicate negative results.

***In vivo* Behavioral Pharmacology**

Animals

Adult male Sprague Dawley rats from Envigo (Indianapolis, IN) were used for all behavioral studies. All experiments were approved by the Institutional Animal Care and Use Committee at Vanderbilt University and experimental procedures followed the guidelines of the National Research Council Guide for the Care and Use of Laboratory Animals.

VU6008055 Rat AHL Protocol

Adult male Harlan Sprague Dawley rats were tested in SmartFrame Open Field locomotor activity test chambers (Kinder Scientific, Poway, CA) to automatically record locomotor activity. All rats were habituated in locomotor activity enclosures for 30 min, followed by pretreatment by oral gavage for an additional 30 min with either vehicle or a dose of **VU6008055** or the comparator M_4 PAM **VU0467154**. Next, rats were injected subcutaneously with vehicle or a dose of 0.75 mg/kg amphetamine and then monitored for an additional 60 min. Changes in locomotor activity were recorded for a total of 120 min. Locomotor data were expressed as the number of photobeam breaks/5 min intervals across the 120-min test session or as the total ambulation, calculated as sum of photobeam beam breaks from the time of amphetamine administration (60 min) until the end of the study (120 min). Time course data were analyzed by two-way ANOVA with main effects of treatment and time; changes in total ambulation were analyzed by one-way ANOVA followed by Dunnett's *post hoc* test (GraphPad Prism 7 [GraphPad Software, San Diego, CA]). For all tests, $\alpha \leq 0.05$ was considered to represent statistical significance. Finally, percent reversal data were calculated in Microsoft Excel using the following formula: Percent Reversal = 100 - {[(total ambulation in individual animal from t = 60 to t = 120) / (mean total ambulation from t=60 to t=120 in the VAMP group)] * 100}. Mean percent reversal ± S.E.M. was calculated for each dose group using GraphPad Prism 7.

At the end of this behavioral study (90 min after compound administration, rats were anesthetized with isoflurane, then decapitated, and the plasma and brain tissues were collected for the evaluation of exposure levels of **VU6008055** or **VU0467154** by pharmacokinetic analysis.

VU6008055 Rat MK-801-Induced Hyperlocomotion Protocol

The methods for assessing the effects of **VU6008055** on MK-801-induced hyperlocomotion were similar to those for the AHL study with the *following exceptions*: Following the 30-min habituation in the locomotor chambers, rats were pretreated by oral gavage with vehicle, a dose of **VU6008055** or the comparator M_4 PAM **VU0467154**. *60 min later* animals were injected subcutaneously with MK-801 (0.18

mg/kg) and then monitored for an additional 60 min. Changes in locomotor activity were recorded for a total of *150 min*. Locomotor data were expressed as the number of photobeam breaks/5 min intervals across the 150-min test session or as the total ambulation, calculated as sum of photobeam beam breaks from the time of MK-801 administration *(90 min)* until the end of the study *(150 min)*. Time course data were analyzed by two-way ANOVA with main effects of treatment and time; changes in total ambulation were analyzed by one-way ANOVA followed by Dunnett's *post hoc* test (GraphPad Prism 7 [GraphPad Software, San Diego, CA]). For all tests, $\alpha \leq 0.05$ was considered to represent statistical significance. Finally, percent reversal data were calculated in Microsoft Excel using the following formula: *Percent Reversal = 100 - {[(total ambulation in individual animal from t = 90 to t = 150) / (mean total ambulation from t=90 to t=150 in the VAMP group)] * 100}*. Mean percent reversal ± S.E.M. was calculated for each dose group using GraphPad Prism 7.

Conditioned Avoidance Response

Two batches of sixty-four male Wistar Han rats (ordered to be in the range of 125-150g upon arrival) were purchased from Charles River, Margate, UK. One batch was used for studies with **VU6008055** alone and one for studies with **VU608055** in combination with risperidone. Animals were group housed in cages of 4 upon arrival with free access to food and water. All animals were maintained at 21±2 °C on a normal phase light-dark cycle (lights on approx. 07:00 – 19:00 h). Relative humidity was typically 55±15% with prolonged periods below 40%RH or above 70%RH avoided as detailed in the UK Code of Practice. Animals were acclimatized for approximately one week prior to the start of the training.

Conditioned avoidance behavior was assessed using automated shuttle boxes (42 x 16 x 20 cm; Med Associates, St. Albans USA). Boxes were subdivided into two compartments by means of a partition (through which rats could pass) and the presence of infrared-sensitive photocells enabled the position of the animals to be determined. Each compartment had a separate light and tone generator. Each box was placed in a sound attenuated chamber. Animals were trained to avoid a foot shock. Specifically, animals were trained to move to the adjacent compartment within 10 s upon administration of the conditioned stimulus (tone and light), in order to avoid presentation of the unconditioned stimulus (foot shock, 0.5 mA for a maximum duration of 10 s) via the grid floor.

Training was organized such that the 64 rats each underwent two weeks of training (Monday - Friday) consisting of 30 trials in a 30 min test session with a variable inter-trial interval of 20-30 s. Where an animal crossed to the other compartment to avoid the shock, this was recorded by the apparatus as an avoidance response. Where the animal crossed to the other compartment during presentation of the shock, this was recorded by the apparatus as an escape response. Where the animal did not cross to the other compartment

during presentation of the shock, this was recorded by the apparatus as an escape failure. Subsequent to the ten days training of 30 daily trials, the final five days of training was reduced to 10 trials per day (a 10-minute test session with a variable inter-trial interval of 20-30 s). Eleven animals were removed from the study by the end of training because they did not meet the criteria for inclusion. Animals included for testing were tested at weekly intervals with the study week organized such that two training days (each 10 trials) were followed by a baseline (pre-test) session. In this session, all animals were dosed with the study vehicles prior to the test by either the subcutaneous (risperidone, 30 min prior to test) or oral (**VU6008055**, 60 min prior to test) route. Animals then underwent the test protocol (10 trials in a 10 min session with a variable inter-trial interval of 20-30 s). Animals that exhibited stable performance (>80% avoidance responses for last 3 drug-free CAR sessions), underwent drug (or vehicle) testing the next day. Any animals that did not meet the success criteria underwent further training on the day of the experiment. The allocation of animals to experimental treatments was based on performance in the baseline session, such that groups were balanced as far as possible for number of avoidance responses, latency to avoid or escape and testing chamber.

Pharmacologic MRI

Experimental design. Male Sprague Dawley rats were scanned to collect pharmacological MRI data using a 9.4 Tesla Varian magnet controlled by a Varian Inova console with a Doty litz 38-mm transmit-receive radiofrequency coil.[2] For preparation, rats with preimplanted jugular vein catheters were weighed and received pretreatment by oral gavage with either vehicle or a dose of **VU6008055** (1 and 10 mg/kg), then anesthetized with isoflurane (3.5%) for 4 minutes, and were intubated. After transfer to a head holder with a bite bar and connection to a mechanical ventilator (O_2:N_2O 1:2 in 2% isoflurane, respiratory rate 80 breaths per minute), the jugular catheter was flushed with saline, physiological monitoring probes (Electrocardiogram, rectal temperature, end tidal carbon dioxide physiological parameters were monitored during the experiment, rectal temperature, end tidal carbon dioxide) and two subcutaneous catheters were placed. Isoflurane was lowered to 0.9%, drug lines were connected for pancuronium bromide and amphetamine, and after 20 minutes, toe/tail pinch withdrawal and corneal reflexes were tested. After determining lack of reflexes, pancuronium bromide (1 mg/kg, sc) administered. During the next 25 minutes, the rat was placed in the scanner and MRI set-up (coil tuning and matching, power calibration, shimming) took place, as well as slice selection and collection of anatomical images. High-resolution anatomical images were acquired using a fast spin-echo sequence with the following parameters: repetition time 2550 ms; effective echo time 40 ms; number of excitations 2; 128 × 128 matrix; 35 × 35 mm2 field of view; 14 contiguous slices, 1.0 mm thick). Respiratory rate was adjusted to 66 breaths per minute; heart rate,

respiration, and rectal temperature were continuously monitored and temperature maintained through an air-heating unit. End-tidal CO_2 was continuously monitored and, if it changed, maintained by adjusting flow rate. The functional scan consisted of a 5 minute pre-contrast baseline, contrast agent (monocrystalline ion oxide nanoparticoles, MION 20 mg/kg, iv) injection and 5 minute equilibration and 10 minute post-contrast baseline followed by subcutaneous injection of vehicle or 1 mg/kg amphetamine (85 minutes after pretreatment) and additional 45 minutes of image acquisition. Contrast-enhanced CBV fMRI data were acquired using a fast spin echo sequence (repetition time 2000 ms; effective echo time 36 ms; number of excitations 3; 64 × 64 matrix) with the slice plan copied from the anatomical scan protocol; one brain volume (14 slices) was acquired every minute. At the end of each fMRI study, isoflurane was increased to 5% for euthanization, and the anatomical scan was repeated to determine that contrast agent had entered the brain; then each animal was removed from the scanner/holder and decapitated for plasma and brain tissue collection for the evaluation of exposure levels of **VU6008055** by pharmacokinetic analysis.

phMRI Data Analysis. Raw data were images reconstructed by the Varian Inova software. To express data as percent change in cerebral blood volume over baseline per minute, images were preprocessed and analyzed using in-house MATLAB code and Analysis of Functional NeuroImages (AFNI) software3. Preprocessing included brain-masking, motion-correction (AFNI 2dreg), and coregistration to a template (AFNI 3dreg). Fractional CBV changes were calculated on a voxel-wise basis for each subject for each image using the equation: $\%\Delta CBV(t)/CBVo = [\ln S(t) - \ln So]/[\ln So - \ln Spre] \times 100$, where S(t) is the measured signal at time t, So is the post-contrast baseline signal, and Spre is the pre-contrast baseline4 and converted to percentages. Data were expressed as percent change in cerebral volume over baseline. Regions of interest (ROIs), pre-defined on the template, based on the Paxinos and Watson rat brain atlas (2007), were applied to all coregistered subjects. Mean percent CBV changes (left and right hemispheres averaged) were calculated for each ROI for each minute to generate time courses. The mean percent change in CBV were calculated for each ROI for minutes 31-40 for each treatment group; these mean CBV changes (31-40 minute bin) were statistically compared between 1-A versus V-A and 10-A vs V-A using one-way ANOVA and Dunnett's post hoc multiple comparison test in GraphPad Prism 8.0. Mean CBV changes were calculated for the 31-40 minute bin for the V-V versus **VU6008055** 10-V groups were statistically compared using the Mann Whitney test (one-tailed) in GraphPad Prism 8.0. Maps were generated using mean percent change in CBV for minutes 31-40 and corresponding voxels of the template brain colorized.

EEG Procedures

Surgery. All animals used in telemetry studies were surgically implanted under isoflurane anesthesia with a telemetric transmitter (Data Sciences International [DSI], Minneapolis, MN) for the wireless recording

of EEG, electromyographic (EMG), and motor activity as described previously.[3] After surgery animals were individually housed.

Electroencephalography. All animals were allowed post-surgical recovery for at least 10 days before EEG recordings were initiated. EEG and EMG were recorded from the home cage of each animal continuously for 24 hrs beginning at the onset of the light cycle on the day of each study. Telemetric EEG and EMG waveform data were collected using Dataquest A.R.T. software (DSI). Data were continuously sampled at a rate of 500 Hz and transmitted via a receiver (RPC-1, DSI) placed below the cage of each rat. Each receiver was connected to a data exchange matrix (DSI, MN), which transfers data to a computer for off-line analysis.

qEEG spectral power analysis. Power spectra were computed in 1-Hz bins from 0.5 to 100 Hz using a Fast Fourier Transform with a Hamming window and overlap ratio of 0.5 for each rat in 10-sec epochs. Spectral power across the entire spectrum was examined by averaging the power from all 10-sec wake epochs to yield the state-dependent relative power spectrum in 1-Hz intervals as previously described.[3,4] Pharmacological effects on arousal during wake were determined as within subject changes by expressing the power spectrum 1-2 hour post dosing as a percent change within each respective 1 Hz interval from the 1-h interval prior to dosing (baseline). (e.g. [power 1-2 h post dosing/ 1 h baseline x 100]-100). Data from each animal were then averaged.

Statistics. qEEG data are presented as means ± S.E.M. Dose- and frequency band -dependent effects of VU6008055 were analyzed by a mixed model ANOVA followed by Dunnett's *post hoc* test to compare the vehicle controls to each dose of **VU6008055** using GraphPad Prism.

Human Dose Predictions

A prediction of hepatic metabolic clearance was also made using the well-stirred liver model, incorporating established physiological scaling factors as well as unbound fractions in blood and non-specific binding within the in vitro hepatocyte incubation and inclusion of an empirical correction factor.[5,6] The empirical correction factor was based on a regression equation derived for a set of reference compounds for which the main route of elimination was reported as hepatic metabolism (currently based on prior knowledge, Table S16). A schematic in Figure S4, shows the steps to transform the in vitro hepatocyte CL_{int} to a predicted in vivo CL using the regression correction approach.[7,8]

The *in vitro* CL_{int} is scaled to a liver CL_{int} according to Equation 1.

$$Scaled\ CL_{\text{int}}\ \frac{CL_{\text{int}} \cdot SF \cdot fu_b}{fu_{inc}} \quad (1)$$

Where fu_b and fu_{inc} are given by equations 2 and 3 respectively and SF have been presented in Table S15.

$$fu_b = \frac{fu_p}{R_b} \qquad \text{whereby } R_b = \text{the blood to plasma ratio} \quad (2)$$

$$fu_{inc} = \frac{1}{1 + 125 \cdot V_R \cdot 10^{0.072 \cdot \log P/D^2} + 0.067 \cdot \log P/D - 1.126} \quad (3)$$

Whereby V_R = the ratio between the cell volume and the incubation volume and it has a value of 0.005 at a cell concentration of $1E^6$ cells/mL.[9] The lipophilicty descriptor, log P / D, corresponds to a logP value for basic compounds and a $logD_{7.4}$ value for all other ion classes (e.g. acidic, neutral or zwitterionic compounds).

The slope and intercept from the regression offset equation were then used to calculate

the log predicted *in vivo* CL_{int}, that is, log[(CL_b × LBF)/ (LBF-CL_b)]. The predicted blood clearance was then calculated after rearrangement of the WSM according to equation 4.

$$\text{Predicted blood CL} = \frac{\text{LBF} \cdot \textit{predicted}\ \text{CL}_{\text{int, in vivo}}}{\text{LBF} + \textit{predicted}\ \text{CL}_{\text{int,in vivo}}} \quad (4)$$

Whereby the predicted $CL_{\text{int, in vivo}}$ is given by: $10^{\log(\text{predicted CL}_{\text{int,in vivo}})}$ (5)

The above predicted hepatic metabolic clearance plus **AF98943 (VU6008055)** specific properties (e.g. logD, pKa, fu_p) were integrated with system defined physiological values (e.g. tissue volumes and blood flows) in order to simulate total plasma-concentration time profiles using Gastro-Plus v9.6; a commercial physiologically-based pharmacokinetic modelling software. Following IV and oral dosing simulated and observed plasma-concentration time profiles were compared for rat, beagle dog, cynomolgus monkey and Göttingen minipig to substantiate the PBPK modelling approach. This approach was then applied to human *in vitro* data to prospectively predict human PK parameters and profiles at specified doses. The principal input parameters for PBPK modelling of **AF98943 (VU6008055)** are outlined in Table S16. Simulations were run using rat, dog, monkey, minipig and human default whole body PBPK models customized to align with standardized liver blood flow values and representative animal body weights (e.g. mean values from PK studies where applicable). Perfusion rate-limited kinetics were assumed. V_{ss} was predicted using the recommended mechanistic tissue composition model and adjusted fu_p values calculated within Gastro-Plus.[10] The liver and kidney were considered the only sites of elimination (renal CL was calculated

according to GFR • fu_p). With regards simulating oral PK profiles, an in-silico P_{eff} value (predicted from structure using the Gastro-Plus ADMET predictor add-in was used in conjunction with the measured thermodynamic solubility to calculate the rate constants used in the Gastro-Plus multi-compartment ACAT model that defines the oral absorption profile.[11,12] All other software variables were set according to the recommended Gastro-Plus default settings. Simulated IV and oral PK profiles for rat, dog, monkey and minipig were run using either the observed CL, taken from the PK studies, or predicted CL scaled from hepatocyte CL_{int} data. Using the observed CL facilitated a more in-depth evaluation of how accurately the V_{ss} and oral absorption profiles were predicted from the tissue composition model and consideration of permeability and measured solubility, respectively. Comparing simulated profiles using the predicted CL with the *in vivo* profiles provided an overall view of how accurately PK could be predicted from combination of IVIVE of CL and PBPK modelling.

Dose predictions are based on achieving transient cover (12 hr) above a pharmacological target, total human plasma concentration of 11 µM when corrected for fraction unbound (rat and human).

The human therapeutic dose (D) is estimated by considering the human PK prediction and the steady-state pharmacologically active therapeutic concentration (C_{eff}) using equation:

$$\text{Predicted human dose} = \left[\frac{C_{eff}\cdot AR\cdot\left(k_a - k_{e\lim}\right)\cdot V_{ss}}{k_a\cdot\left(e^{-k_{e\lim}\cdot tau} - e^{-k_a\cdot tau}\right)}\right] \Big/ F \qquad (6)$$

Where,

C_{eff} = steady-state effective concentration

AR = accumulation ratio = $1 - e^{-K_{elim}}$. tau

Tau = dosing interval

k_{elim} = elimination rate

k_a = absorption rate (estimated from the oral T_{max} in the animal PK models; note if a PBPK model is used the absorption rate is calculated within the Gastro-Plus software for each GI compartment described within the ACAT model).

V_{ss} = steady-state volume of distribution

F = bioavailability = $F_a . F_g . F_h$

Based on the *in vitro-in vivo* clearance extrapolation (IVIVE) scaling approach and considering a human target total steady-state plasma concentration of 4200 ng/mL (1800 ng/mL in pharmacological species), the predicted human dose is 820 mg for once daily and 640 mg for twice daily administration. These predictions are well aligned with the physiologically based pharmacokinetic modelling (PBPK) model, where the predicted human efficacious dose is 850 mg for once daily or 410 for twice daily administration. Hence, the overall predicted human dose is 820-850 mg once daily or 410-640 mg twice daily (**Table S18**).

Figure S4. Prediction of hepatic metabolic CL. Reference compounds were used to establish regression correction factor expressed as the following equation: $\log_{10}$[(derived *in vivo* CL_{int} = a • $\log_{10}$(CL_{int} • SF • (fu_b/fu_{inc}) + b; where a = the slope and b = the intercept. This was subsequently used to predict *in vivo* CL_{int} and CL for the SQ compound. Note, the derived *in vivo* CL_{int} is calculated from the total *in vivo* CL using the rearranged well-stirred model according to: (CL·LBF)/(LBF-CL).

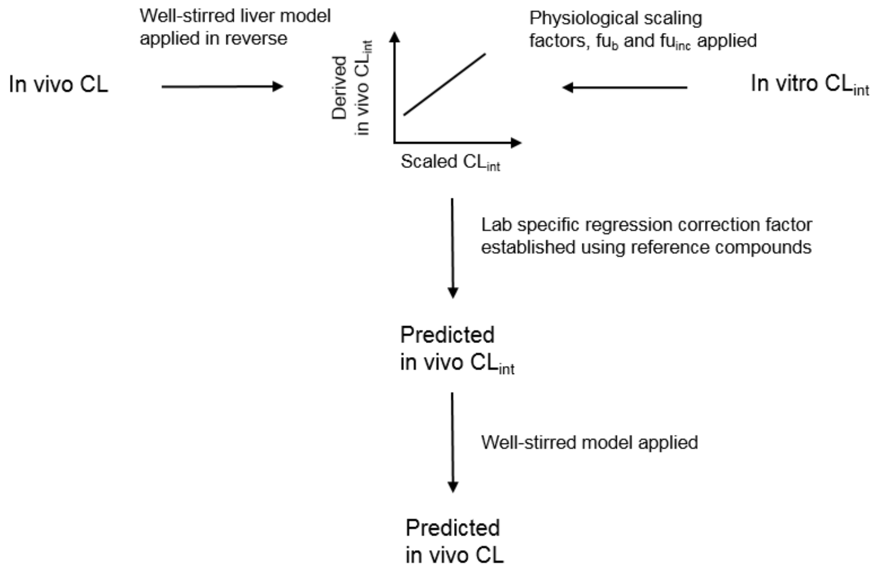

Table S15. Parameters for IVIVE using the modified well-stirred liver model. The empirical scaler for each species was identified by regression analysis applied to a reference set of compounds.

Parameter	Rat	Dog	Monkey	Minipig	Human
**Physiological scaling factor**					
Hepatocytes 1E^{6} / g liver	163	169	120	124	120
Liver weight g / kg body weight	40	32	32	16.7	24
LBF (L/h/kg)	4.8	3.3	2.64	2.22	1.2
**Linear regression equations***					
Hepatocyte CL_{int} based assay	y = 0.76x + 0.48	y = 0.67x + 0.52	y = 0.73x + 0.48	y = 0.76x + 0.48	y = 0.73x + 0.48

*Based on prior knowledge

Table S16. Compound and System specific parameters used within Gastro-Plus PBPK software to predict V_{ss} and simulate IV and oral PK profiles.

Parameter	Rat	Dog	Monkey	Minipig	Human
**Compound specific**					
logD	3.5				
pKa	3.56, 2.73, 0.57, -0.89, -1.71				
MDCK-MDR1 A2B P_{app} / in silico P_{app} (E^{-6} cm/s)	9 / 42				
Predicted P_{eff} (E^{-4} cm/s)*	0.21	2.6	0.93	0.81	0.94
Thermodynamic solubility, SGF, FaSSIF, FeSSIF (mg/mL)	0.0015, 0.13, 0.0077, 0.037				
Thermodynamic solubility used in simulations	0.015	0.003	0.0045	0.0015	0.025
In vitro fu_p (% free)**	0.78	3.8	0.76	1.63	0.42
Adjusted fu_p (% free)***	0.74	2.2	0.66	1.2	0.39
R_b****	0.66	1.05	1.05	1.05	1.05
Predicted plasma $CL_{hepatic, metabolic}$ (L/h/kg)	0.19	0.70	0.19	0.19	0.046
Predicted CL_{renal} according to $fu_p \cdot GFR$ (L/h/kg)*****	0.004	NA	0.001	0.002	0.0004
In vivo plasma CL_{total} (L/h/kg)	0.26	0.96	0.32	0.46	
**Gastro-Plus system specific**					
Whole body animal physiology model	Rat_0.25kg_ 80mL/min/Kg_LBF	Compartmental	Monkey_4kg_ 44mL/min/Kg_LBF	Minipig_14.2kg_ 37mL/min/Kg_LBF	HumAmeMalHlthy30YO_70kg_LBF_20.5ml/min/Kg
LBF (L/h/kg)	4.8	3.3	2.64	2.22	1.2
Whole body tissue composition (Kp) model	Perfusion limited**Error! Reference source not found.**				
Oral physiology model	Rat - Fasted	Beagle - Fasted	Human - Fasted	Minipig - Fed	Human - Fasted
Oral absorption model	Opt logD Model SA/V6.1				

* Predicted from structure using Gastro-Plus ADMET predictor in-silico model

** In vitro fu_p is given as the average across test concentrations in Table

*** Calculated within the Gastro-Plus software to account for observations whereby tissue K_ps and hence V_{ss} can be over-predicted for lipophilic drugs. Investigators have shown this can be corrected using an adjusted fu_p value and hypothesize the difference in adjusted versus measured fu_p may be a consequence of significant binding to plasma lipids (and may not be captured by standard fu_p equilibrium dialysis measurements).

**** Blood to plasma ratio derived from in vivo PK studies in which blood and plasma AUC's were determined in the same animals.

***** Renal CL was predicted within Gastro-Plus based on $fu_p \bullet GFR$

Table S17. In vitro plasma protein binding of **AF98943 (VU6008055)** in preclinical species and human at plasma concentrations of 0.1, 1.0 and 10 µM (mean ± SD, n=3, N=1).

**Species**	**Dilution**	**Concentration (µM)**		
		**0.1**	**1.0**	**10**
Human (% free)	10	0.43 ± 0.22	0.40 ± 0.07	0.43 ± 0.19
C57 Mouse (% free)	10	<0.2	0.35 ± 0.11	0.32 ± 0.01
SD Rat (% free)	10	<0.5	0.97 ± 0.08	0.59 ± 0.08
Cynomolgus Monkey (% free)	10	0.76 ± 0.04	0.70 ± 0.05	0.82 ± 0.25
Göttingen Minipig (% free)	10	1.5 ± 0.2	1.5 ± 0.1	1.9 ± 0.9
Beagle Dog (% free)	1	3.4 ± 0.2	4.1 ± 0.8	3.9 ± 0.1

Table S18. Summary of human PK and dose predictions.

**Predicted parameter**	**Human, IVIVE approach**	**Human, PBPK approach QD (BID)**	**Human predictions /overall classification**
Plasma CL_{hep} (L/h/kg)	0.046	0.046	Low clearance
Plasma V_{ss} (L/Kg)	0.44	0.74	Low-moderate volume of distribution
Plasma $T_{1/2}$ (h)	6.6	11.5	Moderate half-life
Oral F_{abs} (%)	~50	40 (47)	Solubility limited absorption - dose dependent
Oral F_g (%)	100	100	Assumed there is no intestinal first pass metabolism
F_{oral} (%)	48	38 (45)	Moderate-high bioavailability
Human target total plasma conc (ng/mL)	4200 (11162 nM)	4200 (11162 nM)	Based on rodent in vivo pharmacology models, corrected for M4 EC_{50} and free fraction in plasma

Once Daily Dose (mg)	820	850*	820-850 mg once daily
Twice Daily Dose,<br>12 hr apart (mg)	640	410**	410-640 mg twice daily

Footnote: * Assumed solution dosing (immediate release capsule) under fasted conditions for first in human. For PBPK model, Fabs is very sensitive to input solubility value for proposed dose range. Applied an empirical correction factor of 16-fold (0.024 mg/ml) to the thermodynamic solubility pH7.4 (based on rat oral PBPK model). Projected doses should therefore be interpreted with caution and have greater validity under enabling formulation conditions where in vivo solubility can be anticipated in the defined range (or an appropriate dissolution profile if administering as a suspension or tablet). ** BID facilitates a lower dose (with a higher associated $F_{abs,}$).

References

1) Lin, J.H.; Chiba, M.; Balani, S.K.; Chen, I.W.; Kwei, G.Y.; Vastag, K.J; Nishime, J.A. Species differences in the pharmacokinetics and metabolism of indinavir, a potent human immunodeficiency virus protease inhibitor. *Drug Metab Dispos*. 1996; 24:1111-1120.
2) Byun, N.E.; Grannan, M.; Bubser, M.; Barry, R.L.; Thompson, A.; Rosanelli, J.; Gowrishankar, R.; Kelm, N.S.; Damon, S.; Bridges, T.; Melancon, B.J.; Tarr, J.C.; Brogan, J.T.; Avison, M.J.; Deutch, A.; Wess, J.; Wood, M.R.; Lindsley, C.W.; Gore, J.C.; Conn, P.J.; Jones, C.K. Antipsychotic-like and cognitive enhancing effects of the selective M4 muscarinic acetylcholine receptor positive allosteric modulator VU0152100. *Neuropsychopharmacology*. 2014; 39:1578-1593.
3) Gould, R.W.; Nedelcovych, M.T.; Gong, X.; Tsai, E.; Bubser, M.; Bridges, T.M.; Wood, M.R.; Duggan, M.E.; Brandon, N.J.; Dunlop, J.; Wood, M.W.; Ivarsson, M.; Noetzel, M.J.; Daniels, J.S.; Niswender, C.M.; Lindsley, C.W.; Conn, P.J.; Jones, C.K. State-dependent alterations in sleep/wake architecture elicited by the M4 PAM VU0467154 – Relation to antipsychotic drug-like effects. *Neuropharmacology*. 2016; 102:244-253.
4) Gould, R.W.; Russell, J.K.; Nedelcovych, M.T.; Bubser, M.; Blobaum, A.L.; Bridges, T.M.; Newhouse, P.A.; Lindsley, C.W.; Conn, P.J.; Nader, M.A.; Jones, C.K. Modulation of arousal and sleep/wake architecture by M1 PAM VU0453595 across young and aged rodents and nonhuman primates. *Neuropsychopharmacology*. 2020; 45:2219-2228.
5) Smith, R.; Jones, R.D.O.; Ballard, P.G.; Griffiths, H.H. Determination of microsome and hepatocyte scaling factors for in vitro/in vivo extrapolation in the rat and dog. *Xenobiotica*. 2008; 38: 1386-1398
6) Riley, R.J.; McGinnity, D.F.; Austin, R.P. A unified model for predictin human hepatic, metabolic clearance from in vitro intrinsic data in hepatocytes and microsomes. *Drug Metab. Dispos*. 2005; 33: 1304-1311.

7) Sohlenius-Sternbeck, A.K.; Afzelius, L.; Prusis, P.; Neelissen, J.; Hoogstraate, J.; Johansson, J.; Floby, E.; Bengtsson, A.; Gissberg, O.; Sternbeck, J.; Petersson, C. Evaluation of the human prediciton of clearance from hepatocyte and microsome intrinsic clearance for 52 drug compounds. *Xenobiotica*. 2010; 40: 637-649.
8) Sohlenius-Sternbeck, A.K.; Jones, C.; Ferguson, D.; Middleton B.J.; Projean, D.; Floby, E.; Bylund, J.; Afzelius, L. Practical use of the regression offset approach for the prediction of in vivo intrinsic clearance from hepatocytes. *Xenobiotica*. 2012; 42: 841-853.
9) Kilford, P.J. Hepatocellular binding of drugs: correction for unbound fraction I hepatocyte incubations using microsonal binding or drug lipophilicty data. *Drug Metab. Dispos*. 2008; 36: 1194-1197.
10) Lukacova, V.P.N.; Lave, T.; Fraczkiewicz, G.; Bolger, M.B.; Woltosz, W.S. 2008. Role of fraction unbound in plasma in calculations of tissue: plasma partition coefficients. 2008 AAPS National Meeting; Atlanta, Georgia.
11) Gertzj M.; Harrison, A.; Houston, J.B.; Galetin, A. Prediction of human intestinal first-pass metabolism of 25 CYP3A sub-strates from in vitro clearance and permeability data. *Drug Metab. Dispos*. 2010; 38:1147-1158.
12) Lennerna, H. Human in vivo regional intestinal permeability : importance for pharmaceutical drug development. *Mol. Pharmaceutics*. 2014; 11:12-23.
